# Host-range shift of H3N8 canine influenza virus: a phylodynamic analysis of its origin and adaptation from equine to canine host

**DOI:** 10.1186/s13567-019-0707-2

**Published:** 2019-10-30

**Authors:** Wanting He, Gairu Li, Ruyi Wang, Weifeng Shi, Kemang Li, Shilei Wang, Alexander Lai, Shuo Su

**Affiliations:** 1 Ministry of Education Joint International Research Laboratory of Animal Health and Food Safety, Engineering Laboratory of Animal Immunity of Jiangsu Province, College of Veterinary Medicine, Nanjing Agricultural University, Nanjing, China; 2 Key Laboratory of Etiology and Epidemiology of Emerging Infectious Diseases in Universities of Shandong, Taishan Medical College, Taian, 271000 China; 3 College of Natural, Applied, and Health Sciences, Kentucky State University, Frankfort, KY USA

## Abstract

Prior to the emergence of H3N8 canine influenza virus (CIV) and the latest avian-origin H3N2 CIV, there was no evidence of a circulating canine-specific influenza virus. Molecular and epidemiological evidence suggest that H3N8 CIV emerged from H3N8 equine influenza virus (EIV). This host-range shift of EIV from equine to canine hosts and its subsequent establishment as an enzootic CIV is unique because this host-range shift was from one mammalian host to another. To further understand this host-range shift, we conducted a comprehensive phylodynamic analysis using all the available whole-genome sequences of H3N8 CIV. We found that (1) the emergence of H3N8 CIV from H3N8 EIV occurred in approximately 2002; (2) this interspecies transmission was by a reassortant virus of the circulating Florida-1 clade H3N8 EIV; (3) once in the canine species, H3N8 CIV spread efficiently and remained an enzootic virus; (4) H3N8 CIV evolved and diverged into multiple clades or sublineages, with intra and inter-lineage reassortment. Our results provide a framework to understand the molecular basis of host-range shifts of influenza viruses and that dogs are potential “mixing vessels” for the establishment of novel influenza viruses.

## Introduction

Influenza A virus (IAV) is a member of *Orthomyxoviridae*. The viral genome consists of eight negative-sensed RNA gene segments. According to the antigenicity of the two viral surface proteins, haemagglutinin (HA) and neuraminidase (NA), IAV is currently classified into 17 HA and 9 NA subtypes. All combinations of the 17 HA and 9 NA subtypes have been found in waterfowl, its natural host [1]. However, only a few defined combinations of HA and NA subtypes circulate in mammalian hosts, e.g., H3N2 and H1N1 among humans and pigs and H3N8 among horses. The establishment of these mammalian influenza viruses, including human influenza A virus (hIAV), was the result of interspecies transmission of an avian influenza virus (AIV) in the past, either directly or through reassortment with circulating contemporary virus. Current evidence supports that interspecies transmission of influenza viruses occurs frequently; however, these occurrences usually result in “dead-end transmission”, possibly due to lack of viral adaptation to the new host species. The roles of the HA, NP, NS, PB1 and PB2 genes in adaption to mammalian hosts have been implicated [2–9]. However, these studies mostly focused on the interspecies transmission of AIV to humans or experimentally inoculated mouse models.

Of the two subtypes of equine influenza virus (EIV), only H3N8 EIV (formerly equine-2 influenza virus) remains circulating in horses, causing sporadic epizootics and disruptions of equine events [10–12]. H3N8 EIV was first identified in Florida in 1963 [13]. Since then, it has spread across multiple continents, including Eurasia [14–16], South America [17, 18], and Australia [19, 20]. Of note, Australia became EIV-free again by aggressive quarantine measures [21, 22]. From its emergence in 1963 to approximately 1990, H3N8 EIV evolved as a monophyletic lineage; since then, it had diverged into American and Eurasian lineages [23], and subsequently, the American lineage further diverged into multiple clades, including Florida-1 [24, 25], of which H3N8 CIV was derived.

Prior to 2003, there was no evidence of circulation of a canine-specific influenza virus despite close contact with humans and with other mammalian species, such as swine and equines. Most influenza viruses can be cultivated in Madin-Darby Canine Kidney (MDCK) cells, indicating the potential susceptibility of dogs to influenza virus infection, at least at the cellular level. In fact, there were reports of isolation of H3N2 human influenza A virus (hIAV) from dogs during influenza epidemics [26], as well as serological evidence of hIAV infections in dogs [27]. Natural and experimental transmission of pandemic H1N1/2009 influenza virus to dogs has been reported [28]. Notably, there were reports of H3N8 EIV infection in a British foxhound [29] as well as in dogs in the United States [30]. These reports suggest that dogs can be a host for influenza A virus. However, these influenza infections were “dead-end” infections, as there was no further sustained spread among dogs.

The emergence of H3N8 CIV in Florida during 2003 [31, 32] was unique. The high genetic homology of the emerging H3N8 CIV strain to the circulating H3N8 EIV of the Florida-1 clade and the geographic coincidence indicated that H3N8 CIV was likely a result of a host-range shift event. Interestingly, H3N8 EIV had been isolated from pigs [33], indicating the susceptibility of pigs to this virus. Interestingly, there was no seroconversion after the experimental inoculation of pigs with H3N8 EIV [34]. There is an apparent species-barrier for H3N8 EIV in pigs, although pigs have been implicated as a “mixing vessel” for influenza viruses [1, 35, 36]. Furthermore, there were no phenotypic differences between H3N8 CIV and H3N8 EIV despite their diverged evolution [37]. Currently, H3N8 CIV is expanding in its geographic distribution and canine host breeds. In addition, CIV has evolved and diverged into multiple lineages and clades.

A significant knowledge gap exists on how and why H3N8 EIV was able to cross the species barrier from equine to canine to become an enzootic CIV. In this study, we performed an extensive and in-depth phylodynamic analysis to address these questions.

## Materials and methods

### Sequence information

#### CIV and EIV whole genome dataset compilation

We conducted an exhaustive search for H3N8 CIV complete genome sequences from the Global Initiative on Sharing All Influenza Data (GISAID) database to generate a working dataset (Additional file 1). A total of 119 HA, 206 M1, 89 NA, 73 NP, 203 NS, 54 PA, 56 PB1, 51 PB2 gene sequences were complied. Likewise, for H3N8 EIV, a total of 349 HA, 212 M1, 331 NA, 170 NP, 233 NS, 153 PA, 158 PB1, and 157 PB2 gene sequences were complied. Eight datasets were created by combining each of the eight gene segments from H3N8 EIV and CIV for subsequent phylogenetic analysis. Phylogenetic analysis of the eight complete datasets was performed using the maximum likelihood (ML) method, followed by a second phylogenetic analysis using a refined subset of gene sequences comprising all H3N8 CIVs and their closely related EIV lineages using RAxML [38]. This refined subset was then analysed to identify the origin of H3N8 CIV.

#### H3N8 CIV datasets for evolutionary adaptation analysis

After excluding partial sequences and non-full-length genome data, a total of forty-four (*n* = 44) complete genomes of H3N8 CIVs were included. The length of each gene segment was as follows: HA: 1695 nucleotides (nt); MP: 756 nt; NA: 1407 nt; NP: 1494 nt; NS: 690 nt; PA: 2148 nt; PB1: 2271 nt; and PB2: 2277 nt. For simplicity, only M1 and NS1 were included for analysis despite their alternative internal RNA splice sites. In all, a total of 12,738 nt for the eight gene segments were analysed.

### Alignment and model selection

Multiple sequence alignments (MSAs) were generated using MUSCLE (version 3.8.31) [39], followed by manual editing using MEGA (version 7.0) [40]. The best-fit model of nucleotide substitution for each sequence dataset was selected using jModelTest [41] according to the Bayesian information criterion (BIC) score. TempEst (version 1.5.1) was used to analyse the temporal signal in the refined dataset sequences.

### Phylogenetic and evolutionary dynamics analyses

All of the maximum-likelihood (ML) trees were constructed by RAxML (version 8.2.4) using the general time reversible (GTR) model and a gamma distribution (G) to account for the rate heterogeneity among sites, and 1000 bootstrap replications were performed [42]. The divergence time between EIV and CIV was estimated using the Bayesian MCMC method implemented in the BEAST (version 1.8.4) package. The GTR or Hasegawa-Kishino-Yano (HKY) model plus gamma distributed rate heterogeneity were used in the nucleotide substitution model. The lognormal relaxed molecular clock was used as the molecular clock model [43], and the Bayesian skyline coalescent model was set as the tree prior. Markov Chain Monte Carlo (MCMC) sampling was run for 10^8^ generations, with trees and posteriors sampled every 10^4^ steps. Each gene dataset was independently run twice and combined using LogCombiner. After a burn-in of 10%, the final maximum clade credibility (MCC) tree was summarized using TreeAnnotator [44] and visualized using Figtree [43]. In addition, the Bayesian inference (BI) tree of each gene segment was built using MrBayes with the HKY + G model, 10^7^ generations and a sampling frequency of 10^3^ [45]. After removing the reassortment segment, the time of most recent common ancestor (tMRCA) and evolutionary rates were estimated using the Bayesian MCMC method.

### Amino acid substitutions and U content analysis

Consensus sequences of different H3N8 CIV clades were aligned, and corresponding mutations (deviations from the consensus) were identified. The mutation position in each clade was confirmed using MESQUITE (a modular, extendible software for evolutionary biology). The number of amino acid changes in each enzootic cluster was counted. The U content of the HA and NA gene segments from multiple influenza A viruses was calculated using BioEdit [46]. Regression and correlation analyses were performed using GraphPad version 7.

### Selection analysis

**Figure 1:**
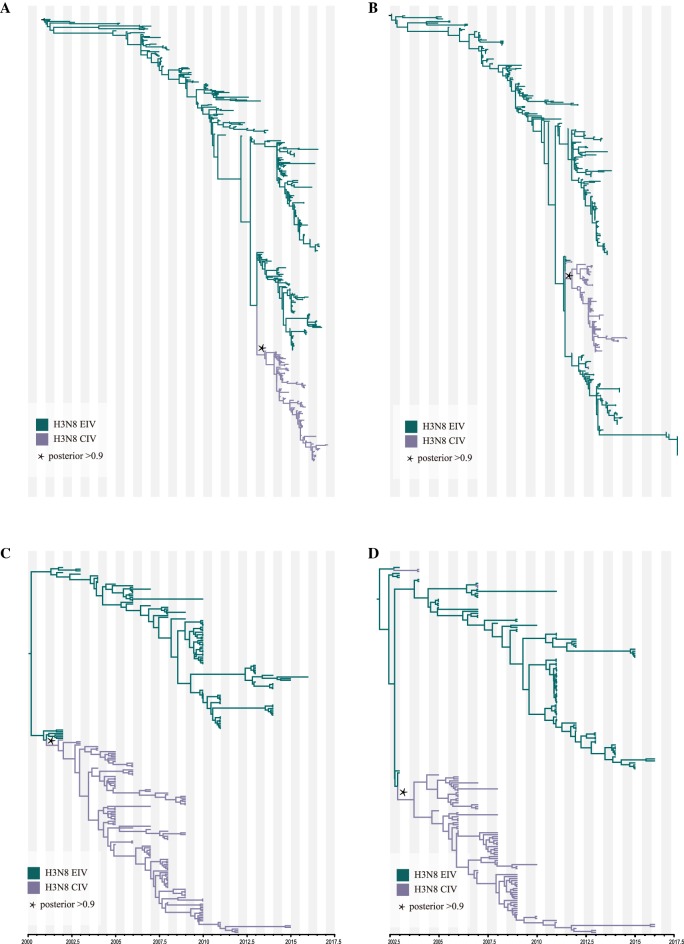
**ML and MCC tree of the HA (A) and NA (B) segments.** The ML trees were constructed using the GTR + gamma nucleotide substitution model with 1000 bootstrap replications (**A**: HA; **B**: NA). The MCC trees were reconstructed using the HKY + gamma nucleotide substitution model and lognormal relaxed clock. The skyline coalescent model length chain was set at 1 × 10^8^ in length and resampled every 1 × 10^4^ steps (**C**: HA; **D**: NA).

**Figure 2:**
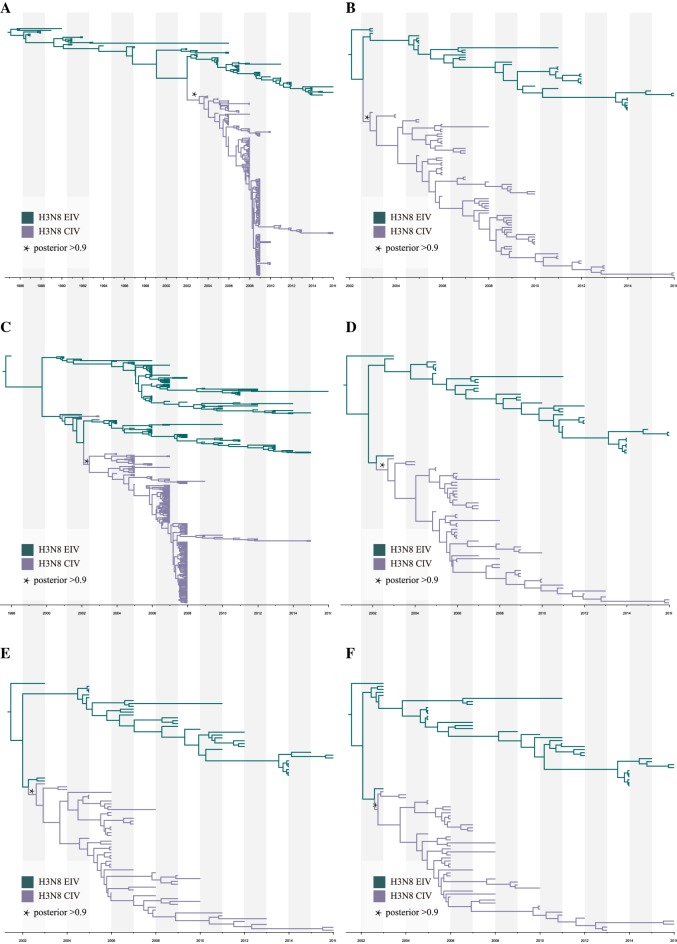
**MCC trees of H3N8 CIV other gene segments.** The MCC trees were reconstructed based on refined subsets of gene sequences from all CIV and closely related EIV sequences using the HKY + gamma nucleotide substitution model and lognormal relaxed clock. The skyline coalescent model length chain was set at 1 × 10^8^ in length and re-sampled every 1 × 10^4^ steps (**A**: M1; **B**: NP; **C**: NS1; **D**: PA; **E**: PB1; **F**: PB2).

The MCC trees (Figures 1C, D, 2 and 5) were used as the input reference trees in DATAMONKEY [47], which was used to estimate selection pressures. The SLAC (Single Likelihood Ancestry Counting), FEL (Fixed Impact Probability), MEME (Evolutionary Mixed Effects Model) and FUBAR (Fast, Unconstrained Bayesian Approximation) methods were used to identify codons under positive selection. The branch-site REL model was used to determine the selection pressure along the branches [48–50]. We considered *p* values of SLAC, FEL and MEME less than 0.1 and a FUBAR posterior probability > 0.9 as significant, and only sites supported by at least three methods were reported. To reduce the bias due to reassortment, a further refined dataset was produced by removing suspected sequences. The mean ratio of non-synonymous substitution per site (dN/dS) was then calculated using the SLAC method.

## Results

### Origin of H3N8 CIV

**Figure 3:**
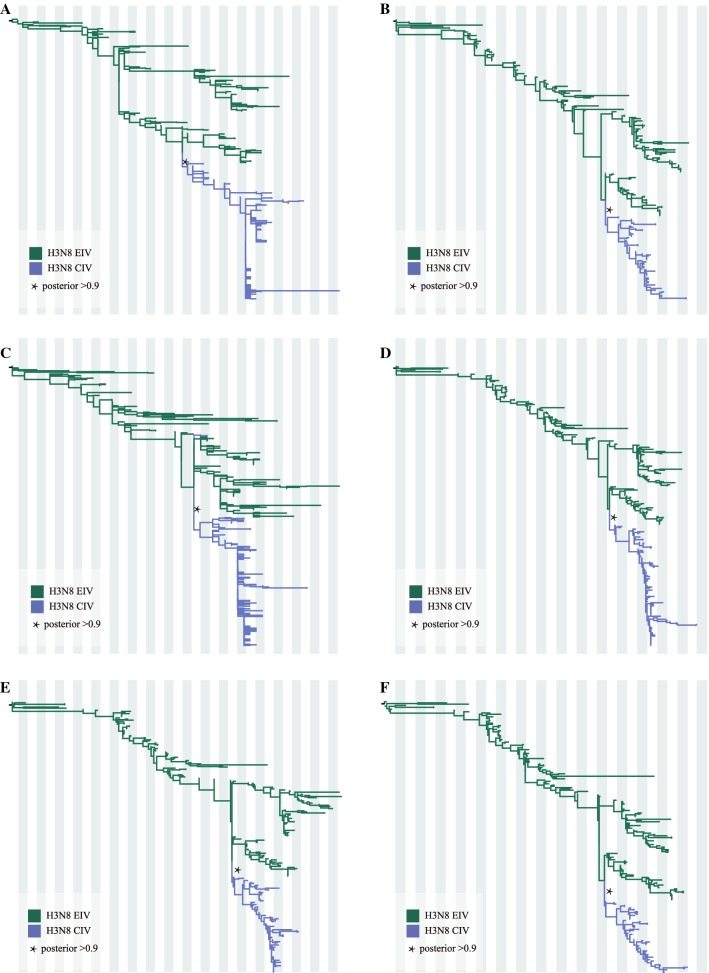
**Original ML trees of the internals genes of H3N8 CIV.** ML trees were constructed using the GTR + gamma nucleotide substitution model with 1000 bootstrap replications. **A**: M1; **B**: NP; **C**: NS; **D**: PA; **E**: PB1; **F**: PB2.

In agreement with published reports, both temporal-spatial and molecular evidence suggest that H3N8 CIV originated from H3N8 EIV. Figure 1 shows the phylogenies of the gene segments for the surface proteins, HA and NA; Figures 1A, B show the initial ML trees, and Figures 1C, D were generated by selecting subtrees that included all H3N8 CIVs and related lineages to identify the origin. Similarly, the phylogenies of the other six internal genes (M1, NP, NS1, PA, PB1, PB2), as shown in Figure 2, further support the origin of CIV from EIV. In addition, the original ML trees also indicated that each gene segment of H3N8 CIV was closely related to the H3N8 EIV lineage (Figure 3), except for A/Florida/242/2003, A/Florida/15592.1/2004, and A/Florida/43/2004, which were not clustered with the other CIV segments in the NS1 gene tree (Figure 3C). These isolates were among the “emerging clade”. Therefore, the emerging H3N8 CIV appeared to originate from a reassortant H3N8 EIV. Further evidence is provided below.

### Phylogenetic and evolution dynamics of H3N8 CIV

**Figure 4:**
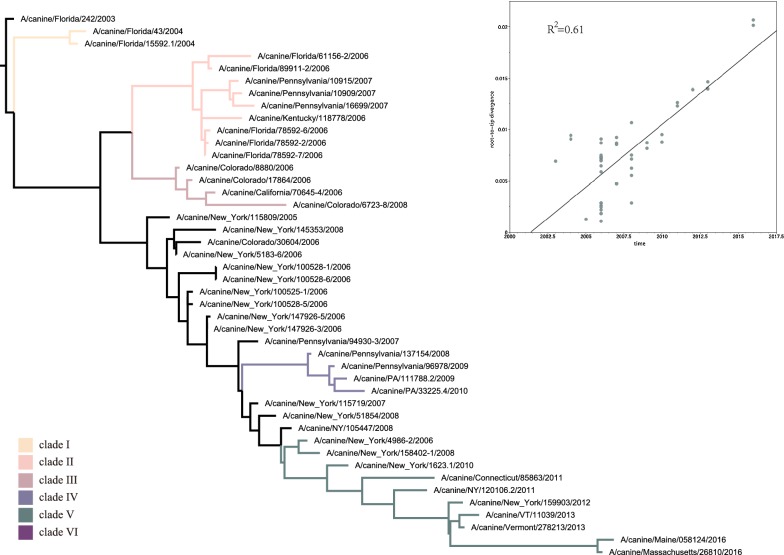
**ML tree of the H3N8 CIV concatenated full genome.** From the dataset of the 44 full viral genome sequences, after splicing for segments M and NS, the segments were concatenated (HA, M1, NA, NP, NS1, PA, PB1, and PB2) for each virus, followed by the generation of a maximum-likelihood (ML) tree. The tree was constructed using the HKY + gamma nucleotide substitution model with 1000 bootstrap replications. Insert: Regression analysis of branch-length to year of virus isolation. Colour rectangles indicate different clades: clade I (yellow), clade II (pink), clade III (brown), clade IV (blue), clade V (green), clade VI (purple).

**Figure 5:**
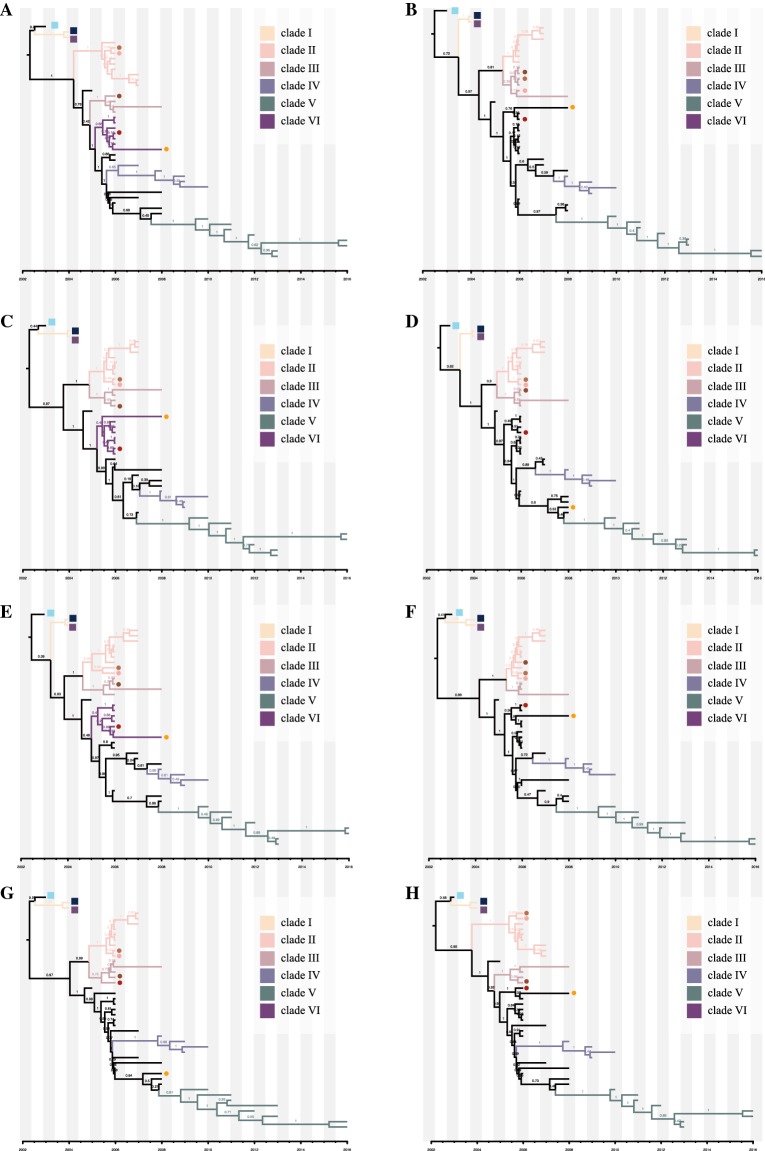
**MCC tree for each H3N8 CIV segment.** MCC trees were constructed using the BEAST (V1.8.4) program with a GTR plus gamma nucleotide substitution mode and relaxed molecular clock. The prior tree set as the coalescent: Bayesian skyline with 10^8^ generations (**A**: HA; **B**: M1; **C**: NA; **D**: NP; **E**: NS1; **F**: PA; **G**: PB1; **H**: PB2). The different coloured rectangles indicate different clades: clade I (yellow), clade II (pink), clade III (brown), clade IV (blue), clade V (green), and clade VI (purple). Squares and circles indicate viruses that underwent intrasubtypic reassortment and further reassortment, respectively. Light-blue square: A/Florida/242/2003; Dark-blue square: A/Florida/43/2004; Purple square: A/Florida/15592.1/2004; Light-tan circle: A/Florida/89911-2/2006; Coral circle: A/canine/Florida/61156-5/2006; Tan circle: A/Colorado/8880/2006; Scarlet circle: A/Colorado/30604/2006; Orange Circle: A/canine/New York/145353/2008.

From the dataset of the 44 full viral genome sequences, after splicing for segments M and NS, the segments were concatenated (HA, M1, NA, NP, NS1, PA, PB1, and PB2) for each virus, followed by generation of a maximum-likelihood (ML) tree (Figure 4). According to the topology of this concatenated ML tree, H3N8 CIV could be divided into six major clades. Furthermore, a regression analysis using the root-to-tip distance of the ML tree of the full-length genome (Figure 4 insert) showed that the R^2^ was 0.61, indicating a somewhat linear relationship between nucleotide divergence and time, hence satisfying the criterion for Bayesian analysis. The MCC trees for each of the eight gene segments were subsequently generated, as shown in Figure 5. As shown in Figures 4 and 5, Clade I consists of early viruses isolated from Florida in 2004. Clade II consists of isolates exclusively from Florida and Pennsylvania from 2006 to 2007. Clade III consists of isolates from Colorado and California from 2006 to 2008. Clade IV consists of isolates from Pennsylvania from 2008 to 2010. Clade V consists of strains circulating in Eastern States between 2010 and 2016. Clade VI consists of viruses from New York in 2006.

**Figure 6:**
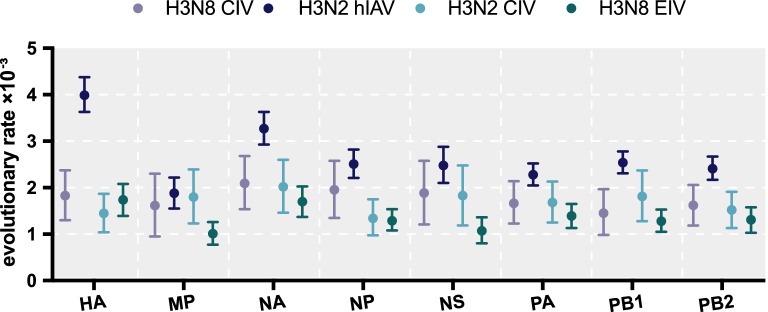
**Evolutionary rates of canine, equine, and human IAVs.** Evolutionary rates of each segment of H3N8 CIV, H3N8 EIV, H3N2 CIV, and H3N2hIAV. The evolutionary rates of each segment were simulated using the BEAST (V1.8.4) program with a GTR plus gamma nucleotide substitution mode, relaxed molecular clock, and a prior coalescent Bayesian skyline tree with 10^8^ generations.

A Bayesian MCMC method was then utilized to estimate the evolutionary rate for each gene segment (Figure 6). Despite the small sample size, the MCC trees of each gene segment exhibited similar topology and maintain five stable clades for M1, NP, PA, PB1, and PB2. However, an additional clade VI exists for HA, NA, and NS. Furthermore, as shown in Figure 5, intrasubtypic reassortment was detected among these six clades. For example, for A/canine/Florida/61156-5/2006 and A/Florida/89911-2/2006, while the M1 gene segment clusters within clade III, their other gene segments cluster within clade II; for A/Colorado/8880/2006, the PA gene segment clusters in clade II, while its other segments cluster within clade III; and for A/Colorado/30604/2006, the PB1 gene segment clusters in clade III, whereas the other gene segments cluster in clade VI. The topology of each BI tree of the eight gene segments was similar to that of the MCC tree (data not shown).

To achieve accurate dating of each gene segment, reassortant sequences were first removed from the dataset. The time of the most recent common ancestor (tMRCA) and the time of divergence were computed as described in the Materials and methods. As shown in Table 1, the tMRCA of HA, NA, PA and PB2 was 2002 (95% highest posterior density interval (HPD): 2000–2003), of M1 was 2002 (95% HPD 1998–2003), of PB1 was 2002 (95% HPD 1999–2003), of NP was 2003 (95% HPD 2000–2003) and of NS was 2003 (95% HPD 1997–2005). In general, these tMRCAs were in agreement with the time scale for the documented emergence of H3N8 CIV, within the timeframe of approximately 2002. However, for the time of divergence, NS1 appears to have diverged earlier than the rest—in 2001—with an estimated interval from 1999 to 2002. In addition, the range of tMRCA for NS1 was broader than the rest, from 1997 to 2005. To validate this estimated time of divergence for NS1, a sample of published NS (nucleotide) sequences for Florida-1 EIV and CIV were used in a regression analysis. A plot of the divergence of nucleotides over time (dN/t) showed that they intercepted around 2001–2002 (Additional file 2), supporting the tMRCA analysis. Furthermore, a crucial role of NS1 was revealed by a comparison of key amino acid residues. The NS sequence of A/equine/Ohio/1/2003, a Florida-1 clade EIV, was used as a reference. A/canine/Florida/242/2003 was used as the emerging CIV, and isolates from the other six clades (I to VI) are shown in Table 2. There were variations among the six identified CIV clades (I to VI). Some were unique for specific clades, e.g., L50I was unique for clade II. Some were “fixed” for later clades, e.g., V156I and L185F for clades IV to VI. Some had more than one variation, e.g., P212H for clade II and P212S for clade V. Some of these unique amino acid residues were within identified functional domains, e.g., a nuclear transport signal (137–146): 140G and 140R for clade I and clade II to VI, respectively [51].

**Table 1:** **The tMRCA and divergence time for each segment of H3N8 CIV**

Segment	tMRCA		Divergence time	
	Mean	95% HPD interval	Mean	95% HPD interval
HA	2002	[2000–2003]	2002	[2000–2002]
M1	2002	[1998–2003]	2002	[2001–2003]
NA	2002	[2000–2003]	2003	[2002–2003]
NP	2003	[2000–2003]	2003	[2002–2003]
NS1	2003	[1997–2005]	2001	[1999–2002]
PA	2002	[2000–2003]	2002	[2001–2003]
PB1	2002	[1999–2003]	2002	[2001–2003]
PB2	2002	[2000–2003]	2003	[2002–2003]

**Table 2:** **Variations in amino acid sequences in the NS1 genes**

Position	Amino acid residues in the NS1 gene											
	21	50	72	77	86	88	140	156	185	193	212	214
EIV/Ohio/2003	R	L	E	L	T	R	G	V	L	R	P	F
CIV/Florida/2003	R	L	E	L	T	R	G	V	L	R	P	F
CIV-Clade I	R	L	E	L	T	L	G	V	L	R	P	F
CIV-Clade II	R	I	E	L	A	R	R	V	L	R	H	F
CIV-Clade III	R	L	E	L	A	R	R	V	L	R	P	F
CIV-Clade IV	Q	L	E	P	A	R	R	I	F	R	P	F
CIV-Clade V	Q	L	E	P	A	R	R	I	F	K	S	L
CIV-Clade VI	R	L	K	P	A	R	R	I	F	R	P	F

Taken together, the emergence of CIV from EIV was not from a whole EIV progenitor virus per se but by a “reassortant” virus involving a unique NS gene segment.

We also calculated the evolutionary rates for different H3N8 CIV gene segments. Overall, after removing the reassortant sequences of each segment, the eight gene segments evolved at similar rates over the 13 years since the first report in dogs. The evolutionary rate of HA was 1.83 × 10^−3^ (95% HPD 1.3–2.37 × 10^−3^ substitutions/site/year) and that of NA was 2.09 × 10^−3^ (95% HPD 1.54–2.68 × 10^−3^ substitutions/site/year). For the other gene segments, the evolutionary rates ranged from 0.85 to 2.58 × 10^−3^ substitution evolutionary rates/site/year (Figure 6). In general, the evolutionary rate for each gene segment was higher in H3N8 CIV than in H3N8 EIV [52]. However, these rates were at the low end of the 95% HPD when compared to that of H3N2 hIAV [53].

**Figure 7:**
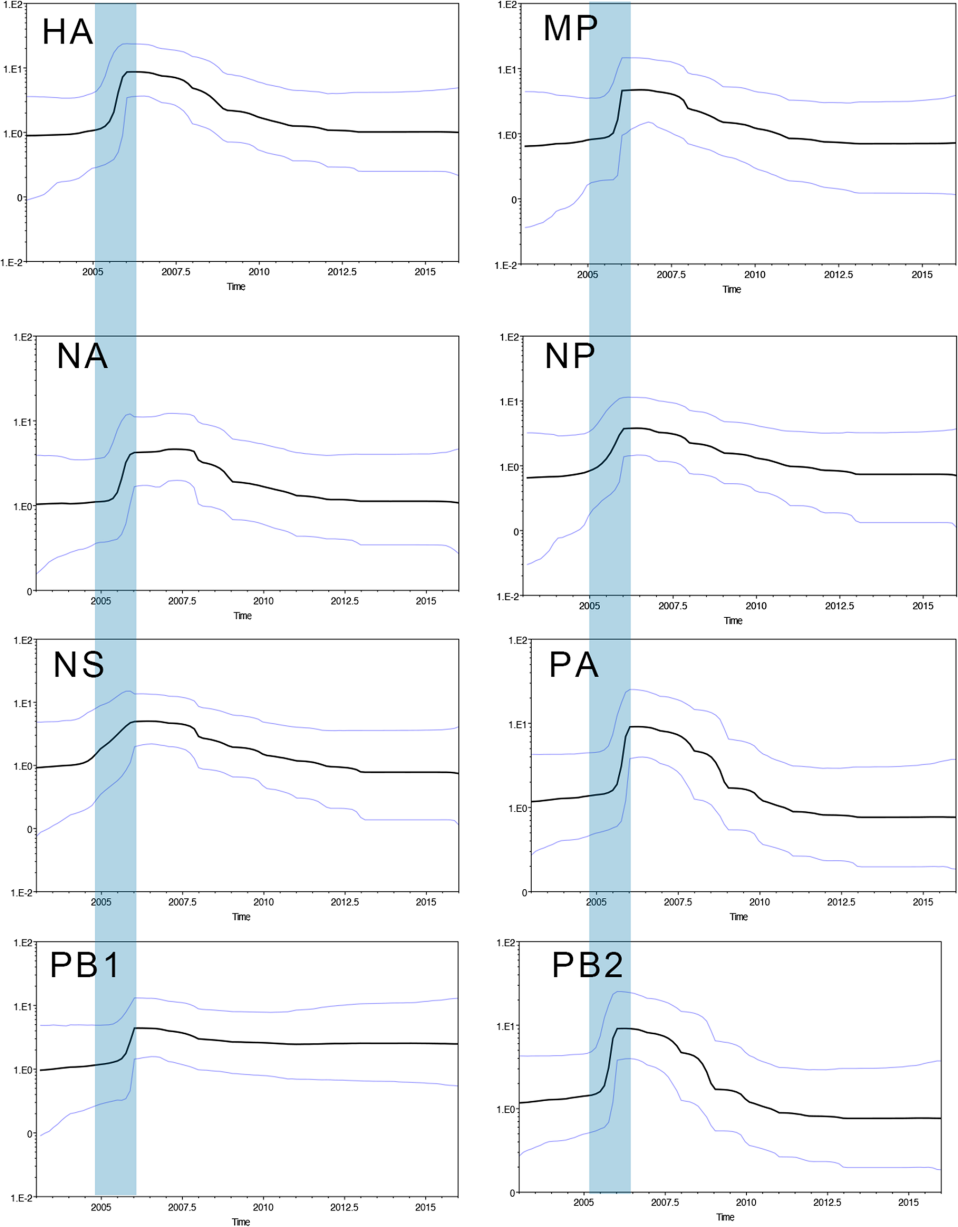
**The Bayesian Skyline plot (BSP) of H3N8 CIV in each segment.** BSPs were simulated using the BEAST (V1.8.4) program, based on all eight segments of 44 strains, with a GTR plus gamma nucleotide substitution mode and relaxed molecular clock. The prior tree set as the coalescent: Bayesian skyline with 10^8^ generations. The blue vertical bar indicates the period of divergence of major H3N8 lineages in canine species.

To reconstruct the population dynamics for the emergence of H3N8 CIV, a skyline plot was constructed. As shown in Figure 7, the effective population size of H3N8 CIV increased sharply between 2005 and 2006 for almost all the gene segments, with the exception of NS1. In addition, this sharp increase coincided with the time of divergence of H3N8 CIV into multiple clades (I to V).

### Adaptation of H3N8 CIV in canine species

To investigate the molecular changes that occurred in H3N8 CIV after interspecies transmission to the new host species, changes in the amino acid residues for the eight viral proteins, including HA, M1, NA, NP, NS, PA, PB1 and PB2, were analysed. Variations in the amino acid sequences are tabulated in Additional file 3. Regarding the HA gene of CIV and EIV (the positioning or numbering of amino acid residues in the HA gene is based on the starting codon for H3 HA), as previously described by Wen et al. [54], there was a W222L substitution at the receptor-binding site in all CIV HA genes. However, for the antigenic sites A to D [25], there was a N54K substitution at antigenic site C in the CIV HA gene. Several non-synonymous substitutions in the HA gene were noted: V41I for clade V; G479D for clades II, III, and IV; and G479N for clade V. However, it reverted back to 479G for clade VI. Whether this N495G was a true reversion or a result of reassortment remains to be determined. Additional variations for other viral proteins are shown in Additional file 3. The phenotypic significance of these variations, however, remains to be determined.

**Figure 8:**
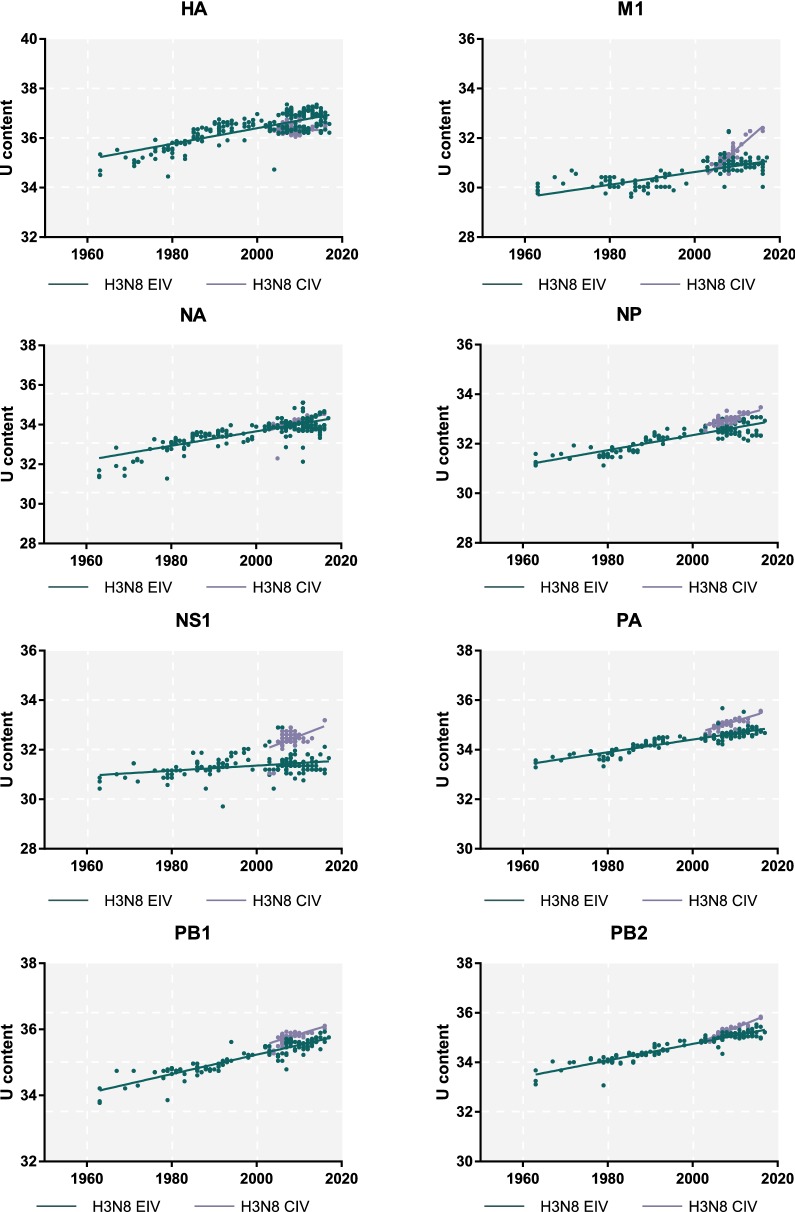
**The U content of H3N8 CIV.** U content was calculated using BioEdit, and the regression curves over time for each segment of H3N8 CIV (blue line) and H3N8 EIV (red line) were performed using GraphPad version 7.

In addition, we also analysed the Uracil (U) content of each segment in both H3N8 CIV and H3N8 EIV to assess the degree of adaptation to the corresponding host species [55, 56]. For H3N8 EIV, the time frame spans from 1963 to present, whereas for H3N8 CIV, it spans from 2002 onwards. As shown in Figure 8, there was an increasing trend in the U content over time (*p* < 0.0001) for all gene segments in both viruses, with the exception of the HA segment of H3N8 CIV, in which the U content gradually decreased over time. However, this difference was not statistically significant. Therefore, the HA gene of this emerging H3N8 CIV, in contrast to other gene segments, appeared to be “adaptation stagnant”. For NA, NP, PA, PB1 and PB2, the increasing rate of the U content for H3N8 CIV was similar to that of H3N8 EIV. Furthermore, for M1 and NS1, the rate of increase in CIV was significantly greater than that in EIV, suggesting that these two genes were under selective pressure to adapt in the new canine host.

**Figure 9:**
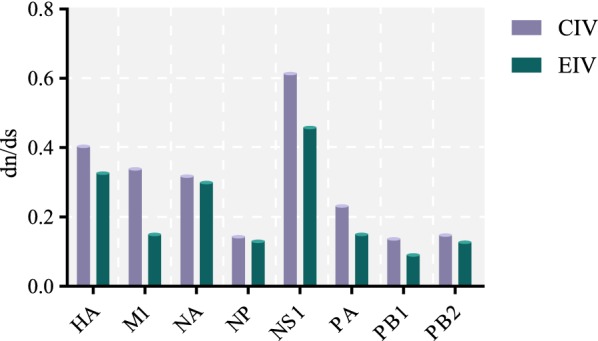
**Mean dN/dS of H3N8 CIV and EIV in each segment.** dN/dS were estimated using the SLAC algorithm on the website DATAMONKEY, and the H3N8 CIVs are shown by blue bars. The H3N8 EIVs are shown by red bars.

Finally, the mean ratio dN/dS was calculated using SLAC, as shown in Figure 9. Interestingly, the mean dN/dS values for all gene segments of H3N8 CIV were higher than the corresponding gene segments of H3N8 EIV, indicating that CIV accumulated more non-synonymous substitutions after interspecies transmission to canines. The mean dN/dS values of the major coding regions of CIV ranged from 0.14 to 0.40, except for NS1, for which the dN/dS ratio was 0.6. In particular, there was a significant difference in the M1 gene, as the dN/dS ratio of CIV was almost double that of EIV.

## Discussion

### Time of emergence

Overall, our results indicate that the divergence time of H3N8 CIV was between 2002 and 2003. Interestingly, serological evidence of H3N8 EIV infection in British foxhounds was also determined to be approximately 2002 [29]. Furthermore, the H3N8 EIV infecting British foxhounds was closely related to representatives of a sub-lineage of American viruses; this was a first incursion of this lineage into the region [57]. Without viral isolation, it is difficult to determine if the virus infecting the British foxhounds was identical to that from Florida. Why did these two events of interspecies transmission by an apparently similar contemporary EIV result in different clinical outcomes? Our results indicated that the emerging H3N8 CIV was a reassortant virus that had fully adapted to the canine host when it emerged in 2002. However, whether there were multiple “attempts” by H3N8 EIV to establish in a canine host or whether there were “back-n-forth” progenitor viruses between the two host species, of which one successfully established, remains a major knowledge gap. That “successful” progenitor virus appeared to be a reassortant virus, as discussed below.

### The emerging strain was a reassortant virus

From the topology of the ML and MCC trees, we concluded that the emerged H3N8 CIV in Florida was a reassortant virus with an NS1 gene segment from an earlier EIV, which was also supported by the tMRCA of the NS1 gene segment to have emerged 1997–2005. The NS1 gene of influenza viruses, including H3N8 EIV, has been shown to counteract host innate immunity [51, 58, 59]. Furthermore, NS1 of EIV was categorized into a pre-divergent lineage, Eurasian and American/Kentucky sub-lineages, and subsequently into Florida-1 and Florida-2 clades [60]. Due to the segmented genome, the viral phenotype depends on the combination of the gene segments or its “gene constellation” [61]. It has also been shown that multiple EIVs have infected canines, and reassortment events have occurred in canines. The host-switch of EIV to CIV by the Florida-1 clade virus was “successful”, while the interspecies transmission event in England was not. Furthermore, during the spread of H3N8 EIV in Australia in 2007, dogs in close contact with infected horses developed clinical symptoms, with virus isolation from two index cases, but the virus did not spread further [62]. One possibility is that in A/equine/Sydney/2007 (or “would be” A/canine/Sydney/2007), unlike A/canine/Florida/2003, the H3N8 EIV that crossed species to dogs in Sydney lacked the NS1 gene segment from a putative earlier H3N8 EIV and hence became a “dead-end” event, as shown by the phylogeny of the HA1 gene segment [63]. However, other factors, such as improved quarantine and a history of “back and forth” virus transmission, cannot be ruled out. Notably, the “donor” of the NS1 gene segment to the emerging strain of H3N8 CIV is particularly interesting and remains to be elucidated. It suggests that possibly its “evolutionary clock” differs from that of other gene segments, but this is unlikely. An alternative hypothesis is that there was a “frozen” virus strain in circulation. However, taking together, this “successful” interspecies transmission was a “rarity”, as a unique combination of gene segments was required, plus a unique amino acid substitution at the receptor-binding site in the HA gene (W222L) [54]. Therefore, this interspecies transmission was not a “wholesale” event, but rather occurred during at least two events, one involving reassortment of a unique combination of gene segments and a mutation or adaptation at the receptor-binding site in the HA. However, whether these events were simultaneous or sequential remains unknown.

### Adaptations to canine hosts

According to the tMRCA for each gene segment with regard to the time of virus emergence, the “adaptation” had occurred while in the equine host. This “pre-emergence adaptation” could be a characteristic of EIV in the Florida-1 clade, which might not occur in other sublineages. Phenotypic heterogeneity is common in equine influenza virus. For example, there is heterogeneity in virulence among different strains of EIV in the early American lineage (unpublished results). As discussed above, although the emerging strain was a reassortant strain and the reassortment event likely occurred in the equine host, back-and-forth interspecies transmission between the canine and equine host prior to the emergence of CIV cannot be ruled out.

In addition, the U content of each gene segment was analysed for both H3N8 CIV and H3N8 EIV. An increasing U content in viral gene segments is an indicator of viral adaptation to a new host species [64]. There was an increasing trend in the U content of each gene segment over time for both H3N8 CIV and H3N8 EIV (Figure 8), indicating that both viruses were adapting to their respective hosts. However, the rate of increase was significantly higher in the NS1 and M1 genes from H3N8 CIV. As discussed above, NS1 is involved in counteracting the host innate immune response, specifically downregulating interferon expression and interferon-induced antiviral responses [65]. Our results indicate that the adaptation of H3N8 CIV to canine species involved overcoming host innate immunity both at the pre-emerging and post-enzootic stages. Elucidating the underlying mechanism of this differential rate of increase in the U content between CIV and EIV for NS1 and M1 may shed light on this host-range shift from one mammalian host to another.

Changes in critical amino acid residues may affect the functionality of viral proteins, and the changes may be a result of selection or adaptation. Variations, particularly among different clades, as shown in Additional file 3, in addition to known functions such as nuclear transport signalling or receptor-binding, can only be delineated by experimental approaches. These experiments are ongoing.

### Diverged evolution and intrasubtypic reassortment

Our phylogenetic analyses revealed that enzootic H3N8 CIV has evolved into five to six clades (I to VI). This rapid divergence is in contrast to the parental H3N8 EIV, which only diverged in approximately 1990 after a long-term monophyletic evolution since it was first identified in 1963. Whether it was due to a “founder effect” by geographic restriction or a function of selective pressure remains to be determined.

Reassortment among multiple lineages of co-circulating influenza viruses is not uncommon [66–68]. Likewise, for the diverged sublineages of H3N8 CIV, different gene segment clustering in different clades was observed in several viruses, indicating intrasubtypic reassortment. Murcia et al. [52] showed that intrasubtypic reassortment is common in H3N8 EIV, and reassortment results in enhanced virulence. Whether these CIV reassortments among these multiple clades have biological significance remains to be determined.

The overall evolutionary rate of H3N8 CIV was lower than that of H3N2 hIAV, higher than that of H3N8 EIV, and similar to that of H3N2 CIV [69]. In addition, the evolutionary rate of each gene segment of H3N8 CIV was similar to each other. In addition, from the skyline plot, the effective population size of H3N8 CIV increased between 2005 and 2006, consistent with the genetic divergence during this period.

Interestingly, current epidemiological data indicate that H3N2 CIV mainly affects pet dogs, while H3N8 CIV affects stray dogs and dogs in shelters. Given the recent report of the susceptibility of dogs to a wide spectrum of influenza viruses, the high frequency of reassortment among these influenza viruses [70] and that stray dogs have a high risk of contact with other species, including birds and humans, the risk of dogs as a “mixing vessel” generating a pandemic influenza virus should not be overlooked.

In conclusion, by conducting a phylodynamic analysis to reconstruct the evolutionary path of CIV, we have not only demonstrated that the interspecies transmission of H3N8 EIV to canine occurred in approximately 2002 but also shown that the progenitor virus was a reassortant virus involving an “ancestral NS1 gene segment”. Interspecies transmission of influenza viruses might occur more frequently than previously thought, but to adapt and remain in a new host species, that is, to undergo a host-range shift, unique combinations of gene segments plus critical mutations are required. Furthermore, H3N8 CIV has diverged into multiple sublineages or clades, possibly as a result of founder effect, in which the founder virus evolves within geographic or facility boundaries. Similar to other influenza viruses, frequent reassortment within and among these clades was observed. These findings provide a conceptual framework to understand the mechanism of interspecies transmission and host-range shifts of influenza viruses. Finally, although we utilized a computation-intensive genomics approach to reconstruct this host-range shift and the subsequent evolution and adaptation of CIV, phenotypic studies are still required to validate these results. These experiments are ongoing.

## Supplementary information

**Additional file 1.** **List of genetic sequences from multiple influenza A viruses used in this study**.

**Additional file 2. Regression analysis of the rate of nucleotide substitutions for the NS1 gene of EIV and CIV**.

**Additional file 3.** **Amino acid changes that differentiate H3N8 CIV into six clades**.

## Data Availability

All authors hereby state that the data will be made available according to the general policy of *Veterinary Research*.

## Abbreviations

EIV: equine influenza virus
CIV: canine influenza virus
AIV: avian influenza virus
hIAV: human influenza A virus
HA: haemagglutinin
NA: neuraminidase
NP: nucleoprotein
NS: non-structural protein
PB1: basic protein 1
M: matrix protein
nt: nucleotide
GISAID: Global Initiative on Sharing All Influenza Data
ML: maximum likelihood
RAxML: randomized accelerated maximum likelihood
MSA: multiple sequence alignments
MEGA: molecular evolutionary genetics analysis
MUSCLE: multiple sequence comparison by log-expectation
GTR: general time reversible
HKY: Hasegawa-Kishino-Yano
MCMC: Markov chain Monte Carlo
MCC: maximum clade credibility
tMRCA: time of most recent common ancestor
SLAC: single likelihood ancestry counting
FEL: fixed impact probability
MEME: evolutionary mixed effects model
FUBAR: fast, unconstrained Bayesian approximation
HPD: highest posterior density interval
U: uracil
BEAST: Bayesian Evolutionary Analysis Sampling Trees

## References

[1] Webster RG, Bean WJ, Gorman OT, Chambers TM, Kawaoka Y (1992). Evolution and ecology of influenza A viruses. Microbiol Rev.

[2] Gorman OT, Bean WJ, Kawaoka Y, Donatelli I, Guo YJ, Webster RG (1991). Evolution of influenza A virus nucleoprotein genes: implications for the origins of H1N1 human and classical swine viruses. J Virol.

[3] Gorman OT, Donis RO, Kawaoka Y, Webster RG (1990). Evolution of influenza A virus PB2 genes: implications for evolution of the ribonucleoprotein complex and origin of human influenza A virus. J Virol.

[4] Kawaoka Y, Gorman OT, Ito T, Wells K, Donis RO, Castrucci MR, Donatelli I, Webster RG (1998). Influence of host species on the evolution of the nonstructural (NS) gene of influenza A viruses. Virus Res.

[5] Bean WJ, Schell M, Katz J, Kawaoka Y, Naeve C, Gorman O, Webster RG (1992). Evolution of the H3 influenza virus hemagglutinin from human and nonhuman hosts. J Virol.

[6] Danzy S, Studdard LR, Manicassamy B, Solorzano A, Marshall N, Garcia-Sastre A, Steel J, Lowen AC (2014). Mutations to PB2 and NP proteins of an avian influenza virus combine to confer efficient growth in primary human respiratory cells. J Virol.

[7] Mok CK, Lee HH, Lestra M, Nicholls JM, Chan MC, Sia SF, Zhu H, Poon LL, Guan Y, Peiris JS (2014). Amino acid substitutions in polymerase basic protein 2 gene contribute to the pathogenicity of the novel A/H7N9 influenza virus in mammalian hosts. J Virol.

[8] Song W, Wang P, Mok BW, Lau SY, Huang X, Wu WL, Zheng M, Wen X, Yang S, Chen Y, Li L, Yuen KY, Chen H (2014). The K526R substitution in viral protein PB2 enhances the effects of E627K on influenza virus replication. Nat Commun.

[9] Kim JH, Hatta M, Watanabe S, Neumann G, Watanabe T, Kawaoka Y (2010). Role of host-specific amino acids in the pathogenicity of avian H5N1 influenza viruses in mice. J Gen Virol.

[10] Daly JM, MacRae S, Newton JR, Wattrang E, Elton DM (2011). Equine influenza: a review of an unpredictable virus. Vet J.

[11] Lai AC, Lin YP, Powell DG, Shortridge KF, Webster RG, Daly J, Chambers TM (1994). Genetic and antigenic analysis of the influenza virus responsible for the 1992 Hong Kong equine influenza epizootic. Virology.

[12] Landolt GA (2014). Equine influenza virus. Vet Clin North Am Equine Pract.

[13] Waddell GH, Teigland MB, Sigel MM (1963). A new influenza virus associated with equine respiratory disease. J Am Vet Med Assoc.

[14] Powell DG, Thomson GR, Spooner P, Plowright W, Burrows R, Schild GC (1974). The outbreak of equine influenza in England April–May 1973. Vet Rec.

[15] Wilson WD (1993). Equine influenza. Vet Clin North Am Equine Pract.

[16] Oxburgh L, Berg M, Klingeborn B, Emmoth E, Linne T (1994). Evolution of H3N8 equine influenza virus from 1963 to 1991. Virus Res.

[17] Olguin Perglione C, Golemba MD, Torres C, Barrandeguy M (2016). Molecular epidemiology and spatio-temporal dynamics of the H3N8 equine influenza virus in South America. Pathogens.

[18] Alves Beuttemmuller E, Woodward A, Rash A, Dos Santos Ferraz LE, Fernandes Alfieri A, Alfieri AA, Elton D (2016). Characterisation of the epidemic strain of H3N8 equine influenza virus responsible for outbreaks in South America in 2012. Virology.

[19] Reeve-Johnson L (2007). Equine influenza in Australia. Vet Rec.

[20] Alder M (ed) (2008) Summary of the Australian equine influenza outbreak. Vet Rec 163:378 10.1136/vr.163.13.378 19031639

[21] Scott-Orr H (2011). Proof of freedom from equine influenza infection in Australia in 2007–08. Aust Vet J.

[22] Watson J, Daniels P, Kirkland P, Carroll A, Jeggo M (2011). The 2007 outbreak of equine influenza in Australia: lessons learned for international trade in horses. Rev Sci Tech.

[23] Daly JM, Lai AC, Binns MM, Chambers TM, Barrandeguy M, Mumford JA (1996). Antigenic and genetic evolution of equine H3N8 influenza A viruses. J Gen Virol.

[24] Lai AC, Rogers KM, Glaser A, Tudor L, Chambers T (2004). Alternate circulation of recent equine-2 influenza viruses (H3N8) from two distinct lineages in the United States. Virus Res.

[25] Lai AC, Chambers TM, Holland RE, Morley PS, Haines DM, Townsend HG, Barrandeguy M (2001). Diverged evolution of recent equine-2 influenza (H3N8) viruses in the Western Hemisphere. Arch Virol.

[26] Chang CP, New AE, Taylor JF, Chiang HS (1976). Influenza virus isolations from dogs during a human epidemic in Taiwan. Int J Zoonoses.

[27] Kilbourne ED, Kehoe JM (1975). Demonstration of antibodies to both hemagglutinin and neuraminidase antigens of H3N2 influenza A virus in domestic dogs. Intervirology.

[28] Lin D, Sun S, Du L, Ma J, Fan L, Pu J, Sun Y, Zhao J, Sun H, Liu J (2012). Natural and experimental infection of dogs with pandemic H1N1/2009 influenza virus. J Gen Virol.

[29] Daly JM, Blunden AS, Macrae S, Miller J, Bowman SJ, Kolodziejek J, Nowotny N, Smith KC (2008). Transmission of equine influenza virus to English foxhounds. Emerg Infect Dis.

[30] Anderson TC, Bromfield CR, Crawford PC, Dodds WJ, Gibbs EP, Hernandez JA (2012). Serological evidence of H3N8 canine influenza-like virus circulation in USA dogs prior to 2004. Vet J.

[31] Crawford PC, Dubovi EJ, Castleman WL, Stephenson I, Gibbs EP, Chen L, Smith C, Hill RC, Ferro P, Pompey J, Bright RA, Medina MJ, Johnson CM, Olsen CW, Cox NJ, Klimov AI, Katz JM, Donis RO (2005). Transmission of equine influenza virus to dogs. Science.

[32] Payungporn S, Crawford PC, Kouo TS, Chen LM, Pompey J, Castleman WL, Dubovi EJ, Katz JM, Donis RO (2008). Influenza A virus (H3N8) in dogs with respiratory disease, Florida. Emerg Infect Dis.

[33] Tu J, Zhou H, Jiang T, Li C, Zhang A, Guo X, Zou W, Chen H, Jin M (2009). Isolation and molecular characterization of equine H3N8 influenza viruses from pigs in China. Arch Virol.

[34] Solorzano A, Foni E, Cordoba L, Baratelli M, Razzuoli E, Bilato D, Martin del Burgo MA, Perlin DS, Martinez J, Martinez-Orellana P, Fraile L, Chiapponi C, Amadori M, del Real G, Montoya M (2015). Cross-species infectivity of H3N8 influenza virus in an experimental infection in swine. J Virol.

[35] Webster RG, Hinshaw VS, Bean WJ, Turner B, Shortridge KF (1977). Influenza viruses from avian and porcine sources and their possible role in the origin of human pandemic strains. Dev Biol Stand.

[36] Shortridge KF, Webster RG, Butterfield WK, Campbell CH (1977). Persistence of Hong Kong influenza virus variants in pigs. Science.

[37] Feng KH, Gonzalez G, Deng L, Yu H, Tse VL, Huang L, Huang K, Wasik BR, Zhou B, Wentworth DE, Holmes EC, Chen X, Varki A, Murcia PR, Parrish CR (2015). Equine and canine influenza H3N8 viruses show minimal biological differences despite phylogenetic divergence. J Virol.

[38] Stamatakis A (2006). RAxML-VI-HPC: maximum likelihood-based phylogenetic analyses with thousands of taxa and mixed models. Bioinformatics.

[39] Edgar RC (2004). MUSCLE: multiple sequence alignment with high accuracy and high throughput. Nucleic Acids Res.

[40] Kumar S, Stecher G, Tamura K (2016). MEGA7: molecular evolutionary genetics analysis version 7.0 for bigger datasets. Mol Biol Evol.

[41] Posada D (2008). jModelTest: phylogenetic model averaging. Mol Biol Evol.

[42] Stamatakis A (2014). RAxML version 8: a tool for phylogenetic analysis and post-analysis of large phylogenies. Bioinformatics.

[43] Drummond AJ, Rambaut A (2007). BEAST: Bayesian evolutionary analysis by sampling trees. BMC Evol Biol.

[44] Helfrich P, Rieb E, Abrami G, Lucking A, Mehler A (2018) TreeAnnotator: versatile visual annotation of hierarchical text relations. In: Lrec 2018: edition of the language resources and evaluation conference; 2018

[45] Huelsenbeck JP, Ronquist F (2001). MRBAYES: Bayesian inference of phylogenetic trees. Bioinformatics.

[46] Hall TA (1999) BioEdit: a user-friendly biological sequence alignment editor and analysis program for Windows 95/98/NT. In: Nucl Acids Symp Ser, pp 95–98

[47] Delport W, Poon AFY, Frost SDW, Kosakovsky Pond SL (2010). Datamonkey 2010: a suite of phylogenetic analysis tools for evolutionary biology. Bioinformatics.

[48] Murrell B, Moola S, Mabona A, Weighill T, Sheward D, Pond SLK, Scheffler K (2013). FUBAR: a fast, unconstrained Bayesian approximation for inferring selection. Mol Biol Evol.

[49] Murrell B, Wertheim JO, Moola S, Weighill T, Scheffler K, Kosakovsky Pond SL (2012). Detecting individual sites subject to episodic diversifying selection. PLoS Genet.

[50] Smith MD, Wertheim JO, Weaver S, Murrell B, Scheffler K, Kosakovsky Pond SL (2015). Less is more: an adaptive branch-site random effects model for efficient detection of episodic diversifying selection. Mol Biol Evol.

[51] Barba M, Daly JM (2016). The influenza NS1 protein: what do we know in equine influenza virus pathogenesis?. Pathogens.

[52] Murcia PR, Baillie GJ, Daly J, Elton D, Jervis C, Mumford JA, Newton R, Parrish CR, Hoelzer K, Dougan G, Parkhill J, Lennard N, Ormond D, Moule S, Whitwham A, McCauley JW, McKinley TJ, Holmes EC, Grenfell BT, Wood JL (2010). Intra- and interhost evolutionary dynamics of equine influenza virus. J Virol.

[53] Westgeest KB, Russell CA, Lin X, Spronken MI, Bestebroer TM, Bahl J, van Beek R, Skepner E, Halpin RA, de Jong JC, Rimmelzwaan GF, Osterhaus AD, Smith DJ, Wentworth DE, Fouchier RA, de Graaf M (2014). Genomewide analysis of reassortment and evolution of human influenza A(H3N2) viruses circulating between 1968 and 2011. J Virol.

[54] Wen F, Blackmon S, Olivier AK, Li L, Guan M, Sun H, Wang PG, Wan XF (2018). Mutation W222L at the receptor binding site of hemagglutinin could facilitate viral adaption from equine influenza A(H3N8) virus to dogs. J Virol.

[55] Rabadan R, Levine AJ, Robins H (2006). Comparison of avian and human influenza A viruses reveals a mutational bias on the viral genomes. J Virol.

[56] Worobey M, Han GZ, Rambaut A (2014). A synchronized global sweep of the internal genes of modern avian influenza virus. Nature.

[57] Newton JR, Daly JM, Spencer L, Mumford JA (2006). Description of the outbreak of equine influenza (H3N8) in the United Kingdom in 2003, during which recently vaccinated horses in Newmarket developed respiratory disease. Vet Rec.

[58] Garcia-Sastre A, Egorov A, Matassov D, Brandt S, Levy DE, Durbin JE, Palese P, Muster T (1998). Influenza A virus lacking the NS1 gene replicates in interferon-deficient systems. Virology.

[59] Geiss GK, Salvatore M, Tumpey TM, Carter VS, Wang X, Basler CF, Taubenberger JK, Bumgarner RE, Palese P, Katze MG, García-Sastre A (2002). Cellular transcriptional profiling in influenza A virus-infected lung epithelial cells: the role of the nonstructural NS1 protein in the evasion of the host innate defense and its potential contribution to pandemic influenza. Proc Natl Acad Sci U S A.

[60] Kwasnik M, Gora IM, Rola J, Zmudzinski JF, Rozek W (2016). NS-gene based phylogenetic analysis of equine influenza viruses isolated in Poland. Vet Microbiol.

[61] Fulvini AA, Ramanunninair M, Le J, Pokorny BA, Arroyo JM, Silverman J, Devis R, Bucher D (2011). Gene constellation of influenza A virus reassortants with high growth phenotype prepared as seed candidates for vaccine production. PLoS One.

[62] Kirkland PD, Finlaison DS, Crispe E, Hurt AC (2010). Influenza virus transmission from horses to dogs, Australia. Emerg Infect Dis.

[63] Hayward JJ, Dubovi EJ, Scarlett JM, Janeczko S, Holmes EC, Parrish CR (2010). Microevolution of canine influenza virus in shelters and its molecular epidemiology in the United States. J Virol.

[64] Rahnama L, Aris-Brosou S (2013). Phylodynamics of the emergence of influenza viruses after cross-species transmission. PLoS One.

[65] Feng W, Sun X, Shi N, Zhang M, Guan Z, Duan M (2017). Influenza a virus NS1 protein induced A20 contributes to viral replication by suppressing interferon-induced antiviral response. Biochem Biophysics Res Commun.

[66] Holmes EC, Ghedin E, Miller N, Taylor J, Bao Y, St George K, Grenfell BT, Salzberg SL, Fraser CM, Lipman DJ, Taubenberger JK (2005). Whole-genome analysis of human influenza A virus reveals multiple persistent lineages and reassortment among recent H3N2 viruses. PLoS Biol.

[67] Jian JW, Lai CT, Kuo CY, Kuo SH, Hsu LC, Chen PJ, Wu HS, Liu MT (2008). Genetic analysis and evaluation of the reassortment of influenza B viruses isolated in Taiwan during the 2004–2005 and 2006–2007 epidemics. Virus Res.

[68] Joseph U, Vijaykrishna D, Smith GJD, Su YCF (2018). Adaptive evolution during the establishment of European avian-like H1N1 influenza A virus in swine. Evol Appl.

[69] Zhu H, Hughes J, Murcia PR (2015). Origins and evolutionary dynamics of H3N2 canine influenza virus. J Virol.

[70] Chen Y, Trovao NS, Wang G, Zhao W, He P, Zhou H, Mo Y, Wei Z, Ouyang K, Huang W, García-Sastre A, Nelson M (2018). Emergence and evolution of novel reassortant influenza A viruses in canines in Southern China. mBio.

