# Supplementary material for: Host-range shift of H3N8 canine influenza virus: a phylodynamic analysis of its origin and adaptation from equine to canine host
Source: Vet Res. 2019 Oct 30;50:87. doi: 10.1186/s13567-019-0707-2 (PMC6822366; doi:10.1186/s13567-019-0707-2)
Supplement: Supplementary file 1 — Additional file 1. List of genetic sequences from multiple influenza A viruses used in this study. [file 13567_2019_707_MOESM1_ESM.docx]

| HA gene | | | | | | | | | |
| --- | --- | --- | --- | --- | --- | --- | --- | --- | --- |
| Isolate_Name | Isolate_Id | | | Segment_Id | | | | | |
| A/canine/California/70645-4/2006 | EPI_ISL_79878 | | | EPI281427 | | | | | |
| A/canine/Colorado/148902/2006 | EPI_ISL_87459 | | | EPI307318 | | | | | |
| A/canine/Colorado/17864/2006 | EPI_ISL_79875 | | | EPI281403 | | | | | |
| A/canine/Colorado/2025974/2007 | EPI_ISL_124090 | | | EPI383599 | | | | | |
| A/canine/Colorado/224766/2006 | EPI_ISL_124091 | | | EPI383600 | | | | | |
| A/canine/Colorado/224986/2006 | EPI_ISL_87458 | | | EPI307317 | | | | | |
| A/canine/Colorado/231256/2007 | EPI_ISL_124092 | | | EPI383601 | | | | | |
| A/canine/Colorado/234550/2009 | EPI_ISL_124093 | | | EPI383602 | | | | | |
| A/canine/Colorado/3/2006 | EPI_ISL_87460 | | | EPI307319 | | | | | |
| A/canine/Colorado/30604/2006 | EPI_ISL_84848 | | | EPI297518 | | | | | |
| A/canine/Colorado/6723-14/2008 | EPI_ISL_82077 | | | EPI289522 | | | | | |
| A/canine/Colorado/6723-8/2008 | EPI_ISL_79901 | | | EPI281611 | | | | | |
| A/canine/Colorado/850078/2009 | EPI_ISL_124094 | | | EPI383603 | | | | | |
| A/canine/Colorado/861142/2010 | EPI_ISL_124095 | | | EPI383604 | | | | | |
| A/canine/Colorado/861997/2010 | EPI_ISL_124096 | | | EPI383605 | | | | | |
| A/canine/Colorado/863160/2010 | EPI_ISL_124097 | | | EPI383606 | | | | | |
| A/canine/Colorado/866907/2010 | EPI_ISL_124098 | | | EPI383607 | | | | | |
| A/canine/Colorado/884753/2010 | EPI_ISL_124099 | | | EPI383608 | | | | | |
| A/canine/Colorado/886050/2010 | EPI_ISL_124100 | | | EPI383609 | | | | | |
| A/canine/Colorado/8880/2006 | EPI_ISL_82095 | | | EPI289520 | | | | | |
| A/canine/Connecticut/85863/2011 | EPI_ISL_294935 | | | EPI1157841 | | | | | |
| A/canine/CT/85863/2011 | EPI_ISL_167698 | | | EPI546819 | | | | | |
| A/canine/Florida/14/2006 | EPI_ISL_68224 | | | EPI237566 | | | | | |
| A/canine/Florida/15592.1/2004 | EPI_ISL_294945 | | | EPI1157860 | | | | | |
| A/canine/Florida/242/2003 | EPI_ISL_9528 | | | EPI98483 | | | | | |
| A/canine/Florida/43/2004 | EPI_ISL_9527 | | | EPI98556 | | | | | |
| A/canine/Florida/61156-2/2006 | EPI_ISL_79895 | | | EPI281563 | | | | | |
| A/canine/Florida/78592-2/2006 | EPI_ISL_79879 | | | EPI281435 | | | | | |
| A/canine/Florida/78592-6/2006 | EPI_ISL_79880 | | | EPI281443 | | | | | |
| A/canine/Florida/78592-7/2006 | EPI_ISL_79881 | | | EPI281451 | | | | | |
| A/canine/Florida/89911-2/2006 | EPI_ISL_79882 | | | EPI281459 | | | | | |
| A/canine/Iowa/13628/2005 | EPI_ISL_9541 | | | EPI98702 | | | | | |
| A/canine/Kentucky/118778/2006 | EPI_ISL_79889 | | | EPI281515 | | | | | |
| A/canine/Maine/058124/2016 | EPI_ISL_294942 | | | EPI1157830 | | | | | |
| A/canine/Massachusetts/26810/2016 | EPI_ISL_234612 | | | EPI838684 | | | | | |
| A/canine/New York/100525-1/2006 | EPI_ISL_79883 | | | EPI281467 | | | | | |
| A/canine/New York/100528-1/2006 | EPI_ISL_79884 | | | EPI281475 | | | | | |
| A/canine/New York/100528-5/2006 | EPI_ISL_79885 | | | EPI281483 | | | | | |
| A/canine/New York/100528-6/2006 | EPI_ISL_79886 | | | EPI281491 | | | | | |
| A/canine/New York/115719/2007 | EPI_ISL_79896 | | | EPI281571 | | | | | |
| A/canine/New York/115809/2005 | EPI_ISL_79864 | | | EPI281315 | | | | | |
| A/canine/New York/12370090/2011 | EPI_ISL_124101 | | | EPI383610 | | | | | |
| A/canine/New York/13258337/2011 | EPI_ISL_124102 | | | EPI383611 | | | | | |
| A/canine/New York/13453967/2011 | EPI_ISL_124103 | | | EPI383612 | | | | | |
| A/canine/New York/13454104/2011 | EPI_ISL_124104 | | | EPI383613 | | | | | |
| A/canine/New York/13693247/2011 | EPI_ISL_124105 | | | EPI383614 | | | | | |
| A/canine/New York/13752393/2011 | EPI_ISL_124106 | | | EPI383615 | | | | | |
| A/canine/New York/13889381/2011 | EPI_ISL_124107 | | | EPI383616 | | | | | |
| A/canine/New York/13949306/2011 | EPI_ISL_124108 | | | EPI383617 | | | | | |
| A/canine/New York/13949315/2011 | EPI_ISL_124109 | | | EPI383618 | | | | | |
| A/canine/New York/145353/2008 | EPI_ISL_79904 | | | EPI281635 | | | | | |
| A/canine/New York/147926-3/2006 | EPI_ISL_79887 | | | EPI281499 | | | | | |
| A/canine/New York/147926-5/2006 | EPI_ISL_79888 | | | EPI281507 | | | | | |
| A/canine/New York/158402-1/2008 | EPI_ISL_79905 | | | EPI281643 | | | | | |
| A/canine/New York/159903/2012 | EPI_ISL_294938 | | | EPI1157829 | | | | | |
| A/canine/New York/1623.1/2010 | EPI_ISL_294943 | | | EPI1157868 | | | | | |
| A/canine/New York/3699/2010 | EPI_ISL_294940 | | | EPI1157865 | | | | | |
| A/canine/New York/3821631/2011 | EPI_ISL_124110 | | | EPI383619 | | | | | |
| A/canine/New York/4986-2/2006 | EPI_ISL_79873 | | | EPI281387 | | | | | |
| A/canine/New York/5183-6/2006 | EPI_ISL_79871 | | | EPI281371 | | | | | |
| A/canine/New York/51854/2008 | EPI_ISL_79902 | | | EPI281619 | | | | | |
| A/canine/New York/6977983/2010 | EPI_ISL_124111 | | | EPI383620 | | | | | |
| A/canine/NY/100525/2006 | EPI_ISL_81918 | | | EPI289524 | | | | | |
| A/canine/NY/100528-4/2006 | EPI_ISL_81920 | | | EPI289519 | | | | | |
| A/canine/NY/105447/2008 | EPI_ISL_174477 | | | EPI576528 | | | | | |
| A/canine/NY/120106.2/2011 | EPI_ISL_166503 | | | EPI546815 | | | | | |
| A/canine/NY/133/2010 | EPI_ISL_167704 | | | EPI546826 | | | | | |
| A/canine/NY/147926/2006 | EPI_ISL_82076 | | | EPI289518 | | | | | |
| A/canine/NY/159903/2012 | EPI_ISL_167700 | | | EPI546821 | | | | | |
| A/canine/NY/1623.1/2010 | EPI_ISL_167705 | | | EPI546827 | | | | | |
| A/canine/NY/3699/2010 | EPI_ISL_167706 | | | EPI546828 | | | | | |
| A/canine/NY/4986/2006 | EPI_ISL_81919 | | | EPI289517 | | | | | |
| A/canine/NY/dog1c01/2009 | EPI_ISL_82037 | | | EPI289558 | | | | | |
| A/canine/NY/dog4c01/2009 | EPI_ISL_82013 | | | EPI289584 | | | | | |
| A/canine/NY/dog4c02/2009 | EPI_ISL_82011 | | | EPI289585 | | | | | |
| A/canine/NY/dog4c05/2009 | EPI_ISL_82122 | | | EPI289586 | | | | | |
| A/canine/NY/dog4c10/2009 | EPI_ISL_82132 | | | EPI289578 | | | | | |
| A/canine/NY/dog4c13/2009 | EPI_ISL_82009 | | | EPI289583 | | | | | |
| A/canine/NY/dog4c14/2009 | EPI_ISL_82133 | | | EPI289575 | | | | | |
| A/canine/NY/dog4c17/2009 | EPI_ISL_82004 | | | EPI289579 | | | | | |
| A/canine/NY/dog4c18/2009 | EPI_ISL_82086 | | | EPI289580 | | | | | |
| A/canine/NY/dog4c20/2009 | EPI_ISL_82005 | | | EPI289581 | | | | | |
| A/canine/NY/dog5c04/2009 | EPI_ISL_82062 | | | EPI289561 | | | | | |
| A/canine/NY/dog6c02/2009 | EPI_ISL_82161 | | | EPI289565 | | | | | |
| A/canine/NY/dog6c08/2009 | EPI_ISL_82169 | | | EPI289567 | | | | | |
| A/canine/NY/dog6c12/2009 | EPI_ISL_82155 | | | EPI289574 | | | | | |
| A/canine/NY/dog6c13/2009 | EPI_ISL_82082 | | | EPI289568 | | | | | |
| A/canine/NY/dog6c15/2009 | EPI_ISL_82156 | | | EPI289573 | | | | | |
| A/canine/NY/dog6c16/2009 | EPI_ISL_82084 | | | EPI289566 | | | | | |
| A/canine/NY/dog6c19/2009 | EPI_ISL_82006 | | | EPI289569 | | | | | |
| A/canine/NY/dog8c01/2008 | EPI_ISL_81969 | | | EPI289525 | | | | | |
| A/canine/NY/dog8c04/2008 | EPI_ISL_82112 | | | EPI289555 | | | | | |
| A/canine/NY/dog8c05/2008 | EPI_ISL_81981 | | | EPI289527 | | | | | |
| A/canine/NY/dog8c06/2008 | EPI_ISL_81972 | | | EPI289528 | | | | | |
| A/canine/NY/dog8c07/2008 | EPI_ISL_81973 | | | EPI289529 | | | | | |
| A/canine/NY/dog8c13/2008 | EPI_ISL_81976 | | | EPI289554 | | | | | |
| A/canine/PA/111788.2/2009 | EPI_ISL_166506 | | | EPI546825 | | | | | |
| A/canine/PA/27637.3/2010 | EPI_ISL_167697 | | | EPI546818 | | | | | |
| A/canine/PA/33225.4/2010 | EPI_ISL_167696 | | | EPI546816 | | | | | |
| A/canine/PA/96978/2009 | EPI_ISL_167699 | | | EPI546820 | | | | | |
| A/canine/Pennsylvania/10909/2007 | EPI_ISL_79891 | | | EPI281531 | | | | | |
| A/canine/Pennsylvania/10915/2007 | EPI_ISL_74767 | | | EPI256924 | | | | | |
| A/canine/Pennsylvania/137154/2008 | EPI_ISL_79903 | | | EPI281627 | | | | | |
| A/canine/Pennsylvania/16699/2007 | EPI_ISL_79890 | | | EPI281523 | | | | | |
| A/canine/Pennsylvania/94930-3/2007 | EPI_ISL_79897 | | | EPI281579 | | | | | |
| A/canine/Pennsylvania/96978/2009 | EPI_ISL_294937 | | | EPI1157820 | | | | | |
| A/canine/South Carolina/89215/2009 | EPI_ISL_124112 | | | EPI383621 | | | | | |
| A/canine/StatenIs/115719/2007 | EPI_ISL_81921 | | | EPI289521 | | | | | |
| A/canine/Sydney/6525/2007 | EPI_ISL_63432 | | | EPI221707 | | | | | |
| A/canine/Sydney/6692/2007 | EPI_ISL_63433 | | | EPI221710 | | | | | |
| A/canine/Texas/1/2004 | EPI_ISL_9535 | | | EPI98568 | | | | | |
| A/canine/Vermont/278213/2013 | EPI_ISL_294941 | | | EPI1157894 | | | | | |
| A/canine/Virginia/93653/2009 | EPI_ISL_82078 | | | EPI289523 | | | | | |
| A/canine/VT/11039/2013 | EPI_ISL_166505 | | | EPI546817 | | | | | |
| A/canine/VT/2782/2013 | EPI_ISL_167701 | | | EPI546822 | | | | | |
| A/canine/Wyoming/86033/2007 | EPI_ISL_87461 | | | EPI307320 | | | | | |
| A/canine/Wyoming/86955/2007 | EPI_ISL_124113 | | | EPI383622 | | | | | |
| A/dog/Jacksonville/C3/2005 | EPI_ISL_18419 | | | EPI149074 | | | | | |
| A/dog/Miami/E3/2005 | EPI_ISL_18420 | | | EPI149075 | | | | | |
| A/equine/Santiago/1/1985 | EPI_ISL_3674 | | | EPI19630 | | | | | |
| A/equine/Kentucky/5/02 | EPI_ISL_4663 | | | EPI25742 | | | | | |
| A/equine/Idaho/37875/1991 | EPI_ISL_6623 | | | EPI52067 | | | | | |
| A/equine/Kentucky/1/1978 | EPI_ISL_8087 | | | EPI77710 | | | | | |
| A/equine/Kentucky/4/1980 | EPI_ISL_8088 | | | EPI77729 | | | | | |
| A/equine/California/1/1980 | EPI_ISL_8089 | | | EPI77748 | | | | | |
| A/equine/Kentucky/2/1981 | EPI_ISL_8090 | | | EPI77767 | | | | | |
| A/equine/Miami/1/1963 | EPI_ISL_8092 | | | EPI129722 | | | | | |
| A/equine/Kentucky/pass_the_pepper1/1976 | EPI_ISL_8093 | | | EPI77824 | | | | | |
| A/equine/Kentucky/bitter_boredom5/1976 | EPI_ISL_8094 | | | EPI77843 | | | | | |
| A/equine/Georgia/1/1981 | EPI_ISL_8095 | | | EPI77862 | | | | | |
| A/equine/Georgia/3/1981 | EPI_ISL_8096 | | | EPI77881 | | | | | |
| A/equine/Georgia/9/1981 | EPI_ISL_8097 | | | EPI77900 | | | | | |
| A/equine/Georgia/10/1981 | EPI_ISL_8098 | | | EPI77919 | | | | | |
| A/equine/Georgia/13/1981 | EPI_ISL_8099 | | | EPI77938 | | | | | |
| A/equine/Kentucky/magnificent_genius1/1981 | EPI_ISL_8100 | | | EPI77957 | | | | | |
| A/equine/California/103/1982 | EPI_ISL_8101 | | | EPI77976 | | | | | |
| A/equine/New York/VR-297/1983 | EPI_ISL_8102 | | | EPI77995 | | | | | |
| A/equine/New York/1/1999 | EPI_ISL_9531 | | | EPI98554 | | | | | |
| A/equine/Kentucky/5/2002 | EPI_ISL_9530 | | | EPI321721 | | | | | |
| A/equine/Ohio/1/2003 | EPI_ISL_9534 | | | EPI98560 | | | | | |
| A/equine/Massachussetts/213/2003 | EPI_ISL_9529 | | | EPI98562 | | | | | |
| A/equine/New York/452/2003 | EPI_ISL_9532 | | | EPI98564 | | | | | |
| A/equine/California/191/2003 | EPI_ISL_9533 | | | EPI98566 | | | | | |
| A/equine/Wisconsin/1/03 | EPI_ISL_9783 | | | EPI99376 | | | | | |
| A/equine/Hong Kong/1/92 | EPI_ISL_8281 | | | EPI129223 | | | | | |
| A/equine/Argentina/1/93 | EPI_ISL_14606 | | | EPI129317 | | | | | |
| A/equine/Florida/1/94 | EPI_ISL_1403 | | | EPI6432 | | | | | |
| A/equine/Kentucky/1/90 | EPI_ISL_14607 | | | EPI129321 | | | | | |
| A/equine/Florida/1/93 | EPI_ISL_14608 | | | EPI129323 | | | | | |
| A/equine/Kentucky/1/92 | EPI_ISL_8282 | | | EPI129325 | | | | | |
| A/Equine/Alaska/1/91 | EPI_ISL_680 | | | EPI129327 | | | | | |
| A/equine/Uruguay/1/1963 | EPI_ISL_21282 | | | EPI154026 | | | | | |
| A/equine/Tennessee/5/1985 | EPI_ISL_14704 | | | EPI129736 | | | | | |
| A/equine/Kentucky/2/1986 | EPI_ISL_14705 | | | EPI129738 | | | | | |
| A/equine/Kentucky/1/1987 | EPI_ISL_14706 | | | EPI129740 | | | | | |
| A/equine/France/1/1976 | EPI_ISL_14810 | | | EPI130345 | | | | | |
| A/equine/Kentucky/1/81 | EPI_ISL_689 | | | EPI130955 | | | | | |
| A/equine/Kentucky/3/1981 | EPI_ISL_15342 | | | EPI133028 | | | | | |
| A/equine/California/83/1982 | EPI_ISL_15343 | | | EPI133047 | | | | | |
| A/equine/Kentucky/1/1986 | EPI_ISL_15344 | | | EPI133066 | | | | | |
| A/equine/Kentucky/692/1988 | EPI_ISL_15345 | | | EPI133104 | | | | | |
| A/equine/Kentucky/694/1988 | EPI_ISL_15346 | | | EPI133123 | | | | | |
| A/equine/Kentucky/698/1988 | EPI_ISL_15347 | | | EPI133142 | | | | | |
| A/equine/Kentucky/1277/1990 | EPI_ISL_15348 | | | EPI133161 | | | | | |
| A/equine/Texas/39655/1991 | EPI_ISL_15349 | | | EPI133180 | | | | | |
| A/equine/Kentucky/1/1992 | EPI_ISL_15350 | | | EPI133199 | | | | | |
| A/equine/Alaska/29759/1991 | EPI_ISL_15351 | | | EPI133218 | | | | | |
| A/equine/Tennessee/5/1986 | EPI_ISL_21061 | | | EPI133237 | | | | | |
| A/equine/Kentucky/1/1991 | EPI_ISL_15352 | | | EPI133256 | | | | | |
| A/equine/Kentucky/8/1994 | EPI_ISL_15353 | | | EPI133275 | | | | | |
| A/equine/New York/1/1983 | EPI_ISL_15356 | | | EPI133332 | | | | | |
| A/equine/Kentucky/Rosie100/1981 | EPI_ISL_15830 | | | EPI137528 | | | | | |
| A/equine/California/4537/1997 | EPI_ISL_15842 | | | EPI137756 | | | | | |
| A/equine/California/8560/2002 | EPI_ISL_15843 | | | EPI137775 | | | | | |
| A/equine/Kentucky/2/1987 | EPI_ISL_19104 | | | EPI152769 | | | | | |
| A/equine/Kanazawa/1/2007 | EPI_ISL_666 | | | EPI153118 | | | | | |
| A/equine/Kentucky/3/1986 | EPI_ISL_19663 | | | EPI153850 | | | | | |
| A/equine/Sao Paulo/6/1963 | EPI_ISL_19670 | | | EPI153914 | | | | | |
| A/equine/Cordoba/18/1985 | EPI_ISL_19671 | | | EPI153922 | | | | | |
| A/equine/Santa Fe/1/1985 | EPI_ISL_19672 | | | EPI153930 | | | | | |
| A/equine/Italy/824/1991 | EPI_ISL_19673 | | | EPI153938 | | | | | |
| A/equine/Italy/1199/1992 | EPI_ISL_19674 | | | EPI153946 | | | | | |
| A/equine/Austria/421/1992 | EPI_ISL_19675 | | | EPI153954 | | | | | |
| A/equine/Switzerland/173/1993 | EPI_ISL_19676 | | | EPI153962 | | | | | |
| A/equine/Roma/5/1991 | EPI_ISL_19677 | | | EPI153970 | | | | | |
| A/equine/Italy/1062/1991 | EPI_ISL_19678 | | | EPI153978 | | | | | |
| A/equine/Switzerland/1118/1979 | EPI_ISL_19679 | | | EPI153986 | | | | | |
| A/equine/Romania/1/1980 | EPI_ISL_19680 | | | EPI153994 | | | | | |
| A/equine/Sao Paulo/1/1969 | EPI_ISL_19681 | | | EPI154002 | | | | | |
| A/equine/Fontainbleu/1/1979 | EPI_ISL_19682 | | | EPI154010 | | | | | |
| A/equine/Berlin/1/1989 | EPI_ISL_19683 | | | EPI154018 | | | | | |
| A/equine/Sussex/1/1989 | EPI_ISL_19884 | | | EPI154452 | | | | | |
| A/equine/Sussex/93753/89 | EPI_ISL_8280 | | | EPI80610 | | | | | |
| A/equine/Kentucky/2/1980 | EPI_ISL_20807 | | | EPI156241 | | | | | |
| A/equine/Algiers/1/1972 | EPI_ISL_20808 | | | EPI156249 | | | | | |
| A/equine/Johannesburg/1/1986 | EPI_ISL_20809 | | | EPI156257 | | | | | |
| A/equine/Guelph/G03-55399/2003 | EPI_ISL_20865 | | | EPI156660 | | | | | |
| A/equine/Guelph/G04-54701/2004 | EPI_ISL_20866 | | | EPI156661 | | | | | |
| A/equine/Guelph/G03-0250/2003 | EPI_ISL_20867 | | | EPI156662 | | | | | |
| A/equine/Guelph/06-28865/2006 | EPI_ISL_20868 | | | EPI156663 | | | | | |
| A/equine/Switzerland/2225/1979 | EPI_ISL_22611 | | | EPI159272 | | | | | |
| A/equine/France/1/1967 | EPI_ISL_22612 | | | EPI159280 | | | | | |
| A/equine/Lonquen/1/2006 | EPI_ISL_23100 | | | EPI161638 | | | | | |
| A/equine/Egypt/6066NAMRU3-VSVRI/2008 | EPI_ISL_24747 | | | EPI164730 | | | | | |
| A/equine/Sachiyama/1/1971 | EPI_ISL_25008 | | | EPI165401 | | | | | |
| A/equine/Newmarket/5/2003 | EPI_ISL_75492 | | | EPI261829 | | | | | |
| A/equine/Gansu/7/2008 | EPI_ISL_32656 | | | EPI186854 | | | | | |
| A/equine/Hubei/6/2008 | EPI_ISL_32657 | | | EPI186862 | | | | | |
| A/equine/Heilongjiang/10/2008 | EPI_ISL_32658 | | | EPI186870 | | | | | |
| A/equine/Liaoning/9/2008 | EPI_ISL_32659 | | | EPI186878 | | | | | |
| A/equine/Inner Mongolia/8/2008 | EPI_ISL_32660 | | | EPI186886 | | | | | |
| A/equine/Qinghai/1/1994 | EPI_ISL_32661 | | | EPI186894 | | | | | |
| A/equine/Xinjiang/1/2007 | EPI_ISL_32662 | | | EPI186902 | | | | | |
| A/equine/Xinjiang/2/2007 | EPI_ISL_32663 | | | EPI186910 | | | | | |
| A/equine/Xinjiang/3/2007 | EPI_ISL_32664 | | | EPI186918 | | | | | |
| A/equine/Xinjiang/4/2007 | EPI_ISL_32665 | | | EPI186926 | | | | | |
| A/equine/Xinjiang/5/2007 | EPI_ISL_32666 | | | EPI186934 | | | | | |
| A/equine/Ibaraki/1/07 | EPI_ISL_63503 | | | EPI222039 | | | | | |
| A/equine/Sydney/6085/2007 | EPI_ISL_63504 | | | EPI222041 | | | | | |
| A/equine/Bari/2005 | EPI_ISL_64877 | | | EPI227206 | | | | | |
| A/TBY/91 | EPI_ISL_68837 | | | EPI239827 | | | | | |
| A/equine/Ibadan/6/91 | EPI_ISL_68846 | | | EPI239849 | | | | | |
| A/equine/Ibadan/9/91 | EPI_ISL_68847 | | | EPI239850 | | | | | |
| A/equine/Kentucky/1/1981 | EPI_ISL_68957 | | | EPI240110 | | | | | |
| A/equine/Kentucky/211/1987 | EPI_ISL_68958 | | | EPI240118 | | | | | |
| A/equine/Romania/1980 | EPI_ISL_68961 | | | EPI240127 | | | | | |
| A/equine/Suffolk/89 | EPI_ISL_68962 | | | EPI240128 | | | | | |
| A/equine/Hokkaido/I828/2008 | EPI_ISL_69119 | | | EPI240682 | | | | | |
| A/equine/Yokohama/aq19/2009 | EPI_ISL_69743 | | | EPI241775 | | | | | |
| A/equine/Algiers/1972 | EPI_ISL_69895 | | | EPI242347 | | | | | |
| A/equine/Fontainebleau/1976 | EPI_ISL_69896 | | | EPI242348 | | | | | |
| A/equine/Kascakew/1/1978 | EPI_ISL_69898 | | | EPI242353 | | | | | |
| A/equine/Miami/1963 | EPI_ISL_69900 | | | EPI242359 | | | | | |
| A/equine/New Market/1976 | EPI_ISL_69901 | | | EPI242360 | | | | | |
| A/equine/Tokyo/1971 | EPI_ISL_69906 | | | EPI242371 | | | | | |
| A/equine/Huabei/1/2007 | EPI_ISL_70195 | | | EPI243547 | | | | | |
| A/equine/Almaty/26/2007 | EPI_ISL_71977 | | | EPI250068 | | | | | |
| A/equine/Ahmedabad/1/2009 | EPI_ISL_74364 | | | EPI255566 | | | | | |
| A/equine/Mysore/1/2008 | EPI_ISL_74367 | | | EPI255570 | | | | | |
| A/equine/Katra-Jammu/6/2008 | EPI_ISL_154972 | | | EPI265942 | | | | | |
| A/equine/Texas/117793/2005 | EPI_ISL_79867 | | | EPI281339 | | | | | |
| A/equine/Ohio/113461-2/2005 | EPI_ISL_79866 | | | EPI281331 | | | | | |
| A/equine/Ohio/113461-1/2005 | EPI_ISL_79869 | | | EPI281355 | | | | | |
| A/equine/Ohio/113461-3/2005 | EPI_ISL_79870 | | | EPI281363 | | | | | |
| A/equine/Montana/9233/2007 | EPI_ISL_79893 | | | EPI281547 | | | | | |
| A/equine/Virginia/131054-3/2005 | EPI_ISL_79899 | | | EPI281595 | | | | | |
| A/equine/New York/146066/2007 | EPI_ISL_79900 | | | EPI281603 | | | | | |
| A/equine/Spain/1/2007 | EPI_ISL_81729 | | | EPI288543 | | | | | |
| A/equine/Colorado/10/2007 | EPI_ISL_87462 | | | EPI307321 | | | | | |
| A/equine/Florida/612/2004 | EPI_ISL_89154 | | | EPI314107 | | | | | |
| A/equine/Florida/779/2004 | EPI_ISL_89155 | | | EPI314110 | | | | | |
| A/equine/Otar/764/2007 | EPI_ISL_89156 | | | EPI314112 | | | | | |
| A/equine/Yokohama/aq13/2010 | EPI_ISL_297955 | | | EPI1172954 | | | | | |
| A/equine/Tottori/1/07 | EPI_ISL_89221 | | | EPI314449 | | | | | |
| A/equine/Tokyo/2/1971 | EPI_ISL_95197 | | | EPI332914 | | | | | |
| A/equine/Donegal/1/2007 | EPI_ISL_100144 | | | EPI347149 | | | | | |
| A/equine/Meath/1/2007 | EPI_ISL_100145 | | | EPI347150 | | | | | |
| A/equine/Kildare/1/2007 | EPI_ISL_100146 | | | EPI347151 | | | | | |
| A/equine/Down/1/2008 | EPI_ISL_100147 | | | EPI347152 | | | | | |
| A/equine/Donegal/1/2009 | EPI_ISL_100148 | | | EPI347153 | | | | | |
| A/equine/Limerick/1/2010 | EPI_ISL_100150 | | | EPI347155 | | | | | |
| A/equine/Carlow/1/2009 | EPI_ISL_100151 | | | EPI347156 | | | | | |
| A/equine/Heilongjiang/1/2010 | EPI_ISL_103105 | | | EPI352393 | | | | | |
| A/equine/Nador/1/1997 | EPI_ISL_119931 | | | EPI372051 | | | | | |
| A/equine/Essaouira/2/2004 | EPI_ISL_119932 | | | EPI372053 | | | | | |
| A/equine/Yokohama/aq5/2011 | EPI_ISL_121370 | | | EPI376180 | | | | | |
| A/equine/Yokohama/aq29/2011 | EPI_ISL_121371 | | | EPI376182 | | | | | |
| A/equine/Yokohama/aq53/2011 | EPI_ISL_121372 | | | EPI376184 | | | | | |
| A/equine/Yokohama/aq79/2011 | EPI_ISL_121373 | | | EPI376186 | | | | | |
| A/equine/Mongolia/3/2011 | EPI_ISL_129371 | | | EPI395970 | | | | | |
| A/equine/Mongolia/20/2011 | EPI_ISL_129372 | | | EPI395971 | | | | | |
| A/equine/Mongolia/56/2011 | EPI_ISL_129373 | | | EPI395973 | | | | | |
| A/equine/Kyonggi/SA1/2011 | EPI_ISL_129971 | | | EPI398887 | | | | | |
| A/equine/Tiaret/1/2011 | EPI_ISL_144944 | | | EPI465042 | | | | | |
| A/equine/Tiaret/2/2011 | EPI_ISL_144945 | | | EPI465043 | | | | | |
| A/equine/Tiaret/3/2011 | EPI_ISL_144946 | | | EPI465044 | | | | | |
| A/equine/Tiaret/4/2011 | EPI_ISL_144947 | | | EPI465045 | | | | | |
| A/equine/Tiaret/5/2011 | EPI_ISL_144948 | | | EPI465046 | | | | | |
| A/equine/Tiaret/6/2011 | EPI_ISL_144949 | | | EPI465047 | | | | | |
| A/equine/Tiaret/7/2011 | EPI_ISL_144950 | | | EPI465048 | | | | | |
| A/equine/Tiaret/8/2011 | EPI_ISL_144951 | | | EPI465049 | | | | | |
| A/equine/Tiaret/9/2011 | EPI_ISL_144952 | | | EPI465050 | | | | | |
| A/equine/Tiaret/10/2011 | EPI_ISL_144953 | | | EPI465051 | | | | | |
| A/equine/Xuzhou/01/2013 | EPI_ISL_151130 | | | EPI489630 | | | | | |
| A/equine/Ayrshire/1/2013 | EPI_ISL_151796 | | | EPI492691 | | | | | |
| A/equine/Hertfordshire/1/2013 | EPI_ISL_151797 | | | EPI492692 | | | | | |
| A/equine/Northamptonshire/1/2013 | EPI_ISL_151841 | | | EPI492817 | | | | | |
| A/equine/Shropshire/1/2013 | EPI_ISL_151842 | | | EPI492818 | | | | | |
| A/equine/Shropshire/2/2013 | EPI_ISL_151843 | | | EPI492819 | | | | | |
| A/equine/Worcestershire/1/2013 | EPI_ISL_151845 | | | EPI492821 | | | | | |
| A/equine/Warwickshire/1/2013 | EPI_ISL_151846 | | | EPI492822 | | | | | |
| A/equine/East Yorkshire/1/2013 | EPI_ISL_151847 | | | EPI492823 | | | | | |
| A/equine/Lincolnshire/1/2013 | EPI_ISL_151848 | | | EPI492824 | | | | | |
| A/equine/Lanarkshire/1/2013 | EPI_ISL_151981 | | | EPI493612 | | | | | |
| A/equine/Lanarkshire/2/2013 | EPI_ISL_151982 | | | EPI493614 | | | | | |
| A/equine/Northamptonshire/7/2013 | EPI_ISL_151984 | | | EPI493616 | | | | | |
| A/eqine/Shropshire/5/2013 | EPI_ISL_151985 | | | EPI493619 | | | | | |
| A/equine/Shropshire/8/2013 | EPI_ISL_151986 | | | EPI493622 | | | | | |
| A/equine/Heilongjiang/SS1/2013 | EPI_ISL_161474 | | | EPI527593 | | | | | |
| A/equine/South Kazakhstan/236/2012 | EPI_ISL_166607 | | | EPI542619 | | | | | |
| A/equine/FL/146609/2011 | EPI_ISL_167702 | | | EPI546823 | | | | | |
| A/equine/OR/78356/2012 | EPI_ISL_167703 | | | EPI546824 | | | | | |
| A/equine/Baizak/09/2012 | EPI_ISL_170787 | | | EPI558694 | | | | | |
| A/equine/LKZ/09/2012 | EPI_ISL_170788 | | | EPI558696 | | | | | |
| A/equine/Matybulak/10/2012 | EPI_ISL_170789 | | | EPI558697 | | | | | |
| A/equine/Kostanay/09/2012 | EPI_ISL_170790 | | | EPI558698 | | | | | |
| A/equine/Guangxi/1/2008 | EPI_ISL_174545 | | | EPI573752 | | | | | |
| A/equine/Switzerland/P112/07 | EPI_ISL_177468 | | | EPI584116 | | | | | |
| A/equine/Lincolnshire/06 | EPI_ISL_177471 | | | EPI584127 | | | | | |
| A/equine/Cheshire/06 | EPI_ISL_177472 | | | EPI584135 | | | | | |
| A/equine/Moulton/98 | EPI_ISL_177487 | | | EPI584173 | | | | | |
| A/equine/Snailwell/98 | EPI_ISL_177489 | | | EPI584187 | | | | | |
| A/equine/Lanarkshire/09 | EPI_ISL_177490 | | | EPI584195 | | | | | |
| A/equine/Dorset/09 | EPI_ISL_177491 | | | EPI584203 | | | | | |
| A/equine/Perthshire/3/09 | EPI_ISL_177492 | | | EPI584211 | | | | | |
| A/equine/Yorkshire/3/09 | EPI_ISL_156745 | | | EPI584219 | | | | | |
| A/equine/Shropshire/10 | EPI_ISL_156748 | | | EPI584227 | | | | | |
| A/equine/East Renfrewshire/2/11 | EPI_ISL_156750 | | | EPI584236 | | | | | |
| A/equine/Devon/1/11 | EPI_ISL_156749 | | | EPI584244 | | | | | |
| A/equine/California/1/10 | EPI_ISL_177498 | | | EPI584270 | | | | | |
| A/equine/Kentucky/1/11 | EPI_ISL_177500 | | | EPI584278 | | | | | |
| A/equine/Dubai/1/12 | EPI_ISL_177501 | | | EPI584286 | | | | | |
| A/equine/Rio Grande do Sul/1/12 | EPI_ISL_177502 | | | EPI584295 | | | | | |
| A/Equine/Sweden/SVA111206SZ0085/VIR165837/2011 | EPI_ISL_180684 | | | EPI594013 | | | | | |
| A/Equine/Sweden/SVA111206SZ0085VIR165837/2011 | EPI_ISL_190419 | | | EPI620254 | | | | | |
| A Equine Sweden SVA111208SZ0077VIR167905 2011 | EPI_ISL_190420 | | | EPI620256 | | | | | |
| #A Equine Sweden SVA111208SZ0077VIR167906 2011 | EPI_ISL_190421 | | | EPI620258 | | | | | |
| A Equine Sweden SVA111212SZ0058VIR169816 2011 | EPI_ISL_190422 | | | EPI620260 | | | | | |
| A Equine Sweden SVA111209SZ0099VIR169118 2011 | EPI_ISL_190423 | | | EPI620262 | | | | | |
| A/Equine/Sweden/SVA111209SZ0099VIR169122/2011 | EPI_ISL_190424 | | | EPI620264 | | | | | |
| A/Equine/Sweden/SVA111209SZ0099VIR169124/2011 | EPI_ISL_190425 | | | EPI620266 | | | | | |
| A/Equine/Sweden/SVA111209SZ0099VIR169126/2011 | EPI_ISL_190426 | | | EPI620268 | | | | | |
| A/Equine/Sweden/SVA111209SZ0099VIR169128/2011 | EPI_ISL_190427 | | | EPI620270 | | | | | |
| A/Equine/Sweden/SVA111209SZ0099VIR169130/2011 | EPI_ISL_190428 | | | EPI620272 | | | | | |
| A/Equine/Sweden/SVA111209SZ0099VIR169132/2011 | EPI_ISL_190429 | | | EPI620274 | | | | | |
| A/Equine/Sweden/SVA111212SZ0056VIR169815/2011 | EPI_ISL_190430 | | | EPI620276 | | | | | |
| A/Equine/Sweden/SVA111213SZ0065VIR170334/2011 | EPI_ISL_190431 | | | EPI620278 | | | | | |
| A/Equine/Sweden/SVA111213SZ0065VIR170335/2011 | EPI_ISL_190432 | | | EPI620280 | | | | | |
| A/Equine/Sweden/SVA111213SZ0070VIR170336/2011 | EPI_ISL_190433 | | | EPI620282 | | | | | |
| A/Equine/Sweden/SVA111213SZ0070VIR170337/2011 | EPI_ISL_190434 | | | EPI620284 | | | | | |
| A/Equine/Sweden/SVA111219SZ0079VIR174292/2011 | EPI_ISL_190435 | | | EPI620286 | | | | | |
| A/Equine/Sweden/SVA111219SZ0079VIR174293/2011 | EPI_ISL_190436 | | | EPI620288 | | | | | |
| A/Equine/Sweden/SVA111220SZ0255VIR175145/2011 | EPI_ISL_190437 | | | EPI620290 | | | | | |
| A/Equine/Sweden/SVA111221SZ0085BKT087424/2011 | EPI_ISL_190438 | | | EPI620292 | | | | | |
| A/Equine/Sweden/SVA111222SZ0168VIR177856/2011 | EPI_ISL_190439 | | | EPI620294 | | | | | |
| A/Equine/Sweden/SVA111128SZ0073VIR175146/2011 | EPI_ISL_190440 | | | EPI620296 | | | | | |
| A Equine Sweden SVA111128SZ0073VIR160172 2011 | EPI_ISL_190441 | | | EPI620298 | | | | | |
| A/equine/Jammu and Kashmir/CMVL-LEH4/2008 | EPI_ISL_190902 | | | EPI621730 | | | | | |
| A/equine/Jammu and Kashmir/CMVL-LEH6/2008 | EPI_ISL_190903 | | | EPI621736 | | | | | |
| A/equine/Himachal Pradesh/CMVL-YOL2/2008 | EPI_ISL_190904 | | | EPI621743 | | | | | |
| A/equine/South Lanarkshire/1/2015 | EPI_ISL_195790 | | | EPI643192 | | | | | |
| A/equine/Pulawy/1/2008 | EPI_ISL_168012 | | | EPI647558 | | | | | |
| A/equine/West Midlands/1/2015 | EPI_ISL_197571 | | | EPI650051 | | | | | |
| A/equine/Lanarkshire/2/2015 | EPI_ISL_197572 | | | EPI650053 | | | | | |
| A/equine/Tyne&Wear/1/2015 | EPI_ISL_197574 | | | EPI650056 | | | | | |
| A/equine/Tyne&Wear/2/2015 | EPI_ISL_197573 | | | EPI651395 | | | | | |
| A/equine/Buckinghamshire/1/2014 | EPI_ISL_197753 | | | EPI651398 | | | | | |
| A/Equine/Malaysia/M201-1/2015 | EPI_ISL_200992 | | | EPI669718 | | | | | |
| A/Equine/Malaysia/M201-2/2015 | EPI_ISL_200993 | | | EPI669719 | | | | | |
| A/equine/Malaysia/M201-1/2015 | EPI_ISL_200992 | | | EPI669718 | | | | | |
| A/equine/Malaysia/M201-2/2015 | EPI_ISL_200993 | | | EPI669719 | | | | | |
| A/equine/Kent/1/2015 | EPI_ISL_201991 | | | EPI673530 | | | | | |
| A/equine/East Sussex/1/2015 | EPI_ISL_201992 | | | EPI673532 | | | | | |
| A/equine/Norfolk/1/2015 | EPI_ISL_205763 | | | EPI686736 | | | | | |
| A/equine/North Yorkshire/1/2015 | EPI_ISL_205764 | | | EPI686738 | | | | | |
| A/equine/Northamptonshire/2/2015 | EPI_ISL_205765 | | | EPI686740 | | | | | |
| A/equine/Leicestershire/1/2015 | EPI_ISL_205957 | | | EPI687475 | | | | | |
| A/Equine/Kentucky/1/2014 | EPI_ISL_206059 | | | EPI687835 | | | | | |
| A/equine/North Yorkshire/2/2015 | EPI_ISL_207225 | | | EPI694841 | | | | | |
| A/equine/Scottish Borders/3/2015 | EPI_ISL_212044 | | | EPI710985 | | | | | |
| A/equine/Tennessee/28A/2014 | EPI_ISL_220480 | | | EPI753425 | | | | | |
| A/equine/Tennessee/4A/2014 | EPI_ISL_220481 | | | EPI753433 | | | | | |
| A/equine/Tennessee/30A/2014 | EPI_ISL_220482 | | | EPI753439 | | | | | |
| A/equine/Tennessee/28B/2014 | EPI_ISL_220484 | | | EPI753451 | | | | | |
| A/equine/Tennessee/29A/2014 | EPI_ISL_220485 | | | EPI753457 | | | | | |
| A/equine/Tennessee/27A/2014 | EPI_ISL_220483 | | | EPI753470 | | | | | |
| A/equine/Kent/1/2016 | EPI_ISL_224527 | | | EPI773589 | | | | | |
| A/equine/Hampshire/3/2016 | EPI_ISL_231828 | | | EPI824114 | | | | | |
| A/equine/Ayrshire/2/2013 | EPI_ISL_234614 | | | EPI838696 | | | | | |
| A/equine/Northamptonshire/3/2013 | EPI_ISL_234617 | | | EPI838702 | | | | | |
| A/equine/Northamptonshire/5/2013 | EPI_ISL_234645 | | | EPI838916 | | | | | |
| A/Lincolnshire/1/2013 | EPI_ISL_234646 | | | EPI838918 | | | | | |
| A/equine/West Lothian/1/2014 | EPI_ISL_234908 | | | EPI839642 | | | | | |
| A/equine/Perthshire/1/2014 | EPI_ISL_234911 | | | EPI839711 | | | | | |
| A/equine/Staffordshire/1/2014 | EPI_ISL_234912 | | | EPI839713 | | | | | |
| A/equine/Scottish Borders/1/2014 | EPI_ISL_234913 | | | EPI839715 | | | | | |
| A/equine/Perthshire/2/2014 | EPI_ISL_234914 | | | EPI839717 | | | | | |
| A/equine/Worcestershire/2/2014 | EPI_ISL_234915 | | | EPI839719 | | | | | |
| A/equine/Kent/1/2014 | EPI_ISL_234916 | | | EPI839721 | | | | | |
| A/equine/West Midlands/1/2014 | EPI_ISL_234917 | | | EPI839723 | | | | | |
| A/equine/Northamptonshire/3/2015 | EPI_ISL_234921 | | | EPI839794 | | | | | |
| A/equine/Northamptonshire/4/2015 | EPI_ISL_234922 | | | EPI839821 | | | | | |
| A/equine/Argentina/E37-2/1993 | EPI_ISL_237063 | | | EPI855151 | | | | | |
| A/equine/Argentina/E37-18/1993 | EPI_ISL_237064 | | | EPI855152 | | | | | |
| A/equine/Argentina/E41-2/1993 | EPI_ISL_237065 | | | EPI855153 | | | | | |
| A/equine/Argentina/E131/1994 | EPI_ISL_237066 | | | EPI855154 | | | | | |
| A/equine/Argentina/E226/1995 | EPI_ISL_237067 | | | EPI855155 | | | | | |
| A/equine/Argentina/E433/1997 | EPI_ISL_237068 | | | EPI855156 | | | | | |
| A/equine/Argentina/E750/1999 | EPI_ISL_237069 | | | EPI855157 | | | | | |
| A/equine/Argentina/E1134-7/2001 | EPI_ISL_237070 | | | EPI855158 | | | | | |
| A/equine/Argentina/E652/1999 | EPI_ISL_237071 | | | EPI855159 | | | | | |
| A/equine/Argentina/E2345-5/2012 | EPI_ISL_237072 | | | EPI855160 | | | | | |
| A/equine/Argentina/E2357/2012 | EPI_ISL_237073 | | | EPI855161 | | | | | |
| A/equine/Argentina/E2397-2/2012 | EPI_ISL_237074 | | | EPI855162 | | | | | |
| A/equine/Argentina/E2345-1/2012 | EPI_ISL_237075 | | | EPI855163 | | | | | |
| A/equine/Argentina/E4581-3/2004 | EPI_ISL_237076 | | | EPI855164 | | | | | |
| A/equine/Argentina/E3146-1/2012 | EPI_ISL_237077 | | | EPI855165 | | | | | |
| A/equine/Argentina/E6189-3/2005 | EPI_ISL_237078 | | | EPI855166 | | | | | |
| A/equine/Argentina/E6424-2/2005 | EPI_ISL_237079 | | | EPI855167 | | | | | |
| A/equine/Argentina/E6424-1/2005 | EPI_ISL_237080 | | | EPI855168 | | | | | |
| A/equine/Argentina/E2397-3/2012 | EPI_ISL_237081 | | | EPI855169 | | | | | |
| A/equine/Uruguay/E1918-1/2012 | EPI_ISL_237082 | | | EPI855170 | | | | | |
| A/Equine/Ayrshire/1/2013 | EPI_ISL_151796 | | | EPI492691 | | | | | |
| A/Equine/East Sussex/1/2015 | EPI_ISL_201992 | | | EPI673532 | | | | | |
| A/Equine/Kent/1/15 | EPI_ISL_240663 | | | EPI873593 | | | | | |
| A/Equine/South Africa/4/03 | EPI_ISL_240665 | | | EPI873601 | | | | | |
| A/Equine/Hampshire/4/2016 | EPI_ISL_240664 | | | EPI957606 | | | | | |
| A/Equine/Kent/1/16 | EPI_ISL_255630 | | | EPI957608 | | | | | |
| A/Equine/Fife/1/16 | EPI_ISL_255632 | | | EPI957622 | | | | | |
| A/Equine/Stirlingshire/1/16 | EPI_ISL_255633 | | | EPI957634 | | | | | |
| A/Equine/Lancashire/1/16 | EPI_ISL_255635 | | | EPI957669 | | | | | |
| A/Equine/Gloucestershire/2/16 | EPI_ISL_255686 | | | EPI957813 | | | | | |
| A/Equine/Gloucestershire/3/16 | EPI_ISL_255687 | | | EPI957816 | | | | | |
| A/equine/France/1/1973 | EPI_ISL_257658 | | | EPI968999 | | | | | |
| A/equine/Chantilly/1/1975 | EPI_ISL_257659 | | | EPI969000 | | | | | |
| A/equine/Marseille/1/1979 | EPI_ISL_257660 | | | EPI969001 | | | | | |
| A/equine/Bordeaux/1/1983 | EPI_ISL_257661 | | | EPI969002 | | | | | |
| A/equine/Fontainebleau/1/1983 | EPI_ISL_257662 | | | EPI969003 | | | | | |
| A/equine/Blois/1/1983 | EPI_ISL_257663 | | | EPI969004 | | | | | |
| A/equine/Grosbois/1/1984 | EPI_ISL_257664 | | | EPI969005 | | | | | |
| A/equine/France/1/1985 | EPI_ISL_257665 | | | EPI969006 | | | | | |
| A/equine/France/2/1985 | EPI_ISL_257666 | | | EPI969007 | | | | | |
| A/equine/France/3/1985 | EPI_ISL_257667 | | | EPI969008 | | | | | |
| A/equine/France/1/1986 | EPI_ISL_257668 | | | EPI969009 | | | | | |
| A/equine/France/1/1987 | EPI_ISL_257669 | | | EPI969010 | | | | | |
| A/equine/France/1/1991 | EPI_ISL_257670 | | | EPI969011 | | | | | |
| A/equine/Grosbois/1/1993 | EPI_ISL_257671 | | | EPI969012 | | | | | |
| A/equine/Grosbois/1/1997 | EPI_ISL_257672 | | | EPI969013 | | | | | |
| A/equine/Grosbois/1/1999 | EPI_ISL_257673 | | | EPI969014 | | | | | |
| A/equine/Grosbois/2/1999 | EPI_ISL_257674 | | | EPI969015 | | | | | |
| A/equine/Cagnes-sur-Mer/2/2000 | EPI_ISL_257675 | | | EPI969016 | | | | | |
| A/equine/Grosbois/1/2003 | EPI_ISL_257676 | | | EPI969017 | | | | | |
| A/equine/Belfond/6-2/2009 | EPI_ISL_257677 | | | EPI969018 | | | | | |
| A/equine/Cagnes-sur-Mer/2/2011 | EPI_ISL_257678 | | | EPI969019 | | | | | |
| A/equine/Neuville-Pres-Sees/1/2011 | EPI_ISL_257679 | | | EPI969020 | | | | | |
| A/equine/Cambremer/1/2012 | EPI_ISL_257680 | | | EPI969021 | | | | | |
| A/equine/Ain/1/2014 | EPI_ISL_257681 | | | EPI969022 | | | | | |
| A/equine/Gironde/1/2014 | EPI_ISL_257682 | | | EPI969023 | | | | | |
| A/equine/Saone-et-Loire/1/2015 | EPI_ISL_257683 | | | EPI969024 | | | | | |
| A/equine/Yokohama/aq100/2017 | EPI_ISL_263486 | | | EPI998471 | | | | | |
| A/equine/Sao Paulo/16.19/2012 | EPI_ISL_279063 | | | EPI1065020 | | | | | |
| A/equine/Sao Paulo/24.19/2012 | EPI_ISL_279064 | | | EPI1065021 | | | | | |
| A/equine/Sao Paulo/1.19/2012 | EPI_ISL_279065 | | | EPI1065022 | | | | | |
| A/equine/Ankara/1/2013 | EPI_ISL_281349 | | | EPI1076906 | | | | | |
| A/donkey/Shandong/1/2017 | EPI_ISL_284681 | | | EPI1098480 | | | | | |
| A/equine/Montana/9564-1/2015 | EPI_ISL_285417 | | | EPI1103408 | | | | | |
| A/equine/Sao_Paulo/8.FMVZ/2015 | EPI_ISL_285498 | | | EPI1103598 | | | | | |
| A/equine/Sao_Paulo/10.FMVZ/2015 | EPI_ISL_285491 | | | EPI1103583 | | | | | |
| A/equine/Sao_Paulo/11.FMVZ/2015 | EPI_ISL_285492 | | | EPI1103584 | | | | | |
| A/equine/Sao_Paulo/12.FMVZ/2015 | EPI_ISL_285493 | | | EPI1103585 | | | | | |
| A/equine/Sao_Paulo/15.FMVZ/2015 | EPI_ISL_285500 | | | EPI1103606 | | | | | |
| A/equine/Sao_Paulo/25.FMVZ/2015 | EPI_ISL_285495 | | | EPI1103587 | | | | | |
| A/equine/Sao_Paulo/32.FMVZ/2015 | EPI_ISL_285497 | | | EPI1103589 | | | | | |
| A/equine/Georgia/121362-16/2016 | EPI_ISL_294939 | | | EPI1157812 | | | | | |
| A/equine/New York/135857/2016 | EPI_ISL_294936 | | | EPI1157856 | | | | | |
| A/equine/Oregon/78356/2012 | EPI_ISL_294934 | | | EPI1157878 | | | | | |
| A/equine/Florida/146609/2011 | EPI_ISL_294944 | | | EPI1157899 | | | | | |
| A/equine/Ibaraki/1/2007 | EPI_ISL_297956 | | | EPI1172962 | | | | | |
| M1 | | | | | |  |  |  |  |
| Isolate_Name | Isolate_Id | | | Segment_Id | |  |  |  |  |
| A/canine/California/70645-4/2006 | EPI_ISL_79878 | | | EPI281430 | |  |  |  |  |
| A/canine/Colorado/17864/2006 | EPI_ISL_79875 | | | EPI281406 | |  |  |  |  |
| A/canine/Colorado/17864/2006 | EPI_ISL_79875 | | | EPI281406 | |  |  |  |  |
| A/canine/Colorado/30604/2006 | EPI_ISL_79877 | | | EPI281422 | |  |  |  |  |
| A/canine/Colorado/6723-14/2008 | EPI_ISL_82077 | | | EPI289859 | |  |  |  |  |
| A/canine/Colorado/6723-8/2008 | EPI_ISL_79901 | | | EPI281614 | |  |  |  |  |
| A/canine/Colorado/6723-8/2008 | EPI_ISL_79901 | | | EPI281614 | |  |  |  |  |
| A/canine/Colorado/8880/2006 | EPI_ISL_82095 | | | EPI289858 | |  |  |  |  |
| A/canine/Colorado/8880/2006 | EPI_ISL_82095 | | | EPI289858 | |  |  |  |  |
| A/canine/Connecticut/85863/2011 | EPI_ISL_294935 | | | EPI1157821 | |  |  |  |  |
| A/canine/CT/85863/2011 | EPI_ISL_167698 | | | EPI546833 | |  |  |  |  |
| A/canine/Florida/15592.1/2004 | EPI_ISL_294945 | | | EPI1157843 | |  |  |  |  |
| A/canine/Florida/242/2003 | EPI_ISL_9528 | | | EPI98489 | |  |  |  |  |
| A/canine/Florida/43/2004 | EPI_ISL_9527 | | | EPI98471 | |  |  |  |  |
| A/canine/Florida/61156-2/2006 | EPI_ISL_79895 | | | EPI281566 | |  |  |  |  |
| A/canine/Florida/61156-2/2006 | EPI_ISL_79895 | | | EPI281566 | |  |  |  |  |
| A/canine/Florida/78592-2/2006 | EPI_ISL_79879 | | | EPI281438 | |  |  |  |  |
| A/canine/Florida/78592-6/2006 | EPI_ISL_79880 | | | EPI281446 | |  |  |  |  |
| A/canine/Florida/78592-7/2006 | EPI_ISL_79881 | | | EPI281454 | |  |  |  |  |
| A/canine/Florida/89911-2/2006 | EPI_ISL_79882 | | | EPI281462 | |  |  |  |  |
| A/canine/Jacksonville/2005 | EPI_ISL_64818 | | | EPI226911 | |  |  |  |  |
| A/canine/Kentucky/118778/2006 | EPI_ISL_79889 | | | EPI281518 | |  |  |  |  |
| A/canine/Maine/058124/2016 | EPI_ISL_294942 | | | EPI1157896 | |  |  |  |  |
| A/canine/Massachusetts/26810/2016 | EPI_ISL_234612 | | | EPI839089 | |  |  |  |  |
| A/canine/Miami/2005 | EPI_ISL_64819 | | | EPI226917 | |  |  |  |  |
| A/canine/New York/100525-1/2006 | EPI_ISL_79883 | | | EPI281470 | |  |  |  |  |
| A/canine/New York/100528-1/2006 | EPI_ISL_79884 | | | EPI281478 | |  |  |  |  |
| A/canine/New York/100528-5/2006 | EPI_ISL_79885 | | | EPI281486 | |  |  |  |  |
| A/canine/New York/100528-6/2006 | EPI_ISL_79886 | | | EPI281494 | |  |  |  |  |
| A/canine/New York/115719/2007 | EPI_ISL_79896 | | | EPI281574 | |  |  |  |  |
| A/canine/New York/115809/2005 | EPI_ISL_79864 | | | EPI281318 | |  |  |  |  |
| A/canine/New York/145353/2008 | EPI_ISL_79904 | | | EPI281638 | |  |  |  |  |
| A/canine/New York/147926-3/2006 | EPI_ISL_79887 | | | EPI281502 | |  |  |  |  |
| A/canine/New York/147926-5/2006 | EPI_ISL_79888 | | | EPI281510 | |  |  |  |  |
| A/canine/New York/158402-1/2008 | EPI_ISL_79905 | | | EPI281646 | |  |  |  |  |
| A/canine/New York/159903/2012 | EPI_ISL_294938 | | | EPI1157858 | |  |  |  |  |
| A/canine/New York/1623.1/2010 | EPI_ISL_294943 | | | EPI1157869 | |  |  |  |  |
| A/canine/New York/3699/2010 | EPI_ISL_294940 | | | EPI1157890 | |  |  |  |  |
| A/canine/New York/4986-2/2006 | EPI_ISL_79873 | | | EPI281390 | |  |  |  |  |
| A/canine/New York/5183-6/2006 | EPI_ISL_79871 | | | EPI281374 | |  |  |  |  |
| A/canine/New York/5183-6/2006 | EPI_ISL_79871 | | | EPI281374 | |  |  |  |  |
| A/canine/New York/51854/2008 | EPI_ISL_79902 | | | EPI281622 | |  |  |  |  |
| A/canine/NY/100525/2006 | EPI_ISL_81918 | | | EPI288998 | |  |  |  |  |
| A/canine/NY/100528-4/2006 | EPI_ISL_81920 | | | EPI289000 | |  |  |  |  |
| A/canine/NY/105447/2008 | EPI_ISL_174477 | | | EPI576531 | |  |  |  |  |
| A/canine/NY/120106.2/2011 | EPI_ISL_166503 | | | EPI546829 | |  |  |  |  |
| A/canine/NY/133/2010 | EPI_ISL_167704 | | | EPI546840 | |  |  |  |  |
| A/canine/NY/147926/2006 | EPI_ISL_82076 | | | EPI289862 | |  |  |  |  |
| A/canine/NY/159903/2012 | EPI_ISL_167700 | | | EPI546835 | |  |  |  |  |
| A/canine/NY/1623.1/2010 | EPI_ISL_167705 | | | EPI546841 | |  |  |  |  |
| A/canine/NY/3699/2010 | EPI_ISL_167706 | | | EPI546842 | |  |  |  |  |
| A/canine/NY/4986/2006 | EPI_ISL_81919 | | | EPI288999 | |  |  |  |  |
| A/canine/NY/dog1c01/2009 | EPI_ISL_82037 | | | EPI289117 | |  |  |  |  |
| A/canine/NY/dog1c02/2009 | EPI_ISL_82031 | | | EPI289111 | |  |  |  |  |
| A/canine/NY/dog1c04/2009 | EPI_ISL_82029 | | | EPI289109 | |  |  |  |  |
| A/canine/NY/dog1c05/2009 | EPI_ISL_82036 | | | EPI289116 | |  |  |  |  |
| A/canine/NY/dog1c06/2009 | EPI_ISL_82030 | | | EPI289110 | |  |  |  |  |
| A/canine/NY/dog1c07/2009 | EPI_ISL_82034 | | | EPI289114 | |  |  |  |  |
| A/canine/NY/dog1c08/2009 | EPI_ISL_82038 | | | EPI289118 | |  |  |  |  |
| A/canine/NY/dog1c09/2009 | EPI_ISL_82032 | | | EPI289112 | |  |  |  |  |
| A/canine/NY/dog1c10/2009 | EPI_ISL_82033 | | | EPI289113 | |  |  |  |  |
| A/canine/NY/dog1c11/2009 | EPI_ISL_82022 | | | EPI289102 | |  |  |  |  |
| A/canine/NY/dog1c12/2009 | EPI_ISL_82023 | | | EPI289103 | |  |  |  |  |
| A/canine/NY/dog1c13/2009 | EPI_ISL_82035 | | | EPI289115 | |  |  |  |  |
| A/canine/NY/dog1c14/2009 | EPI_ISL_82024 | | | EPI289104 | |  |  |  |  |
| A/canine/NY/dog1c15/2009 | EPI_ISL_82025 | | | EPI289105 | |  |  |  |  |
| A/canine/NY/dog1c16/2009 | EPI_ISL_82054 | | | EPI289134 | |  |  |  |  |
| A/canine/NY/dog1c17/2009 | EPI_ISL_82026 | | | EPI289106 | |  |  |  |  |
| A/canine/NY/dog1c18/2009 | EPI_ISL_82027 | | | EPI289107 | |  |  |  |  |
| A/canine/NY/dog1c19/2009 | EPI_ISL_82028 | | | EPI289108 | |  |  |  |  |
| A/canine/NY/dog21c07/2009 | EPI_ISL_81928 | | | EPI289008 | |  |  |  |  |
| A/canine/NY/dog21c08/2009 | EPI_ISL_81929 | | | EPI289009 | |  |  |  |  |
| A/canine/NY/dog21c14/2009 | EPI_ISL_81922 | | | EPI289002 | |  |  |  |  |
| A/canine/NY/dog21c15/2009 | EPI_ISL_81923 | | | EPI289003 | |  |  |  |  |
| A/canine/NY/dog21c16/2009 | EPI_ISL_81924 | | | EPI289004 | |  |  |  |  |
| A/canine/NY/dog21c19/2009 | EPI_ISL_81925 | | | EPI289005 | |  |  |  |  |
| A/canine/NY/dog22c12/2009 | EPI_ISL_81932 | | | EPI289012 | |  |  |  |  |
| A/canine/NY/dog22c20/2009 | EPI_ISL_81931 | | | EPI289011 | |  |  |  |  |
| A/canine/NY/dog23c04/2009 | EPI_ISL_81930 | | | EPI289010 | |  |  |  |  |
| A/canine/NY/dog23c07/2009 | EPI_ISL_81927 | | | EPI289007 | |  |  |  |  |
| A/canine/NY/dog23c19/2009 | EPI_ISL_81926 | | | EPI289006 | |  |  |  |  |
| A/canine/NY/dog2c01/2009 | EPI_ISL_82042 | | | EPI289122 | |  |  |  |  |
| A/canine/NY/dog2c02/2009 | EPI_ISL_82041 | | | EPI289121 | |  |  |  |  |
| A/canine/NY/dog2c04/2009 | EPI_ISL_82040 | | | EPI289120 | |  |  |  |  |
| A/canine/NY/dog2c05/2009 | EPI_ISL_82039 | | | EPI289119 | |  |  |  |  |
| A/canine/NY/dog2c06/2009 | EPI_ISL_82046 | | | EPI289126 | |  |  |  |  |
| A/canine/NY/dog2c09/2009 | EPI_ISL_82044 | | | EPI289124 | |  |  |  |  |
| A/canine/NY/dog2c10/2009 | EPI_ISL_82049 | | | EPI289129 | |  |  |  |  |
| A/canine/NY/dog2c11/2009 | EPI_ISL_82048 | | | EPI289128 | |  |  |  |  |
| A/canine/NY/dog2c13/2009 | EPI_ISL_82050 | | | EPI289130 | |  |  |  |  |
| A/canine/NY/dog2c14/2009 | EPI_ISL_82047 | | | EPI289127 | |  |  |  |  |
| A/canine/NY/dog2c17/2009 | EPI_ISL_82043 | | | EPI289123 | |  |  |  |  |
| A/canine/NY/dog2c20/2009 | EPI_ISL_82045 | | | EPI289125 | |  |  |  |  |
| A/canine/NY/dog3c04/2009 | EPI_ISL_82051 | | | EPI289131 | |  |  |  |  |
| A/canine/NY/dog3c09/2009 | EPI_ISL_82056 | | | EPI289136 | |  |  |  |  |
| A/canine/NY/dog3c11/2009 | EPI_ISL_82055 | | | EPI289135 | |  |  |  |  |
| A/canine/NY/dog3c12/2009 | EPI_ISL_82014 | | | EPI289094 | |  |  |  |  |
| A/canine/NY/dog3c19/2009 | EPI_ISL_82015 | | | EPI289095 | |  |  |  |  |
| A/canine/NY/dog3c20/2009 | EPI_ISL_82016 | | | EPI289096 | |  |  |  |  |
| A/canine/NY/dog3c24/2009 | EPI_ISL_81953 | | | EPI289033 | |  |  |  |  |
| A/canine/NY/dog3c26/2009 | EPI_ISL_81952 | | | EPI289032 | |  |  |  |  |
| A/canine/NY/dog3c28/2009 | EPI_ISL_81939 | | | EPI289019 | |  |  |  |  |
| A/canine/NY/dog3c29/2009 | EPI_ISL_81940 | | | EPI289020 | |  |  |  |  |
| A/canine/NY/dog3c30/2009 | EPI_ISL_81941 | | | EPI289021 | |  |  |  |  |
| A/canine/NY/dog3c31/2009 | EPI_ISL_81942 | | | EPI289022 | |  |  |  |  |
| A/canine/NY/dog3c32/2009 | EPI_ISL_81946 | | | EPI289026 | |  |  |  |  |
| A/canine/NY/dog3c37/2009 | EPI_ISL_81947 | | | EPI289027 | |  |  |  |  |
| A/canine/NY/dog3c40/2009 | EPI_ISL_81943 | | | EPI289023 | |  |  |  |  |
| A/canine/NY/dog4c01/2009 | EPI_ISL_82013 | | | EPI289093 | |  |  |  |  |
| A/canine/NY/dog4c02/2009 | EPI_ISL_82011 | | | EPI289091 | |  |  |  |  |
| A/canine/NY/dog4c03/2009 | EPI_ISL_82010 | | | EPI289090 | |  |  |  |  |
| A/canine/NY/dog4c06/2009 | EPI_ISL_82012 | | | EPI289092 | |  |  |  |  |
| A/canine/NY/dog4c08/2009 | EPI_ISL_82007 | | | EPI289087 | |  |  |  |  |
| A/canine/NY/dog4c12/2009 | EPI_ISL_82000 | | | EPI289080 | |  |  |  |  |
| A/canine/NY/dog4c13/2009 | EPI_ISL_82009 | | | EPI289089 | |  |  |  |  |
| A/canine/NY/dog4c15/2009 | EPI_ISL_82003 | | | EPI289083 | |  |  |  |  |
| A/canine/NY/dog4c16/2009 | EPI_ISL_82002 | | | EPI289082 | |  |  |  |  |
| A/canine/NY/dog4c17/2009 | EPI_ISL_82004 | | | EPI289084 | |  |  |  |  |
| A/canine/NY/dog4c20/2009 | EPI_ISL_82005 | | | EPI289085 | |  |  |  |  |
| A/canine/NY/dog5c03/2009 | EPI_ISL_82052 | | | EPI289132 | |  |  |  |  |
| A/canine/NY/dog5c05/2009 | EPI_ISL_82053 | | | EPI289133 | |  |  |  |  |
| A/canine/NY/dog5c06/2009 | EPI_ISL_82019 | | | EPI289099 | |  |  |  |  |
| A/canine/NY/dog5c07/2009 | EPI_ISL_82018 | | | EPI289098 | |  |  |  |  |
| A/canine/NY/dog5c10/2009 | EPI_ISL_82017 | | | EPI289097 | |  |  |  |  |
| A/canine/NY/dog5c11/2009 | EPI_ISL_82020 | | | EPI289100 | |  |  |  |  |
| A/canine/NY/dog5c19/2009 | EPI_ISL_82021 | | | EPI289101 | |  |  |  |  |
| A/canine/NY/dog5c22/2009 | EPI_ISL_81948 | | | EPI289028 | |  |  |  |  |
| A/canine/NY/dog5c24/2009 | EPI_ISL_81949 | | | EPI289029 | |  |  |  |  |
| A/canine/NY/dog5c26/2009 | EPI_ISL_81950 | | | EPI289030 | |  |  |  |  |
| A/canine/NY/dog5c27/2009 | EPI_ISL_81944 | | | EPI289024 | |  |  |  |  |
| A/canine/NY/dog5c29/2009 | EPI_ISL_81945 | | | EPI289025 | |  |  |  |  |
| A/canine/NY/dog5c34/2009 | EPI_ISL_81951 | | | EPI289031 | |  |  |  |  |
| A/canine/NY/dog5c35/2009 | EPI_ISL_81933 | | | EPI289013 | |  |  |  |  |
| A/canine/NY/dog5c36/2009 | EPI_ISL_81938 | | | EPI289018 | |  |  |  |  |
| A/canine/NY/dog5c39/2009 | EPI_ISL_81934 | | | EPI289014 | |  |  |  |  |
| A/canine/NY/dog6c03/2009 | EPI_ISL_82008 | | | EPI289088 | |  |  |  |  |
| A/canine/NY/dog6c04/2009 | EPI_ISL_81998 | | | EPI289078 | |  |  |  |  |
| A/canine/NY/dog6c05/2009 | EPI_ISL_81999 | | | EPI289079 | |  |  |  |  |
| A/canine/NY/dog6c07/2009 | EPI_ISL_82001 | | | EPI289081 | |  |  |  |  |
| A/canine/NY/dog6c17/2009 | EPI_ISL_81997 | | | EPI289077 | |  |  |  |  |
| A/canine/NY/dog6c19/2009 | EPI_ISL_82006 | | | EPI289086 | |  |  |  |  |
| A/canine/NY/dog6c22/2009 | EPI_ISL_81935 | | | EPI289015 | |  |  |  |  |
| A/canine/NY/dog6c31/2009 | EPI_ISL_81936 | | | EPI289016 | |  |  |  |  |
| A/canine/NY/dog6c34/2009 | EPI_ISL_81937 | | | EPI289017 | |  |  |  |  |
| A/canine/NY/dog7c01/2008 | EPI_ISL_81985 | | | EPI289065 | |  |  |  |  |
| A/canine/NY/dog7c02/2008 | EPI_ISL_81984 | | | EPI289064 | |  |  |  |  |
| A/canine/NY/dog7c03/2008 | EPI_ISL_81954 | | | EPI289034 | |  |  |  |  |
| A/canine/NY/dog7c04/2008 | EPI_ISL_81955 | | | EPI289035 | |  |  |  |  |
| A/canine/NY/dog7c05/2008 | EPI_ISL_81986 | | | EPI289066 | |  |  |  |  |
| A/canine/NY/dog7c06/2008 | EPI_ISL_81957 | | | EPI289037 | |  |  |  |  |
| A/canine/NY/dog7c07/2008 | EPI_ISL_81956 | | | EPI289036 | |  |  |  |  |
| A/canine/NY/dog7c09/2008 | EPI_ISL_81987 | | | EPI289067 | |  |  |  |  |
| A/canine/NY/dog7c10/2008 | EPI_ISL_81983 | | | EPI289063 | |  |  |  |  |
| A/canine/NY/dog7c11/2008 | EPI_ISL_81988 | | | EPI289068 | |  |  |  |  |
| A/canine/NY/dog7c12/2008 | EPI_ISL_81989 | | | EPI289069 | |  |  |  |  |
| A/canine/NY/dog7c13/2008 | EPI_ISL_81990 | | | EPI289070 | |  |  |  |  |
| A/canine/NY/dog7c14/2008 | EPI_ISL_81991 | | | EPI289071 | |  |  |  |  |
| A/canine/NY/dog7c15/2008 | EPI_ISL_81992 | | | EPI289072 | |  |  |  |  |
| A/canine/NY/dog7c16/2008 | EPI_ISL_81993 | | | EPI289073 | |  |  |  |  |
| A/canine/NY/dog7c17/2008 | EPI_ISL_81994 | | | EPI289074 | |  |  |  |  |
| A/canine/NY/dog7c19/2008 | EPI_ISL_81995 | | | EPI289075 | |  |  |  |  |
| A/canine/NY/dog7c20/2008 | EPI_ISL_81996 | | | EPI289076 | |  |  |  |  |
| A/canine/NY/dog8c01/2008 | EPI_ISL_81969 | | | EPI289049 | |  |  |  |  |
| A/canine/NY/dog8c02/2008 | EPI_ISL_81970 | | | EPI289050 | |  |  |  |  |
| A/canine/NY/dog8c03/2008 | EPI_ISL_81971 | | | EPI289051 | |  |  |  |  |
| A/canine/NY/dog8c05/2008 | EPI_ISL_81981 | | | EPI289061 | |  |  |  |  |
| A/canine/NY/dog8c06/2008 | EPI_ISL_81972 | | | EPI289052 | |  |  |  |  |
| A/canine/NY/dog8c07/2008 | EPI_ISL_81973 | | | EPI289053 | |  |  |  |  |
| A/canine/NY/dog8c09/2008 | EPI_ISL_81974 | | | EPI289054 | |  |  |  |  |
| A/canine/NY/dog8c10/2008 | EPI_ISL_81975 | | | EPI289055 | |  |  |  |  |
| A/canine/NY/dog8c12/2008 | EPI_ISL_81982 | | | EPI289062 | |  |  |  |  |
| A/canine/NY/dog8c13/2008 | EPI_ISL_81976 | | | EPI289056 | |  |  |  |  |
| A/canine/NY/dog8c14/2008 | EPI_ISL_81977 | | | EPI289057 | |  |  |  |  |
| A/canine/NY/dog8c15/2008 | EPI_ISL_81978 | | | EPI289058 | |  |  |  |  |
| A/canine/NY/dog8c16/2008 | EPI_ISL_81980 | | | EPI289060 | |  |  |  |  |
| A/canine/NY/dog8c20/2008 | EPI_ISL_81979 | | | EPI289059 | |  |  |  |  |
| A/canine/NY/dog9c03/2008 | EPI_ISL_81960 | | | EPI289040 | |  |  |  |  |
| A/canine/NY/dog9c04/2008 | EPI_ISL_81958 | | | EPI289038 | |  |  |  |  |
| A/canine/NY/dog9c05/2008 | EPI_ISL_81959 | | | EPI289039 | |  |  |  |  |
| A/canine/NY/dog9c07/2008 | EPI_ISL_81961 | | | EPI289041 | |  |  |  |  |
| A/canine/NY/dog9c08/2008 | EPI_ISL_81962 | | | EPI289042 | |  |  |  |  |
| A/canine/NY/dog9c09/2008 | EPI_ISL_81963 | | | EPI289043 | |  |  |  |  |
| A/canine/NY/dog9c12/2008 | EPI_ISL_81964 | | | EPI289044 | |  |  |  |  |
| A/canine/NY/dog9c15/2008 | EPI_ISL_81965 | | | EPI289045 | |  |  |  |  |
| A/canine/NY/dog9c16/2008 | EPI_ISL_81966 | | | EPI289046 | |  |  |  |  |
| A/canine/NY/dog9c18/2008 | EPI_ISL_81967 | | | EPI289047 | |  |  |  |  |
| A/canine/NY/dog9c20/2008 | EPI_ISL_81968 | | | EPI289048 | |  |  |  |  |
| A/canine/PA/111788.2/2009 | EPI_ISL_166506 | | | EPI546839 | |  |  |  |  |
| A/canine/PA/27637.3/2010 | EPI_ISL_167697 | | | EPI546832 | |  |  |  |  |
| A/canine/PA/33225.4/2010 | EPI_ISL_167696 | | | EPI546830 | |  |  |  |  |
| A/canine/PA/96978/2009 | EPI_ISL_167699 | | | EPI546834 | |  |  |  |  |
| A/canine/Pennsylvania/10909/2007 | EPI_ISL_79891 | | | EPI281534 | |  |  |  |  |
| A/canine/Pennsylvania/10915/2007 | EPI_ISL_79892 | | | EPI281542 | |  |  |  |  |
| A/canine/Pennsylvania/137154/2008 | EPI_ISL_79903 | | | EPI281630 | |  |  |  |  |
| A/canine/Pennsylvania/16699/2007 | EPI_ISL_79890 | | | EPI281526 | |  |  |  |  |
| A/canine/Pennsylvania/94930-3/2007 | EPI_ISL_79897 | | | EPI281582 | |  |  |  |  |
| A/canine/Pennsylvania/96978/2009 | EPI_ISL_294937 | | | EPI1157889 | |  |  |  |  |
| A/canine/Philadelphia/6371100/2008 | EPI_ISL_82057 | | | EPI289860 | |  |  |  |  |
| A/canine/StatenIs/115719/2007 | EPI_ISL_81921 | | | EPI289001 | |  |  |  |  |
| A/canine/Sydney/6525/2007 | EPI_ISL_63432 | | | EPI221709 | |  |  |  |  |
| A/canine/Sydney/6692/2007 | EPI_ISL_63433 | | | EPI221712 | |  |  |  |  |
| A/canine/Texas/1/2004 | EPI_ISL_9535 | | | EPI98570 | |  |  |  |  |
| A/canine/Vermont/278213/2013 | EPI_ISL_294941 | | | EPI1157816 | |  |  |  |  |
| A/canine/Virginia/93653/2009 | EPI_ISL_82078 | | | EPI289861 | |  |  |  |  |
| A/canine/VT/11039/2013 | EPI_ISL_166505 | | | EPI546831 | |  |  |  |  |
| A/canine/VT/2782/2013 | EPI_ISL_167701 | | | EPI546836 | |  |  |  |  |
| A/donkey/Shandong/1/2017 | EPI_ISL_284681 | | | EPI1098483 | |  |  |  |  |
| A/eq/Hong Kong/1/92 | EPI_ISL_683 | | | EPI3614 | |  |  |  |  |
| A/eq/Kentucky/92 | EPI_ISL_68829 | | | EPI239790 | |  |  |  |  |
| A/eq/LaPlata/1/88 | EPI_ISL_678 | | | EPI3599 | |  |  |  |  |
| A/eq/LaPlata/93 | EPI_ISL_68814 | | | EPI239729 | |  |  |  |  |
| A/eq/Miami/63 | EPI_ISL_69891 | | | EPI242343 | |  |  |  |  |
| A/eq/Roma/5/91 | EPI_ISL_682 | | | EPI3611 | |  |  |  |  |
| A/Equine/Alaska/1/91 | EPI_ISL_680 | | | EPI3605 | |  |  |  |  |
| A/equine/Alaska/29759/1991 | EPI_ISL_15351 | | | EPI133220 | |  |  |  |  |
| A/equine/Algiers/1/1972 | EPI_ISL_20808 | | | EPI156250 | |  |  |  |  |
| A/equine/Almaty/26/2007 | EPI_ISL_71977 | | | EPI250110 | |  |  |  |  |
| A/equine/Ankara/1/2013 | EPI_ISL_281349 | | | EPI1076909 | |  |  |  |  |
| A/equine/Arundel/12369/91 | EPI_ISL_681 | | | EPI3608 | |  |  |  |  |
| A/equine/Austria/421/1992 | EPI_ISL_19675 | | | EPI153955 | |  |  |  |  |
| A/Equine/Ayrshire/1/2013 | EPI_ISL_240595 | | | EPI873574 | |  |  |  |  |
| A/equine/Baizak/09/2012 | EPI_ISL_170787 | | | EPI558699 | |  |  |  |  |
| A/equine/Belfond/6-2/2009 | EPI_ISL_257677 | | | EPI969059 | |  |  |  |  |
| A/equine/Berlin/1/1989 | EPI_ISL_19683 | | | EPI154019 | |  |  |  |  |
| A/equine/Brno/1/1989 | EPI_ISL_68864 | | | EPI239907 | |  |  |  |  |
| A/equine/Brno/1/1995 | EPI_ISL_68697 | | | EPI239312 | |  |  |  |  |
| A/equine/Brno/2/1989 | EPI_ISL_68865 | | | EPI239908 | |  |  |  |  |
| A/equine/Brno/2-P1/1995 | EPI_ISL_68698 | | | EPI239313 | |  |  |  |  |
| A/equine/Brno/3/1989 | EPI_ISL_68866 | | | EPI239909 | |  |  |  |  |
| A/equine/Buckinghamshire/1/2014 | EPI_ISL_197753 | | | EPI651401 | |  |  |  |  |
| A/equine/California/1/10 | EPI_ISL_177498 | | | EPI584273 | |  |  |  |  |
| A/equine/California/1/1980 | EPI_ISL_8089 | | | EPI77750 | |  |  |  |  |
| A/equine/California/103/1982 | EPI_ISL_8101 | | | EPI77978 | |  |  |  |  |
| A/equine/California/4537/1997 | EPI_ISL_15842 | | | EPI137758 | |  |  |  |  |
| A/equine/California/83/1982 | EPI_ISL_15343 | | | EPI133049 | |  |  |  |  |
| A/equine/California/8560/2002 | EPI_ISL_15843 | | | EPI137777 | |  |  |  |  |
| A/equine/Cambremer/1/2012 | EPI_ISL_257680 | | | EPI969060 | |  |  |  |  |
| A/equine/Cheshire/06 | EPI_ISL_177472 | | | EPI584152 | |  |  |  |  |
| A/equine/Cordoba/18/1985 | EPI_ISL_19671 | | | EPI153923 | |  |  |  |  |
| A/equine/Czech Republic/10076-2/2009 | EPI_ISL_60936 | | | EPI211767 | |  |  |  |  |
| A/equine/Detroit/2/1963 | EPI_ISL_69226 | | | EPI240810 | |  |  |  |  |
| A/equine/Devon/1/11 | EPI_ISL_156749 | | | EPI584247 | |  |  |  |  |
| A/equine/Dorset/09 | EPI_ISL_177491 | | | EPI584206 | |  |  |  |  |
| A/equine/Dubai/1/12 | EPI_ISL_177501 | | | EPI584289 | |  |  |  |  |
| A/equine/East Renfrewshire/2/11 | EPI_ISL_156750 | | | EPI584239 | |  |  |  |  |
| A/Equine/East Sussex/1/2015 | EPI_ISL_240662 | | | EPI873583 | |  |  |  |  |
| A/Equine/Fife/1/16 | EPI_ISL_255632 | | | EPI957624 | |  |  |  |  |
| A/equine/FL/146609/2011 | EPI_ISL_167702 | | | EPI546837 | |  |  |  |  |
| A/equine/Florida/146609/2011 | EPI_ISL_294944 | | | EPI1157862 | |  |  |  |  |
| A/equine/Fontainbleu/1/1979 | EPI_ISL_19682 | | | EPI154011 | |  |  |  |  |
| A/equine/France/1/1967 | EPI_ISL_22612 | | | EPI159281 | |  |  |  |  |
| A/equine/Gansu/7/2008 | EPI_ISL_32656 | | | EPI186857 | |  |  |  |  |
| A/equine/Georgia/1/1981 | EPI_ISL_8095 | | | EPI77864 | |  |  |  |  |
| A/equine/Georgia/10/1981 | EPI_ISL_8098 | | | EPI77921 | |  |  |  |  |
| A/equine/Georgia/121362-16/2016 | EPI_ISL_294939 | | | EPI1157819 | |  |  |  |  |
| A/equine/Georgia/13/1981 | EPI_ISL_8099 | | | EPI77940 | |  |  |  |  |
| A/equine/Georgia/3/1981 | EPI_ISL_8096 | | | EPI77883 | |  |  |  |  |
| A/equine/Georgia/9/1981 | EPI_ISL_8097 | | | EPI77902 | |  |  |  |  |
| A/equine/Gironde/1/2014 | EPI_ISL_257682 | | | EPI969061 | |  |  |  |  |
| A/Equine/Gloucestershire/1/16 | EPI_ISL_255667 | | | EPI957673 | |  |  |  |  |
| A/Equine/Gloucestershire/2/16 | EPI_ISL_255686 | | | EPI957815 | |  |  |  |  |
| A/Equine/Gloucestershire/3/16 | EPI_ISL_255687 | | | EPI957818 | |  |  |  |  |
| A/equine/Gopeswar/1/2009 | EPI_ISL_74365 | | | EPI255776 | |  |  |  |  |
| A/equine/Guangxi/1/2008 | EPI_ISL_174545 | | | EPI573755 | |  |  |  |  |
| A/equine/Hampshire/3/2016 | EPI_ISL_231828 | | | EPI957612 | |  |  |  |  |
| A/Equine/Hampshire/4/2016 | EPI_ISL_240664 | | | EPI873599 | |  |  |  |  |
| A/equine/Heilongjiang/1/2010 | EPI_ISL_144923 | | | EPI464985 | |  |  |  |  |
| A/equine/Heilongjiang/10/2008 | EPI_ISL_32658 | | | EPI186873 | |  |  |  |  |
| A/equine/Heilongjiang/SS1/2013 | EPI_ISL_161474 | | | EPI527594 | |  |  |  |  |
| A/equine/Himachal Pradesh/CMVL-YOL2/2008 | EPI_ISL_190904 | | | EPI621746 | |  |  |  |  |
| A/equine/Hissar/CMVL-HSR4/2008 | EPI_ISL_190901 | | | EPI621727 | |  |  |  |  |
| A/equine/Hokkaido/I828/2008 | EPI_ISL_69119 | | | EPI240685 | |  |  |  |  |
| A/equine/Hong Kong/J/1992 | EPI_ISL_68821 | | | EPI239761 | |  |  |  |  |
| A/equine/Huabei/1/2007 | EPI_ISL_70195 | | | EPI243550 | |  |  |  |  |
| A/equine/Hubei/6/2008 | EPI_ISL_32657 | | | EPI186865 | |  |  |  |  |
| A/equine/Ibaraki/1/2007 | EPI_ISL_297956 | | | EPI1172965 | |  |  |  |  |
| A/equine/Idaho/37875/1991 | EPI_ISL_6623 | | | EPI52069 | |  |  |  |  |
| A/equine/Inner Mongolia/8/2008 | EPI_ISL_32660 | | | EPI186889 | |  |  |  |  |
| A/equine/Italy/1062/1991 | EPI_ISL_19678 | | | EPI153979 | |  |  |  |  |
| A/equine/Italy/1199/1992 | EPI_ISL_19674 | | | EPI153947 | |  |  |  |  |
| A/equine/Italy/788/1/1991 | EPI_ISL_68838 | | | EPI239828 | |  |  |  |  |
| A/equine/Italy/824/1991 | EPI_ISL_19673 | | | EPI153939 | |  |  |  |  |
| A/equine/Jammu and Kashmir/CMVL-LEH4/2008 | EPI_ISL_190902 | | | EPI621733 | |  |  |  |  |
| A/equine/Jammu and Kashmir/CMVL-LEH6/2008 | EPI_ISL_190903 | | | EPI621739 | |  |  |  |  |
| A/equine/Johannesburg/1/1986 | EPI_ISL_20809 | | | EPI156258 | |  |  |  |  |
| A/equine/Jouars/4/2006 | EPI_ISL_257684 | | | EPI969058 | |  |  |  |  |
| A/equine/Kanazawa/1/2007 | EPI_ISL_666 | | | EPI3528 | |  |  |  |  |
| A/equine/Kascakew/1/1978 | EPI_ISL_69898 | | | EPI242356 | |  |  |  |  |
| A/equine/Katra-Jammu/7/2008 | EPI_ISL_74366 | | | EPI300770 | |  |  |  |  |
| A/Equine/Kent/1/15 | EPI_ISL_240663 | | | EPI873591 | |  |  |  |  |
| A/Equine/Kent/1/16 | EPI_ISL_255630 | | | EPI957610 | |  |  |  |  |
| A/equine/Kentucky/1/11 | EPI_ISL_177500 | | | EPI584281 | |  |  |  |  |
| A/equine/Kentucky/1/1978 | EPI_ISL_8087 | | | EPI77712 | |  |  |  |  |
| A/equine/Kentucky/1/1981 | EPI_ISL_68957 | | | EPI240111 | |  |  |  |  |
| A/equine/Kentucky/1/1986 | EPI_ISL_15344 | | | EPI133068 | |  |  |  |  |
| A/equine/Kentucky/1/1987 | EPI_ISL_21060 | | | EPI133087 | |  |  |  |  |
| A/equine/Kentucky/1/1991 | EPI_ISL_15352 | | | EPI133258 | |  |  |  |  |
| A/equine/Kentucky/1/1992 | EPI_ISL_15350 | | | EPI133201 | |  |  |  |  |
| A/equine/Kentucky/1/81 | EPI_ISL_689 | | | EPI3596 | |  |  |  |  |
| A/equine/Kentucky/1277/1990 | EPI_ISL_15348 | | | EPI133163 | |  |  |  |  |
| A/equine/Kentucky/2/1980 | EPI_ISL_20807 | | | EPI156242 | |  |  |  |  |
| A/equine/Kentucky/2/1981 | EPI_ISL_8090 | | | EPI77769 | |  |  |  |  |
| A/equine/Kentucky/2/1986 | EPI_ISL_14705 | | | EPI130198 | |  |  |  |  |
| A/equine/Kentucky/2/1986 | EPI_ISL_14705 | | | EPI130198 | |  |  |  |  |
| A/equine/Kentucky/2/1987 | EPI_ISL_19104 | | | EPI152770 | |  |  |  |  |
| A/equine/Kentucky/211/1987 | EPI_ISL_68958 | | | EPI240119 | |  |  |  |  |
| A/equine/Kentucky/3/1981 | EPI_ISL_15342 | | | EPI133030 | |  |  |  |  |
| A/equine/Kentucky/3/1986 | EPI_ISL_19663 | | | EPI153851 | |  |  |  |  |
| A/equine/Kentucky/4/1980 | EPI_ISL_8088 | | | EPI77731 | |  |  |  |  |
| A/equine/Kentucky/5/02 | EPI_ISL_4663 | | | EPI25748 | |  |  |  |  |
| A/equine/Kentucky/692/1988 | EPI_ISL_15345 | | | EPI133106 | |  |  |  |  |
| A/equine/Kentucky/694/1988 | EPI_ISL_15346 | | | EPI133125 | |  |  |  |  |
| A/equine/Kentucky/698/1988 | EPI_ISL_15347 | | | EPI133144 | |  |  |  |  |
| A/equine/Kentucky/8/1994 | EPI_ISL_15353 | | | EPI133277 | |  |  |  |  |
| A/equine/Kentucky/bitter_boredom5/1976 | EPI_ISL_8094 | | | EPI77845 | |  |  |  |  |
| A/equine/Kentucky/magnificent_genius1/1981 | EPI_ISL_8100 | | | EPI77959 | |  |  |  |  |
| A/equine/Kentucky/pass_the_pepper1/1976 | EPI_ISL_8093 | | | EPI77826 | |  |  |  |  |
| A/equine/Kentucky/Rosie100/1981 | EPI_ISL_15830 | | | EPI137530 | |  |  |  |  |
| A/equine/Kostanay/09/2012 | EPI_ISL_170790 | | | EPI558702 | |  |  |  |  |
| A/equine/Kyonggi/SA1/2011 | EPI_ISL_129971 | | | EPI398890 | |  |  |  |  |
| A/equine/Lambourn/22778/92 | EPI_ISL_685 | | | EPI3620 | |  |  |  |  |
| A/equine/Lanarkshire/09 | EPI_ISL_177490 | | | EPI584198 | |  |  |  |  |
| A/Equine/Lancashire/1/16 | EPI_ISL_255635 | | | EPI957671 | |  |  |  |  |
| A/equine/Liaoning/9/2008 | EPI_ISL_32659 | | | EPI186881 | |  |  |  |  |
| A/equine/Lincolnshire/06 | EPI_ISL_177471 | | | EPI584130 | |  |  |  |  |
| A/equine/Lincolnshire/1/2007 | EPI_ISL_29831 | | | EPI501406 | |  |  |  |  |
| A/equine/LKZ/09/2012 | EPI_ISL_170788 | | | EPI558700 | |  |  |  |  |
| A/equine/Malaysia/M201/2015 | EPI_ISL_201434 | | | EPI1073181 | |  |  |  |  |
| A/equine/Massachussetts/213/2003 | EPI_ISL_9529 | | | EPI98495 | |  |  |  |  |
| A/equine/Matybulak/10/2012 | EPI_ISL_170789 | | | EPI558701 | |  |  |  |  |
| A/equine/Miami/1/1963 | EPI_ISL_8092 | | | EPI77807 | |  |  |  |  |
| A/equine/Miami/1963 | EPI_ISL_143221 | | | EPI459664 | |  |  |  |  |
| A/equine/Mongolia/1/2008 | EPI_ISL_19180 | | | EPI153116 | |  |  |  |  |
| A/equine/Mongolia/3/2011 | EPI_ISL_129371 | | | EPI395968 | |  |  |  |  |
| A/equine/Mongolia/56/2011 | EPI_ISL_129373 | | | EPI395972 | |  |  |  |  |
| A/equine/Montana/9233/2007 | EPI_ISL_79893 | | | EPI281550 | |  |  |  |  |
| A/equine/Montana/9564-1/2015 | EPI_ISL_285417 | | | EPI1103411 | |  |  |  |  |
| A/equine/Moulton/1/1998 | EPI_ISL_91607 | | | EPI342597 | |  |  |  |  |
| A/equine/Moulton/98 | EPI_ISL_177487 | | | EPI584182 | |  |  |  |  |
| A/equine/Mysore/12/2008 | EPI_ISL_84310 | | | EPI296142 | |  |  |  |  |
| A/equine/Neuville-Pres-Sees/1/2011 | EPI_ISL_257679 | | | EPI969063 | |  |  |  |  |
| A/equine/New Market/nasalwash1/1979 | EPI_ISL_95198 | | | EPI332925 | |  |  |  |  |
| A/equine/New York/1/1983 | EPI_ISL_15356 | | | EPI133334 | |  |  |  |  |
| A/equine/New York/135857/2016 | EPI_ISL_294936 | | | EPI1157893 | |  |  |  |  |
| A/equine/New York/146066/2007 | EPI_ISL_79900 | | | EPI281606 | |  |  |  |  |
| A/equine/New York/VR-297/1983 | EPI_ISL_8102 | | | EPI77997 | |  |  |  |  |
| A/equine/Newmarket/1/1993 | EPI_ISL_30895 | | | EPI239738 | |  |  |  |  |
| A/equine/Newmarket/11/2003 | EPI_ISL_98910 | | | EPI341901 | |  |  |  |  |
| A/equine/Newmarket/1979 | EPI_ISL_143220 | | | EPI459671 | |  |  |  |  |
| A/equine/Newmarket/2/1993 | EPI_ISL_30896 | | | EPI239744 | |  |  |  |  |
| A/equine/Newmarket/5/2003 | EPI_ISL_30894 | | | EPI235166 | |  |  |  |  |
| A/equine/Northamptonshire/1/2013 | EPI_ISL_151841 | | | EPI584254 | |  |  |  |  |
| A/equine/Ohio/1/2003 | EPI_ISL_9534 | | | EPI98551 | |  |  |  |  |
| A/equine/Ohio/113461-1/2005 | EPI_ISL_79869 | | | EPI281358 | |  |  |  |  |
| A/equine/Ohio/113461-2/2005 | EPI_ISL_79866 | | | EPI281334 | |  |  |  |  |
| A/equine/Ohio/113461-2/2005 | EPI_ISL_79866 | | | EPI281334 | |  |  |  |  |
| A/equine/Ohio/113461-3/2005 | EPI_ISL_79870 | | | EPI281366 | |  |  |  |  |
| A/equine/OR/78356/2012 | EPI_ISL_167703 | | | EPI546838 | |  |  |  |  |
| A/equine/Oregon/78356/2012 | EPI_ISL_294934 | | | EPI1157849 | |  |  |  |  |
| A/equine/Otar/764/2007 | EPI_ISL_89156 | | | EPI314111 | |  |  |  |  |
| A/equine/Perthshire/3/09 | EPI_ISL_177492 | | | EPI584214 | |  |  |  |  |
| A/equine/Philippines/2/1997 | EPI_ISL_91615 | | | EPI342598 | |  |  |  |  |
| A/equine/Pulawy/1/2005 | EPI_ISL_64878 | | | EPI986873 | |  |  |  |  |
| A/equine/Pulawy/1/2006 | EPI_ISL_64366 | | | EPI986874 | |  |  |  |  |
| A/equine/Pulawy/1/2008 | EPI_ISL_168012 | | | EPI986875 | |  |  |  |  |
| A/equine/Qinghai/1/1994 | EPI_ISL_32661 | | | EPI186897 | |  |  |  |  |
| A/equine/Richmond/1/2007 | EPI_ISL_153499 | | | EPI501400 | |  |  |  |  |
| A/equine/Rio Grande do Sul/1/12 | EPI_ISL_177502 | | | EPI584298 | |  |  |  |  |
| A/equine/Roma/5/1991 | EPI_ISL_19677 | | | EPI153971 | |  |  |  |  |
| A/equine/Romania/1/1980 | EPI_ISL_19680 | | | EPI153995 | |  |  |  |  |
| A/equine/Sachiyama/1/1971 | EPI_ISL_25008 | | | EPI165402 | |  |  |  |  |
| A/equine/Santa Fe/1/1985 | EPI_ISL_19672 | | | EPI153931 | |  |  |  |  |
| A/equine/Sao Paulo/1.19/2012 | EPI_ISL_279065 | | | EPI1065024 | |  |  |  |  |
| A/equine/Sao Paulo/1/1969 | EPI_ISL_19681 | | | EPI154003 | |  |  |  |  |
| A/equine/Sao Paulo/6/1963 | EPI_ISL_19670 | | | EPI153915 | |  |  |  |  |
| A/equine/Saone-et-Loire/1/2015 | EPI_ISL_257683 | | | EPI969062 | |  |  |  |  |
| A/equine/Shropshire/10 | EPI_ISL_156748 | | | EPI584230 | |  |  |  |  |
| A/equine/Snailwell/98 | EPI_ISL_177489 | | | EPI584190 | |  |  |  |  |
| A/equine/Solihull/1/2007 | EPI_ISL_29847 | | | EPI341900 | |  |  |  |  |
| A/Equine/South Africa/4/03 | EPI_ISL_240665 | | | EPI873606 | |  |  |  |  |
| A/equine/South Kazakhstan/236/2012 | EPI_ISL_166607 | | | EPI542624 | |  |  |  |  |
| A/Equine/Stirlingshire/1/16 | EPI_ISL_255633 | | | EPI957636 | |  |  |  |  |
| A/equine/Suffolk/95839 | EPI_ISL_66551 | | | EPI232434 | |  |  |  |  |
| A/equine/Sussex/1/1989 | EPI_ISL_19884 | | | EPI240134 | |  |  |  |  |
| A/equine/Sussex/1/1989 | EPI_ISL_19884 | | | EPI240134 | |  |  |  |  |
| A/equine/Sussex/93753/89 | EPI_ISL_8280 | | | EPI154461 | |  |  |  |  |
| A/Equine/Sweden/SVA111206SZ0085/VIR165837/2011 | EPI_ISL_180684 | | | EPI594010 | |  |  |  |  |
| A/equine/Switzerland/1118/1979 | EPI_ISL_19679 | | | EPI153987 | |  |  |  |  |
| A/equine/Switzerland/173/1993 | EPI_ISL_19676 | | | EPI153963 | |  |  |  |  |
| A/equine/Switzerland/2225/1979 | EPI_ISL_22611 | | | EPI159273 | |  |  |  |  |
| A/equine/Switzerland/P112/07 | EPI_ISL_177468 | | | EPI584119 | |  |  |  |  |
| A/equine/Sydney/6085/2007 | EPI_ISL_63504 | | | EPI222043 | |  |  |  |  |
| A/equine/Tennessee/27A/2014 | EPI_ISL_220483 | | | EPI753446 | |  |  |  |  |
| A/equine/Tennessee/28A/2014 | EPI_ISL_220480 | | | EPI753428 | |  |  |  |  |
| A/equine/Tennessee/28B/2014 | EPI_ISL_220484 | | | EPI753453 | |  |  |  |  |
| A/equine/Tennessee/29A/2014 | EPI_ISL_220485 | | | EPI753459 | |  |  |  |  |
| A/equine/Tennessee/30A/2014 | EPI_ISL_220482 | | | EPI753463 | |  |  |  |  |
| A/equine/Tennessee/4A/2014 | EPI_ISL_220481 | | | EPI753435 | |  |  |  |  |
| A/equine/Tennessee/5/1986 | EPI_ISL_21061 | | | EPI133239 | |  |  |  |  |
| A/equine/Tennessee/5/1986 | EPI_ISL_21061 | | | EPI133239 | |  |  |  |  |
| A/equine/Texas/117793/2005 | EPI_ISL_79867 | | | EPI281342 | |  |  |  |  |
| A/equine/Texas/117793/2005 | EPI_ISL_79867 | | | EPI281342 | |  |  |  |  |
| A/equine/Texas/39655/1991 | EPI_ISL_15349 | | | EPI133182 | |  |  |  |  |
| A/equine/Tottori/1/07 | EPI_ISL_89221 | | | EPI314455 | |  |  |  |  |
| A/equine/Uruguay/1/1963 | EPI_ISL_21282 | | | EPI154027 | |  |  |  |  |
| A/equine/Virginia/131054-3/2005 | EPI_ISL_79899 | | | EPI281598 | |  |  |  |  |
| A/equine/Wisconsin/1/03 | EPI_ISL_9783 | | | EPI99382 | |  |  |  |  |
| A/equine/Xinjiang/1/2007 | EPI_ISL_32662 | | | EPI186905 | |  |  |  |  |
| A/equine/Xinjiang/2/2007 | EPI_ISL_32663 | | | EPI186913 | |  |  |  |  |
| A/equine/Xinjiang/3/2007 | EPI_ISL_32664 | | | EPI186921 | |  |  |  |  |
| A/equine/Xinjiang/4/2007 | EPI_ISL_32665 | | | EPI186929 | |  |  |  |  |
| A/equine/Xinjiang/5/2007 | EPI_ISL_32666 | | | EPI186937 | |  |  |  |  |
| A/equine/Xuzhou/01/2013 | EPI_ISL_151130 | | | EPI489631 | |  |  |  |  |
| A/equine/Yokohama/aq13/2010 | EPI_ISL_297955 | | | EPI1172957 | |  |  |  |  |
| A/equine/Yokohama/aq19/2009 | EPI_ISL_69743 | | | EPI241776 | |  |  |  |  |
| A/equine/Yorkshire/3/09 | EPI_ISL_156745 | | | EPI584222 | |  |  |  |  |
| A/equine/Yvelines/2136/89 | EPI_ISL_679 | | | EPI3602 | |  |  |  |  |
| A /Equine/Sweden/SVA111128SZ0073/VIR160172/2011 | EPI_ISL_180755 | | | EPI594561 | |  |  |  |  |
| NA | | | | | | |  |  |  |
| Isolate_Name | Isolate_Id | | | Segment_Id | | |  |  |  |
| A/canine/California/70645-4/2006 | EPI_ISL_79878 | | | EPI281429 | | |  |  |  |
| A/canine/Colorado/148902/2006 | EPI_ISL_87459 | | | EPI307323 | | |  |  |  |
| A/canine/Colorado/17864/2006 | EPI_ISL_79875 | | | EPI281405 | | |  |  |  |
| A/canine/Colorado/17864/2006 | EPI_ISL_79875 | | | EPI281405 | | |  |  |  |
| A/canine/Colorado/224986/2006 | EPI_ISL_87458 | | | EPI307322 | | |  |  |  |
| A/canine/Colorado/3/2006 | EPI_ISL_87460 | | | EPI307324 | | |  |  |  |
| A/canine/Colorado/30604/2006 | EPI_ISL_79877 | | | EPI281421 | | |  |  |  |
| A/canine/Colorado/6723-8/2008 | EPI_ISL_79901 | | | EPI281613 | | |  |  |  |
| A/canine/Colorado/6723-8/2008 | EPI_ISL_79901 | | | EPI281613 | | |  |  |  |
| A/canine/Colorado/8880/2006 | EPI_ISL_79874 | | | EPI281397 | | |  |  |  |
| A/canine/Connecticut/85863/2011 | EPI_ISL_294935 | | | EPI1157806 | | |  |  |  |
| A/canine/Florida/15592.1/2004 | EPI_ISL_294945 | | | EPI1157852 | | |  |  |  |
| A/canine/Florida/242/2003 | EPI_ISL_9528 | | | EPI98487 | | |  |  |  |
| A/canine/Florida/43/2004 | EPI_ISL_9527 | | | EPI98469 | | |  |  |  |
| A/canine/Florida/61156-2/2006 | EPI_ISL_79895 | | | EPI281565 | | |  |  |  |
| A/canine/Florida/61156-2/2006 | EPI_ISL_79895 | | | EPI281565 | | |  |  |  |
| A/canine/Florida/78592-2/2006 | EPI_ISL_79879 | | | EPI281437 | | |  |  |  |
| A/canine/Florida/78592-6/2006 | EPI_ISL_79880 | | | EPI281445 | | |  |  |  |
| A/canine/Florida/78592-7/2006 | EPI_ISL_79881 | | | EPI281453 | | |  |  |  |
| A/canine/Florida/89911-2/2006 | EPI_ISL_79882 | | | EPI281461 | | |  |  |  |
| A/canine/Iowa/13628/2005 | EPI_ISL_9541 | | | EPI98704 | | |  |  |  |
| A/canine/Kentucky/118778/2006 | EPI_ISL_79889 | | | EPI281517 | | |  |  |  |
| A/canine/Maine/058124/2016 | EPI_ISL_294942 | | | EPI1157864 | | |  |  |  |
| A/canine/Massachusetts/26810/2016 | EPI_ISL_234612 | | | EPI839121 | | |  |  |  |
| A/canine/New York/100525-1/2006 | EPI_ISL_79883 | | | EPI281469 | | |  |  |  |
| A/canine/New York/100528-1/2006 | EPI_ISL_79884 | | | EPI281477 | | |  |  |  |
| A/canine/New York/100528-5/2006 | EPI_ISL_79885 | | | EPI281485 | | |  |  |  |
| A/canine/New York/100528-6/2006 | EPI_ISL_79886 | | | EPI281493 | | |  |  |  |
| A/canine/New York/115719/2007 | EPI_ISL_79896 | | | EPI281573 | | |  |  |  |
| A/canine/New York/115809/2005 | EPI_ISL_79864 | | | EPI281317 | | |  |  |  |
| A/canine/New York/145353/2008 | EPI_ISL_79904 | | | EPI281637 | | |  |  |  |
| A/canine/New York/147926-3/2006 | EPI_ISL_79887 | | | EPI281501 | | |  |  |  |
| A/canine/New York/147926-5/2006 | EPI_ISL_79888 | | | EPI281509 | | |  |  |  |
| A/canine/New York/158402-1/2008 | EPI_ISL_79905 | | | EPI281645 | | |  |  |  |
| A/canine/New York/159903/2012 | EPI_ISL_294938 | | | EPI1157853 | | |  |  |  |
| A/canine/New York/1623.1/2010 | EPI_ISL_294943 | | | EPI1157874 | | |  |  |  |
| A/canine/New York/3699/2010 | EPI_ISL_294940 | | | EPI1157827 | | |  |  |  |
| A/canine/New York/4986-2/2006 | EPI_ISL_79873 | | | EPI281389 | | |  |  |  |
| A/canine/New York/5183-6/2006 | EPI_ISL_79871 | | | EPI281373 | | |  |  |  |
| A/canine/New York/5183-6/2006 | EPI_ISL_79871 | | | EPI281373 | | |  |  |  |
| A/canine/New York/51854/2008 | EPI_ISL_79902 | | | EPI281621 | | |  |  |  |
| A/canine/NY/105447/2008 | EPI_ISL_174477 | | | EPI576530 | | |  |  |  |
| A/canine/NY/120106.2/2011 | EPI_ISL_166503 | | | EPI546843 | | |  |  |  |
| A/canine/NY/dog3c01/2009 | EPI_ISL_82138 | | | EPI289482 | | |  |  |  |
| A/canine/NY/dog3c02/2009 | EPI_ISL_82176 | | | EPI289483 | | |  |  |  |
| A/canine/NY/dog3c03/2009 | EPI_ISL_82137 | | | EPI289484 | | |  |  |  |
| A/canine/NY/dog3c04/2009 | EPI_ISL_82051 | | | EPI289485 | | |  |  |  |
| A/canine/NY/dog3c06/2009 | EPI_ISL_82140 | | | EPI289492 | | |  |  |  |
| A/canine/NY/dog3c07/2009 | EPI_ISL_82141 | | | EPI289500 | | |  |  |  |
| A/canine/NY/dog3c08/2009 | EPI_ISL_82179 | | | EPI289493 | | |  |  |  |
| A/canine/NY/dog3c11/2009 | EPI_ISL_82055 | | | EPI289497 | | |  |  |  |
| A/canine/NY/dog3c12/2009 | EPI_ISL_82014 | | | EPI289498 | | |  |  |  |
| A/canine/NY/dog3c13/2009 | EPI_ISL_82143 | | | EPI289494 | | |  |  |  |
| A/canine/NY/dog3c14/2009 | EPI_ISL_82147 | | | EPI289501 | | |  |  |  |
| A/canine/NY/dog3c16/2009 | EPI_ISL_82180 | | | EPI289495 | | |  |  |  |
| A/canine/NY/dog3c17/2009 | EPI_ISL_82145 | | | EPI289496 | | |  |  |  |
| A/canine/NY/dog3c19/2009 | EPI_ISL_82015 | | | EPI289499 | | |  |  |  |
| A/canine/NY/dog4c1/2009 | EPI_ISL_82177 | | | EPI289486 | | |  |  |  |
| A/canine/NY/dog4c11/2009 | EPI_ISL_82073 | | | EPI289488 | | |  |  |  |
| A/canine/NY/dog4c14/2009 | EPI_ISL_82133 | | | EPI289489 | | |  |  |  |
| A/canine/NY/dog4c2/2009 | EPI_ISL_82178 | | | EPI289487 | | |  |  |  |
| A/canine/NY/dog4c20/2009 | EPI_ISL_82005 | | | EPI289491 | | |  |  |  |
| A/canine/NY/dog4c7/2009 | EPI_ISL_82181 | | | EPI289502 | | |  |  |  |
| A/canine/NY/dog5c17/2009 | EPI_ISL_82070 | | | EPI289490 | | |  |  |  |
| A/canine/NY/dog7c01/2008 | EPI_ISL_81985 | | | EPI289478 | | |  |  |  |
| A/canine/NY/dog7c04/2008 | EPI_ISL_81955 | | | EPI289479 | | |  |  |  |
| A/canine/NY/dog7c06/2008 | EPI_ISL_81957 | | | EPI289480 | | |  |  |  |
| A/canine/NY/dog7c09/2008 | EPI_ISL_81987 | | | EPI289481 | | |  |  |  |
| A/canine/NY/dog7c13/2008 | EPI_ISL_81990 | | | EPI289471 | | |  |  |  |
| A/canine/NY/dog7c17/2008 | EPI_ISL_81994 | | | EPI289472 | | |  |  |  |
| A/canine/NY/dog7c21/2008 | EPI_ISL_82171 | | | EPI289473 | | |  |  |  |
| A/canine/NY/dog7c23/2008 | EPI_ISL_82172 | | | EPI289474 | | |  |  |  |
| A/canine/NY/dog7c25/2008 | EPI_ISL_82173 | | | EPI289475 | | |  |  |  |
| A/canine/NY/dog7c27/2008 | EPI_ISL_82174 | | | EPI289476 | | |  |  |  |
| A/canine/NY/dog7c29/2008 | EPI_ISL_82175 | | | EPI289477 | | |  |  |  |
| A/canine/PA/111788.2/2009 | EPI_ISL_166506 | | | EPI546846 | | |  |  |  |
| A/canine/PA/33225.4/2010 | EPI_ISL_167696 | | | EPI546844 | | |  |  |  |
| A/canine/Pennsylvania/10909/2007 | EPI_ISL_79891 | | | EPI281533 | | |  |  |  |
| A/canine/Pennsylvania/10915/2007 | EPI_ISL_79892 | | | EPI281541 | | |  |  |  |
| A/canine/Pennsylvania/137154/2008 | EPI_ISL_79903 | | | EPI281629 | | |  |  |  |
| A/canine/Pennsylvania/16699/2007 | EPI_ISL_79890 | | | EPI281525 | | |  |  |  |
| A/canine/Pennsylvania/94930-3/2007 | EPI_ISL_79897 | | | EPI281581 | | |  |  |  |
| A/canine/Pennsylvania/96978/2009 | EPI_ISL_294937 | | | EPI1157814 | | |  |  |  |
| A/canine/Philadelphia/6371100/2008 | EPI_ISL_82057 | | | EPI289470 | | |  |  |  |
| A/canine/Sydney/6525/2007 | EPI_ISL_63432 | | | EPI221708 | | |  |  |  |
| A/canine/Sydney/6692/2007 | EPI_ISL_63433 | | | EPI221711 | | |  |  |  |
| A/canine/Vermont/278213/2013 | EPI_ISL_294941 | | | EPI1157872 | | |  |  |  |
| A/canine/VT/11039/2013 | EPI_ISL_166505 | | | EPI546845 | | |  |  |  |
| A/canine/Wyoming/86033/2007 | EPI_ISL_87461 | | | EPI307325 | | |  |  |  |
| A/donkey/Shandong/1/2017 | EPI_ISL_284681 | | | EPI1098482 | | |  |  |  |
| A/eqine/Shropshire/5/2013 | EPI_ISL_151985 | | | EPI493620 | | |  |  |  |
| A/equine/Aboyne/2005 | EPI_ISL_153490 | | | EPI499170 | | |  |  |  |
| A/Equine/Alaska/1/91 | EPI_ISL_680 | | | EPI128916 | | |  |  |  |
| A/equine/Alaska/29759/1991 | EPI_ISL_15351 | | | EPI133223 | | |  |  |  |
| A/equine/Alfort/1/1967 | EPI_ISL_257685 | | | EPI969030 | | |  |  |  |
| A/equine/Algiers/1/1972 | EPI_ISL_20808 | | | EPI156251 | | |  |  |  |
| A/equine/Algiers/1972 | EPI_ISL_69895 | | | EPI243223 | | |  |  |  |
| A/equine/Almaty/26/2007 | EPI_ISL_71977 | | | EPI383942 | | |  |  |  |
| A/equine/Ankara/1/2013 | EPI_ISL_281349 | | | EPI1076908 | | |  |  |  |
| A/equine/Austria/421/1992 | EPI_ISL_19675 | | | EPI153956 | | |  |  |  |
| A/Equine/Ayrshire/1/2013 | EPI_ISL_151796 | | | EPI497568 | | |  |  |  |
| A/equine/Ayrshire/1/2013 | EPI_ISL_151796 | | | EPI497568 | | |  |  |  |
| A/equine/Ayrshire/2/2013 | EPI_ISL_234614 | | | EPI838697 | | |  |  |  |
| A/equine/Baizak/09/2012 | EPI_ISL_170787 | | | EPI558691 | | |  |  |  |
| A/equine/Belfond/6-2/2009 | EPI_ISL_257677 | | | EPI969054 | | |  |  |  |
| A/equine/Berlin/1/1989 | EPI_ISL_19683 | | | EPI154020 | | |  |  |  |
| A/equine/Blois/1/1983 | EPI_ISL_257663 | | | EPI969036 | | |  |  |  |
| A/equine/Bordeaux/1/1983 | EPI_ISL_257661 | | | EPI969034 | | |  |  |  |
| A/equine/Buckinghamshire/1/2014 | EPI_ISL_197753 | | | EPI651400 | | |  |  |  |
| A/equine/Cagnes-sur-Mer/2/2000 | EPI_ISL_257675 | | | EPI969048 | | |  |  |  |
| A/equine/Cagnes-sur-Mer/2/2011 | EPI_ISL_257678 | | | EPI969050 | | |  |  |  |
| A/equine/California/1/10 | EPI_ISL_177498 | | | EPI584272 | | |  |  |  |
| A/equine/California/1/1980 | EPI_ISL_8089 | | | EPI77753 | | |  |  |  |
| A/equine/California/103/1982 | EPI_ISL_8101 | | | EPI77981 | | |  |  |  |
| A/equine/California/191/2003 | EPI_ISL_9533 | | | EPI98506 | | |  |  |  |
| A/equine/California/4537/1997 | EPI_ISL_15842 | | | EPI137761 | | |  |  |  |
| A/equine/California/83/1982 | EPI_ISL_15343 | | | EPI133052 | | |  |  |  |
| A/equine/California/8560/2002 | EPI_ISL_15843 | | | EPI137780 | | |  |  |  |
| A/equine/Cambremer/1/2012 | EPI_ISL_257680 | | | EPI969055 | | |  |  |  |
| A/equine/Chantilly/1/1975 | EPI_ISL_257659 | | | EPI969032 | | |  |  |  |
| A/equine/Cheshire/06 | EPI_ISL_177472 | | | EPI584138 | | |  |  |  |
| A/equine/Colorado/10/2007 | EPI_ISL_87462 | | | EPI307326 | | |  |  |  |
| A/equine/Cordoba/18/1985 | EPI_ISL_19671 | | | EPI153924 | | |  |  |  |
| A/equine/County Durham/2/2012 | EPI_ISL_153469 | | | EPI499165 | | |  |  |  |
| A/equine/Devon/1/11 | EPI_ISL_156749 | | | EPI584246 | | |  |  |  |
| A/equine/Devon/1/2011 | EPI_ISL_153462 | | | EPI499166 | | |  |  |  |
| A/equine/Dorset/09 | EPI_ISL_177491 | | | EPI584205 | | |  |  |  |
| A/equine/Dorset/2009 | EPI_ISL_153495 | | | EPI499175 | | |  |  |  |
| A/equine/Dubai/1/12 | EPI_ISL_177501 | | | EPI584288 | | |  |  |  |
| A/equine/Dubai/1/2012 | EPI_ISL_153484 | | | EPI499154 | | |  |  |  |
| A/equine/East Renfrewshire/2/11 | EPI_ISL_156750 | | | EPI584238 | | |  |  |  |
| A/equine/East Renfrewshire/2/2011 | EPI_ISL_153461 | | | EPI499153 | | |  |  |  |
| A/Equine/East Sussex/1/2015 | EPI_ISL_201992 | | | EPI673533 | | |  |  |  |
| A/equine/East Sussex/1/2015 | EPI_ISL_201992 | | | EPI673533 | | |  |  |  |
| A/equine/East Yorkshire/1/2013 | EPI_ISL_151847 | | | EPI497575 | | |  |  |  |
| A/equine/Egypt/6066NAMRU3-VSVRI/2008 | EPI_ISL_89153 | | | EPI314106 | | |  |  |  |
| A/Equine/Fife/1/16 | EPI_ISL_255632 | | | EPI957623 | | |  |  |  |
| A/equine/Florida/146609/2011 | EPI_ISL_294944 | | | EPI1157844 | | |  |  |  |
| A/equine/Florida/612/2004 | EPI_ISL_89154 | | | EPI314108 | | |  |  |  |
| A/equine/Florida/779/2004 | EPI_ISL_89155 | | | EPI314109 | | |  |  |  |
| A/equine/Fontainbleu/1/1979 | EPI_ISL_19682 | | | EPI154012 | | |  |  |  |
| A/equine/Fontainebleau/1/1983 | EPI_ISL_257662 | | | EPI969035 | | |  |  |  |
| A/equine/France/1/1967 | EPI_ISL_22612 | | | EPI159282 | | |  |  |  |
| A/equine/France/1/1973 | EPI_ISL_257658 | | | EPI969031 | | |  |  |  |
| A/equine/France/1/1985 | EPI_ISL_257665 | | | EPI969038 | | |  |  |  |
| A/equine/France/1/1986 | EPI_ISL_257668 | | | EPI969041 | | |  |  |  |
| A/equine/France/1/1987 | EPI_ISL_257669 | | | EPI969042 | | |  |  |  |
| A/equine/France/1/1991 | EPI_ISL_257670 | | | EPI969043 | | |  |  |  |
| A/equine/France/2/1985 | EPI_ISL_257666 | | | EPI969039 | | |  |  |  |
| A/equine/France/3/1985 | EPI_ISL_257667 | | | EPI969040 | | |  |  |  |
| A/equine/Gansu/7/2008 | EPI_ISL_32656 | | | EPI186856 | | |  |  |  |
| A/equine/Georgia/1/1981 | EPI_ISL_8095 | | | EPI77867 | | |  |  |  |
| A/equine/Georgia/10/1981 | EPI_ISL_8098 | | | EPI77924 | | |  |  |  |
| A/equine/Georgia/121362-16/2016 | EPI_ISL_294939 | | | EPI1157833 | | |  |  |  |
| A/equine/Georgia/13/1981 | EPI_ISL_8099 | | | EPI77943 | | |  |  |  |
| A/equine/Georgia/3/1981 | EPI_ISL_8096 | | | EPI77886 | | |  |  |  |
| A/equine/Georgia/9/1981 | EPI_ISL_8097 | | | EPI77905 | | |  |  |  |
| A/equine/Gironde/1/2014 | EPI_ISL_257682 | | | EPI969056 | | |  |  |  |
| A/Equine/Gloucestershire/2/16 | EPI_ISL_255686 | | | EPI957814 | | |  |  |  |
| A/Equine/Gloucestershire/3/16 | EPI_ISL_255687 | | | EPI957817 | | |  |  |  |
| A/equine/Gopeshwar/1/2009 | EPI_ISL_154970 | | | EPI503716 | | |  |  |  |
| A/equine/Gopeswar/1/2009 | EPI_ISL_74365 | | | EPI255568 | | |  |  |  |
| A/equine/Grosbois/1/1984 | EPI_ISL_257664 | | | EPI969037 | | |  |  |  |
| A/equine/Grosbois/1/1993 | EPI_ISL_257671 | | | EPI969044 | | |  |  |  |
| A/equine/Grosbois/1/1997 | EPI_ISL_257672 | | | EPI969045 | | |  |  |  |
| A/equine/Grosbois/1/1999 | EPI_ISL_257673 | | | EPI969046 | | |  |  |  |
| A/equine/Grosbois/1/2003 | EPI_ISL_257676 | | | EPI969049 | | |  |  |  |
| A/equine/Grosbois/13/2009 | EPI_ISL_257686 | | | EPI969053 | | |  |  |  |
| A/equine/Grosbois/2/1999 | EPI_ISL_257674 | | | EPI969047 | | |  |  |  |
| A/equine/Guangxi/1/2008 | EPI_ISL_174545 | | | EPI573754 | | |  |  |  |
| A/equine/Hampshire/3/2016 | EPI_ISL_231828 | | | EPI957607 | | |  |  |  |
| A/equine/Heilongjiang/1/2010 | EPI_ISL_144923 | | | EPI464984 | | |  |  |  |
| A/equine/Heilongjiang/10/2008 | EPI_ISL_32658 | | | EPI186872 | | |  |  |  |
| A/equine/Heilongjiang/SS1/2013 | EPI_ISL_161474 | | | EPI527595 | | |  |  |  |
| A/equine/Hertfordshire/1/2013 | EPI_ISL_151797 | | | EPI497569 | | |  |  |  |
| A/equine/Himachal Pradesh/CMVL-YOL2/2008 | EPI_ISL_190904 | | | EPI621745 | | |  |  |  |
| A/equine/Hissar/CMVL-HSR4/2008 | EPI_ISL_190901 | | | EPI621726 | | |  |  |  |
| A/equine/Huabei/1/2007 | EPI_ISL_70195 | | | EPI243549 | | |  |  |  |
| A/equine/Hubei/6/2008 | EPI_ISL_32657 | | | EPI186864 | | |  |  |  |
| A/equine/Ibaraki/1/07 | EPI_ISL_63503 | | | EPI222040 | | |  |  |  |
| A/equine/Ibaraki/1/2007 | EPI_ISL_297956 | | | EPI1172964 | | |  |  |  |
| A/equine/Idaho/37875/1991 | EPI_ISL_6623 | | | EPI52072 | | |  |  |  |
| A/equine/Inner Mongolia/8/2008 | EPI_ISL_32660 | | | EPI186888 | | |  |  |  |
| A/equine/Italy/1062/1991 | EPI_ISL_19678 | | | EPI153980 | | |  |  |  |
| A/equine/Italy/1199/1992 | EPI_ISL_19674 | | | EPI153948 | | |  |  |  |
| A/equine/Italy/824/1991 | EPI_ISL_19673 | | | EPI153940 | | |  |  |  |
| A/equine/Jammu and Kashmir/CMVL-LEH4/2008 | EPI_ISL_190902 | | | EPI621732 | | |  |  |  |
| A/equine/Jammu and Kashmir/CMVL-LEH6/2008 | EPI_ISL_190903 | | | EPI621738 | | |  |  |  |
| A/equine/Johannesburg/1/1986 | EPI_ISL_20809 | | | EPI156259 | | |  |  |  |
| A/equine/Jouars/4/2006 | EPI_ISL_257684 | | | EPI969052 | | |  |  |  |
| A/equine/Kanazawa/1/2007 | EPI_ISL_666 | | | EPI153119 | | |  |  |  |
| A/equine/Kascakew/1/1978 | EPI_ISL_69898 | | | EPI242355 | | |  |  |  |
| A/equine/Katra-Jammu/7/2008 | EPI_ISL_74366 | | | EPI503715 | | |  |  |  |
| A/Equine/Kent/1/15 | EPI_ISL_240663 | | | EPI873592 | | |  |  |  |
| A/Equine/Kent/1/16 | EPI_ISL_255630 | | | EPI957609 | | |  |  |  |
| A/equine/Kent/1/2014 | EPI_ISL_234916 | | | EPI839722 | | |  |  |  |
| A/equine/Kent/1/2015 | EPI_ISL_201991 | | | EPI673531 | | |  |  |  |
| A/equine/Kentucky/1/11 | EPI_ISL_177500 | | | EPI584280 | | |  |  |  |
| A/equine/Kentucky/1/1978 | EPI_ISL_8087 | | | EPI77715 | | |  |  |  |
| A/equine/Kentucky/1/1981 | EPI_ISL_68957 | | | EPI240112 | | |  |  |  |
| A/equine/Kentucky/1/1986 | EPI_ISL_15344 | | | EPI133071 | | |  |  |  |
| A/equine/Kentucky/1/1987 | EPI_ISL_21060 | | | EPI133090 | | |  |  |  |
| A/equine/Kentucky/1/1991 | EPI_ISL_15352 | | | EPI133261 | | |  |  |  |
| A/equine/Kentucky/1/1992 | EPI_ISL_15350 | | | EPI133204 | | |  |  |  |
| A/equine/Kentucky/1/2011 | EPI_ISL_201105 | | | EPI670002 | | |  |  |  |
| A/equine/Kentucky/1/2012 | EPI_ISL_201106 | | | EPI670003 | | |  |  |  |
| A/Equine/Kentucky/1/2014 | EPI_ISL_206059 | | | EPI687836 | | |  |  |  |
| A/equine/Kentucky/1277/1990 | EPI_ISL_15348 | | | EPI133166 | | |  |  |  |
| A/equine/Kentucky/2/1980 | EPI_ISL_20807 | | | EPI156243 | | |  |  |  |
| A/equine/Kentucky/2/1981 | EPI_ISL_8090 | | | EPI77772 | | |  |  |  |
| A/equine/Kentucky/2/1986 | EPI_ISL_21280 | | | EPI153844 | | |  |  |  |
| A/equine/Kentucky/2/1987 | EPI_ISL_19104 | | | EPI152771 | | |  |  |  |
| A/equine/Kentucky/2/2012 | EPI_ISL_153481 | | | EPI499161 | | |  |  |  |
| A/equine/Kentucky/3/1981 | EPI_ISL_15342 | | | EPI133033 | | |  |  |  |
| A/equine/Kentucky/3/1986 | EPI_ISL_19663 | | | EPI153852 | | |  |  |  |
| A/equine/Kentucky/3/2012 | EPI_ISL_153482 | | | EPI499160 | | |  |  |  |
| A/equine/Kentucky/4/1980 | EPI_ISL_8088 | | | EPI77734 | | |  |  |  |
| A/equine/Kentucky/4/2012 | EPI_ISL_153493 | | | EPI499173 | | |  |  |  |
| A/equine/Kentucky/5/02 | EPI_ISL_4663 | | | EPI25746 | | |  |  |  |
| A/equine/Kentucky/5/2002 | EPI_ISL_9530 | | | EPI98498 | | |  |  |  |
| A/equine/Kentucky/5/2012 | EPI_ISL_153492 | | | EPI499172 | | |  |  |  |
| A/equine/Kentucky/692/1988 | EPI_ISL_15345 | | | EPI133109 | | |  |  |  |
| A/equine/Kentucky/694/1988 | EPI_ISL_15346 | | | EPI133128 | | |  |  |  |
| A/equine/Kentucky/698/1988 | EPI_ISL_15347 | | | EPI133147 | | |  |  |  |
| A/equine/Kentucky/8/1994 | EPI_ISL_15353 | | | EPI133280 | | |  |  |  |
| A/equine/Kentucky/bitter_boredom5/1976 | EPI_ISL_8094 | | | EPI77848 | | |  |  |  |
| A/equine/Kentucky/magnificent_genius1/1981 | EPI_ISL_8100 | | | EPI77962 | | |  |  |  |
| A/equine/Kentucky/pass_the_pepper1/1976 | EPI_ISL_8093 | | | EPI77829 | | |  |  |  |
| A/equine/Kentucky/Rosie100/1981 | EPI_ISL_15830 | | | EPI137533 | | |  |  |  |
| A/equine/Kostanay/09/2012 | EPI_ISL_170790 | | | EPI558695 | | |  |  |  |
| A/equine/Kyonggi/SA1/2011 | EPI_ISL_129971 | | | EPI398889 | | |  |  |  |
| A/equine/Lanarkshire/09 | EPI_ISL_177490 | | | EPI584197 | | |  |  |  |
| A/equine/Lanarkshire/1/2013 | EPI_ISL_151981 | | | EPI493613 | | |  |  |  |
| A/equine/Lanarkshire/2/2013 | EPI_ISL_151982 | | | EPI493615 | | |  |  |  |
| A/equine/Lanarkshire/2/2015 | EPI_ISL_197572 | | | EPI650054 | | |  |  |  |
| A/equine/Lanarkshire/2009 | EPI_ISL_153496 | | | EPI499176 | | |  |  |  |
| A/Equine/Lancashire/1/16 | EPI_ISL_255635 | | | EPI957670 | | |  |  |  |
| A/equine/Leicestershire/1/2015 | EPI_ISL_205957 | | | EPI694842 | | |  |  |  |
| A/equine/Liaoning/9/2008 | EPI_ISL_32659 | | | EPI186880 | | |  |  |  |
| A/equine/Lichtenfeld/1/2012 | EPI_ISL_153494 | | | EPI499174 | | |  |  |  |
| A/equine/Lincolnshire/06 | EPI_ISL_177471 | | | EPI584129 | | |  |  |  |
| A/equine/Lincolnshire/1/2007 | EPI_ISL_29831 | | | EPI501407 | | |  |  |  |
| A/equine/Lincolnshire/2006 | EPI_ISL_153489 | | | EPI499169 | | |  |  |  |
| A/equine/Lincolnshire/2007 | EPI_ISL_153491 | | | EPI499171 | | |  |  |  |
| A/equine/LKZ/09/2012 | EPI_ISL_170788 | | | EPI558692 | | |  |  |  |
| A/equine/Lonquen/1/2006 | EPI_ISL_23100 | | | EPI161637 | | |  |  |  |
| A/Equine/Ludhiana/87 | EPI_ISL_68862 | | | EPI239905 | | |  |  |  |
| A/equine/Malaysia/M201/2015 | EPI_ISL_201434 | | | EPI671849 | | |  |  |  |
| A/Equine/Malaysia/M201-1/2015 (H3N8) | EPI_ISL_200161 | | | EPI667287 | | |  |  |  |
| A/Equine/Malaysia/M201-2/2015 (H3N8) | EPI_ISL_200622 | | | EPI667288 | | |  |  |  |
| A/equine/Marseille/1/1979 | EPI_ISL_257660 | | | EPI969033 | | |  |  |  |
| A/equine/Matybulak/10/2012 | EPI_ISL_170789 | | | EPI558693 | | |  |  |  |
| A/equine/Miami/1/1963 | EPI_ISL_8092 | | | EPI128920 | | |  |  |  |
| A/equine/Miami/1/1963 | EPI_ISL_8092 | | | EPI128920 | | |  |  |  |
| A/equine/Mongolia/3/2011 | EPI_ISL_129371 | | | EPI395969 | | |  |  |  |
| A/equine/Montana/9233/2007 | EPI_ISL_79893 | | | EPI281549 | | |  |  |  |
| A/equine/Montana/9564-1/2015 | EPI_ISL_285417 | | | EPI1103410 | | |  |  |  |
| A/equine/Moulton/98 | EPI_ISL_177487 | | | EPI584176 | | |  |  |  |
| A/equine/Mysore/1/2008 | EPI_ISL_74367 | | | EPI255571 | | |  |  |  |
| A/equine/Mysore/12/2008 | EPI_ISL_84310 | | | EPI503717 | | |  |  |  |
| A/equine/Neuville-Pres-Sees/1/2011 | EPI_ISL_257679 | | | EPI969051 | | |  |  |  |
| A/equine/New Hampshire/1/2013 | EPI_ISL_201107 | | | EPI670004 | | |  |  |  |
| A/equine/New Hampshire/2/2013 | EPI_ISL_201108 | | | EPI670005 | | |  |  |  |
| A/equine/New Market/1979 | EPI_ISL_69902 | | | EPI243224 | | |  |  |  |
| A/equine/New York/1/1983 | EPI_ISL_15356 | | | EPI133337 | | |  |  |  |
| A/equine/New York/1/1999 | EPI_ISL_9531 | | | EPI98500 | | |  |  |  |
| A/equine/New York/135857/2016 | EPI_ISL_294936 | | | EPI1157848 | | |  |  |  |
| A/equine/New York/146066/2007 | EPI_ISL_79900 | | | EPI281605 | | |  |  |  |
| A/equine/New York/452/2003 | EPI_ISL_9532 | | | EPI98504 | | |  |  |  |
| A/equine/New York/VR-297/1983 | EPI_ISL_8102 | | | EPI78000 | | |  |  |  |
| A/equine/Newmarket/1/1993 | EPI_ISL_30895 | | | EPI239735 | | |  |  |  |
| A/equine/Newmarket/2/1993 | EPI_ISL_30896 | | | EPI239741 | | |  |  |  |
| A/equine/Newmarket/5/2003 | EPI_ISL_30894 | | | EPI235163 | | |  |  |  |
| A/equine/Norfolk/1/2015 | EPI_ISL_205763 | | | EPI686737 | | |  |  |  |
| A/equine/North Yorkshire/1/2015 | EPI_ISL_205764 | | | EPI686739 | | |  |  |  |
| A/equine/North Yorkshire/2/2015 | EPI_ISL_207225 | | | EPI699681 | | |  |  |  |
| A/equine/Northamptonshire/1/2013 | EPI_ISL_151841 | | | EPI493625 | | |  |  |  |
| A/equine/Northamptonshire/2/2015 | EPI_ISL_205765 | | | EPI839779 | | |  |  |  |
| A/equine/Northamptonshire/3/2013 | EPI_ISL_234617 | | | EPI838703 | | |  |  |  |
| A/equine/Northamptonshire/3/2013 | EPI_ISL_234617 | | | EPI838703 | | |  |  |  |
| A/equine/Northamptonshire/3/2015 | EPI_ISL_234921 | | | EPI839809 | | |  |  |  |
| A/equine/Northamptonshire/4/2013 | EPI_ISL_152873 | | | EPI497573 | | |  |  |  |
| A/equine/Northamptonshire/4/2015 | EPI_ISL_234922 | | | EPI839822 | | |  |  |  |
| A/equine/Northamptonshire/5/2013 | EPI_ISL_152874 | | | EPI497574 | | |  |  |  |
| A/equine/Northamptonshire/5/2013 | EPI_ISL_152874 | | | EPI497574 | | |  |  |  |
| A/equine/Northamptonshire/7/2013 | EPI_ISL_151984 | | | EPI493617 | | |  |  |  |
| A/equine/Ohio/1/2003 | EPI_ISL_9534 | | | EPI98508 | | |  |  |  |
| A/equine/Ohio/1/2013 | EPI_ISL_201109 | | | EPI670006 | | |  |  |  |
| A/equine/Ohio/113461-1/2005 | EPI_ISL_79869 | | | EPI281357 | | |  |  |  |
| A/equine/Ohio/113461-2/2005 | EPI_ISL_79866 | | | EPI281333 | | |  |  |  |
| A/equine/Ohio/113461-2/2005 | EPI_ISL_79866 | | | EPI281333 | | |  |  |  |
| A/equine/Ohio/113461-3/2005 | EPI_ISL_79870 | | | EPI281365 | | |  |  |  |
| A/equine/Ohio/2/2013 | EPI_ISL_201110 | | | EPI670007 | | |  |  |  |
| A/equine/Oregon/1/2013 | EPI_ISL_201111 | | | EPI670008 | | |  |  |  |
| A/equine/Oregon/3/2013 | EPI_ISL_201112 | | | EPI670009 | | |  |  |  |
| A/equine/Oregon/78356/2012 | EPI_ISL_294934 | | | EPI1157811 | | |  |  |  |
| A/equine/Otar/764/2007 | EPI_ISL_89156 | | | EPI314113 | | |  |  |  |
| A/equine/Pennsylvania/1-2/2011 | EPI_ISL_153475 | | | EPI499159 | | |  |  |  |
| A/equine/Pennsylvania/2-5/2011 | EPI_ISL_153476 | | | EPI499158 | | |  |  |  |
| A/equine/Pennsylvania/3-7/2011 | EPI_ISL_153477 | | | EPI499157 | | |  |  |  |
| A/equine/Pennsylvania/5-5/2011 | EPI_ISL_153478 | | | EPI499156 | | |  |  |  |
| A/equine/Pennsylvania/6-15/2011 | EPI_ISL_153479 | | | EPI499155 | | |  |  |  |
| A/equine/Perthshire/1/2014 | EPI_ISL_234911 | | | EPI839712 | | |  |  |  |
| A/equine/Perthshire/2/2014 | EPI_ISL_234914 | | | EPI839718 | | |  |  |  |
| A/equine/Perthshire/3/09 | EPI_ISL_177492 | | | EPI584213 | | |  |  |  |
| A/equine/Perthshire/3/2009 | EPI_ISL_153498 | | | EPI499179 | | |  |  |  |
| A/equine/Qinghai/1/1994 | EPI_ISL_32661 | | | EPI186896 | | |  |  |  |
| A/equine/Richmond/1/2007 | EPI_ISL_153499 | | | EPI499180 | | |  |  |  |
| A/equine/Richmond/1/2007 | EPI_ISL_153499 | | | EPI499180 | | |  |  |  |
| A/equine/Rio Grande do Sul/1/12 | EPI_ISL_177502 | | | EPI584297 | | |  |  |  |
| A/equine/Roma/5/1991 | EPI_ISL_19677 | | | EPI153972 | | |  |  |  |
| A/equine/Romania/1/1980 | EPI_ISL_19680 | | | EPI153996 | | |  |  |  |
| A/equine/Roxburghshire/1/2012 | EPI_ISL_153468 | | | EPI499164 | | |  |  |  |
| A/equine/Sachiyama/1/1971 | EPI_ISL_25008 | | | EPI165403 | | |  |  |  |
| A/equine/Santa Fe/1/1985 | EPI_ISL_19672 | | | EPI153932 | | |  |  |  |
| A/equine/Santiago/1/1985 | EPI_ISL_3674 | | | EPI19628 | | |  |  |  |
| A/equine/Sao Paulo/1.19/2012 | EPI_ISL_279065 | | | EPI1065025 | | |  |  |  |
| A/equine/Sao Paulo/1/1969 | EPI_ISL_19681 | | | EPI154004 | | |  |  |  |
| A/equine/Sao_Paulo/10.FMVZ/2015 | EPI_ISL_285491 | | | EPI1103591 | | |  |  |  |
| A/equine/Sao_Paulo/12.FMVZ/2015 | EPI_ISL_285493 | | | EPI1103592 | | |  |  |  |
| A/equine/Sao_Paulo/15.FMVZ/2015 | EPI_ISL_285494 | | | EPI1103593 | | |  |  |  |
| A/equine/Sao_Paulo/25.FMVZ/2015 | EPI_ISL_285495 | | | EPI1103594 | | |  |  |  |
| A/equine/Sao Paulo/6/1963 | EPI_ISL_19670 | | | EPI153916 | | |  |  |  |
| A/equine/Sao paulo/6/1969 | EPI_ISL_14482 | | | EPI128924 | | |  |  |  |
| A/equine/Sao_Paulo/8.FMVZ/2015 | EPI_ISL_285490 | | | EPI1103590 | | |  |  |  |
| A/equine/Saone-et-Loire/1/2015 | EPI_ISL_257683 | | | EPI969057 | | |  |  |  |
| A/equine/Scottish Borders/1/2014 | EPI_ISL_234913 | | | EPI839716 | | |  |  |  |
| A/equine/Scottish Borders/3/2015 | EPI_ISL_212044 | | | EPI710986 | | |  |  |  |
| A/equine/Shropshire/1/2013 | EPI_ISL_234615 | | | EPI838699 | | |  |  |  |
| A/equine/Shropshire/10 | EPI_ISL_156748 | | | EPI584229 | | |  |  |  |
| A/equine/Shropshire/2/2013 | EPI_ISL_234616 | | | EPI838701 | | |  |  |  |
| A/equine/Shropshire/2010 | EPI_ISL_153451 | | | EPI499167 | | |  |  |  |
| A/equine/Shropshire/7/2013 | EPI_ISL_151987 | | | EPI493624 | | |  |  |  |
| A/equine/Shropshire/8/2013 | EPI_ISL_151986 | | | EPI493623 | | |  |  |  |
| A/equine/Snailwell/98 | EPI_ISL_177489 | | | EPI584189 | | |  |  |  |
| A/Equine/South Africa/4/03 | EPI_ISL_240665 | | | EPI873600 | | |  |  |  |
| A/equine/South Kazakhstan/236/2012 | EPI_ISL_166607 | | | EPI542620 | | |  |  |  |
| A/equine/South Lanarkshire/1/2015 | EPI_ISL_195790 | | | EPI643193 | | |  |  |  |
| A/equine/Spain/1/2007 | EPI_ISL_81729 | | | EPI288544 | | |  |  |  |
| A/equine/Spain/1/2009 | EPI_ISL_81728 | | | EPI288542 | | |  |  |  |
| A/equine/Staffordshire/1/2014 | EPI_ISL_234912 | | | EPI839714 | | |  |  |  |
| A/Equine/Stirlingshire/1/16 | EPI_ISL_255633 | | | EPI957635 | | |  |  |  |
| A/equine/Sussex/1/1989 | EPI_ISL_19884 | | | EPI240131 | | |  |  |  |
| A/equine/Sussex/1/1989 | EPI_ISL_19884 | | | EPI240131 | | |  |  |  |
| A/equine/Sussex/93753/89 | EPI_ISL_8280 | | | EPI154462 | | |  |  |  |
| A/Equine/Sweden/SVA111128SZ0073VIR175146/2011 | EPI_ISL_190440 | | | EPI620297 | | |  |  |  |
| A/Equine/Sweden/SVA111206SZ0085/VIR165837/2011 | EPI_ISL_180684 | | | EPI594018 | | |  |  |  |
| A/Equine/Sweden/SVA111206SZ0085VIR165837/2011 | EPI_ISL_190419 | | | EPI620255 | | |  |  |  |
| A/Equine/Sweden/SVA111209SZ0099VIR169122/2011 | EPI_ISL_190424 | | | EPI620265 | | |  |  |  |
| A/Equine/Sweden/SVA111209SZ0099VIR169124/2011 | EPI_ISL_190425 | | | EPI620267 | | |  |  |  |
| A/Equine/Sweden/SVA111209SZ0099VIR169126/2011 | EPI_ISL_190426 | | | EPI620269 | | |  |  |  |
| A/Equine/Sweden/SVA111209SZ0099VIR169128/2011 | EPI_ISL_190427 | | | EPI620271 | | |  |  |  |
| A/Equine/Sweden/SVA111209SZ0099VIR169130/2011 | EPI_ISL_190428 | | | EPI620273 | | |  |  |  |
| A/Equine/Sweden/SVA111209SZ0099VIR169132/2011 | EPI_ISL_190429 | | | EPI620275 | | |  |  |  |
| A/Equine/Sweden/SVA111212SZ0056VIR169815/2011 | EPI_ISL_190430 | | | EPI620277 | | |  |  |  |
| A/Equine/Sweden/SVA111213SZ0065VIR170334/2011 | EPI_ISL_190431 | | | EPI620279 | | |  |  |  |
| A/Equine/Sweden/SVA111213SZ0065VIR170335/2011 | EPI_ISL_190432 | | | EPI620281 | | |  |  |  |
| A/Equine/Sweden/SVA111213SZ0070VIR170336/2011 | EPI_ISL_190433 | | | EPI620283 | | |  |  |  |
| A/Equine/Sweden/SVA111213SZ0070VIR170337/2011 | EPI_ISL_190434 | | | EPI620285 | | |  |  |  |
| A/Equine/Sweden/SVA111219SZ0079VIR174292/2011 | EPI_ISL_190435 | | | EPI620287 | | |  |  |  |
| A/Equine/Sweden/SVA111219SZ0079VIR174293/2011 | EPI_ISL_190436 | | | EPI620289 | | |  |  |  |
| A/Equine/Sweden/SVA111220SZ0255VIR175145/2011 | EPI_ISL_190437 | | | EPI620291 | | |  |  |  |
| A/Equine/Sweden/SVA111221SZ0085BKT087424/2011 | EPI_ISL_190438 | | | EPI620293 | | |  |  |  |
| A/Equine/Sweden/SVA111222SZ0168VIR177856/2011 | EPI_ISL_190439 | | | EPI620295 | | |  |  |  |
| A/equine/Switzerland/1118/1979 | EPI_ISL_19679 | | | EPI153988 | | |  |  |  |
| A/equine/Switzerland/173/1993 | EPI_ISL_19676 | | | EPI153964 | | |  |  |  |
| A/equine/Switzerland/2225/1979 | EPI_ISL_22611 | | | EPI159274 | | |  |  |  |
| A/equine/Switzerland/P112/07 | EPI_ISL_177468 | | | EPI584118 | | |  |  |  |
| A/equine/Sydney/6085/2007 | EPI_ISL_63504 | | | EPI222042 | | |  |  |  |
| A/equine/Tennessee/27A/2014 | EPI_ISL_220483 | | | EPI753464 | | |  |  |  |
| A/equine/Tennessee/28A/2014 | EPI_ISL_220480 | | | EPI753427 | | |  |  |  |
| A/equine/Tennessee/28B/2014 | EPI_ISL_220484 | | | EPI753452 | | |  |  |  |
| A/equine/Tennessee/29A/2014 | EPI_ISL_220485 | | | EPI753465 | | |  |  |  |
| A/equine/Tennessee/30A/2014 | EPI_ISL_220482 | | | EPI753440 | | |  |  |  |
| A/equine/Tennessee/4A/2014 | EPI_ISL_220481 | | | EPI753434 | | |  |  |  |
| A/equine/Tennessee/5/1986 | EPI_ISL_21061 | | | EPI133242 | | |  |  |  |
| A/equine/Tennessee/5/1986 | EPI_ISL_21061 | | | EPI133242 | | |  |  |  |
| A/equine/Texas/117793/2005 | EPI_ISL_79867 | | | EPI281341 | | |  |  |  |
| A/equine/Texas/117793/2005 | EPI_ISL_79867 | | | EPI281341 | | |  |  |  |
| A/equine/Texas/39655/1991 | EPI_ISL_15349 | | | EPI133185 | | |  |  |  |
| A/equine/Tiaret/1/2011 | EPI_ISL_144944 | | | EPI468652 | | |  |  |  |
| A/equine/Tokyo/2/1971 | EPI_ISL_95197 | | | EPI332913 | | |  |  |  |
| A/equine/Tottori/1/07 | EPI_ISL_89221 | | | EPI314450 | | |  |  |  |
| A/equine/Tyne&Wear/1/2015 | EPI_ISL_197574 | | | EPI651799 | | |  |  |  |
| A/equine/Tyne&Wear/2/2015 | EPI_ISL_197573 | | | EPI650055 | | |  |  |  |
| A/equine/Uruguay/1/1963 | EPI_ISL_21282 | | | EPI154028 | | |  |  |  |
| A/equine/Uttarkashi/1/2009 | EPI_ISL_154969 | | | EPI503714 | | |  |  |  |
| A/equine/Virginia/131054-3/2005 | EPI_ISL_79899 | | | EPI281597 | | |  |  |  |
| A/equine/Warwickshire/1/2013 | EPI_ISL_151846 | | | EPI497571 | | |  |  |  |
| A/equine/West Lothian/1/2014 | EPI_ISL_234908 | | | EPI839656 | | |  |  |  |
| A/equine/West Midlands/1/2014 | EPI_ISL_234917 | | | EPI839724 | | |  |  |  |
| A/equine/West Midlands/1/2015 | EPI_ISL_197571 | | | EPI650052 | | |  |  |  |
| A/equine/Wisconsin/1/03 | EPI_ISL_9783 | | | EPI99378 | | |  |  |  |
| A/equine/Worcestershire/1/2012 | EPI_ISL_153488 | | | EPI499168 | | |  |  |  |
| A/equine/Worcestershire/1/2013 | EPI_ISL_151845 | | | EPI497570 | | |  |  |  |
| A/equine/Worcestershire/2/2012 | EPI_ISL_153465 | | | EPI499162 | | |  |  |  |
| A/equine/Worcestershire/2/2014 | EPI_ISL_234915 | | | EPI839720 | | |  |  |  |
| A/equine/Worcestershire/4/2012 | EPI_ISL_153467 | | | EPI499163 | | |  |  |  |
| A/equine/Xinjiang/1/2007 | EPI_ISL_32662 | | | EPI186904 | | |  |  |  |
| A/equine/Xinjiang/2/2007 | EPI_ISL_32663 | | | EPI186912 | | |  |  |  |
| A/equine/Xinjiang/3/2007 | EPI_ISL_32664 | | | EPI186920 | | |  |  |  |
| A/equine/Xinjiang/4/2007 | EPI_ISL_32665 | | | EPI186928 | | |  |  |  |
| A/equine/Xinjiang/5/2007 | EPI_ISL_32666 | | | EPI186936 | | |  |  |  |
| A/equine/Xuzhou/01/2013 | EPI_ISL_151130 | | | EPI489632 | | |  |  |  |
| A/equine/Yokohama/aq13/2010 | EPI_ISL_297955 | | | EPI1172956 | | |  |  |  |
| A/equine/Yokohama/aq13/2010 | EPI_ISL_297955 | | | EPI1172956 | | |  |  |  |
| A/equine/Yokohama/aq19/2009 | EPI_ISL_69743 | | | EPI241777 | | |  |  |  |
| A/equine/Yokohama/aq29/2011 | EPI_ISL_121371 | | | EPI376183 | | |  |  |  |
| A/equine/Yokohama/aq5/2011 | EPI_ISL_121370 | | | EPI376181 | | |  |  |  |
| A/equine/Yokohama/aq53/2011 | EPI_ISL_121372 | | | EPI376185 | | |  |  |  |
| A/equine/Yokohama/aq79/2011 | EPI_ISL_121373 | | | EPI376187 | | |  |  |  |
| A/equine/Yorkshire/3/09 | EPI_ISL_156745 | | | EPI584221 | | |  |  |  |
| A/Ken/1/1981 | EPI_ISL_66100 | | | EPI230581 | | |  |  |  |
| A/Lincolnshire/1/2013 | EPI_ISL_234646 | | | EPI838919 | | |  |  |  |
| A /Equine/Sweden/SVA111128SZ0073/VIR160172/2011 | EPI_ISL_180755 | | | EPI594822 | | |  |  |  |
| A Equine Sweden SVA111128SZ0073VIR160172 2011 | EPI_ISL_190441 | | | EPI620299 | | |  |  |  |
| A Equine Sweden SVA111208SZ0077VIR167905 2011 | EPI_ISL_190420 | | | EPI620257 | | |  |  |  |
| #A Equine Sweden SVA111208SZ0077VIR167906 2011 | EPI_ISL_190421 | | | EPI620259 | | |  |  |  |
| A Equine Sweden SVA111209SZ0099VIR169118 2011 | EPI_ISL_190423 | | | EPI620263 | | |  |  |  |
| A Equine Sweden SVA111212SZ0058VIR169816 2011 | EPI_ISL_190422 | | | EPI620261 | | |  |  |  |
| NP | | | | | | |  |  |  |
| Isolate_Name | Isolate_Id | | | Segment_Id | | |  |  |  |
| A/canine/California/70645-4/2006 | EPI_ISL_79878 | | | EPI281428 | | |  |  |  |
| A/canine/Colorado/17864/2006 | EPI_ISL_79875 | | | EPI281404 | | |  |  |  |
| A/canine/Colorado/17864/2006 | EPI_ISL_79875 | | | EPI281404 | | |  |  |  |
| A/canine/Colorado/30604/2006 | EPI_ISL_79877 | | | EPI281420 | | |  |  |  |
| A/canine/Colorado/6723-8/2008 | EPI_ISL_79901 | | | EPI281612 | | |  |  |  |
| A/canine/Colorado/6723-8/2008 | EPI_ISL_79901 | | | EPI281612 | | |  |  |  |
| A/canine/Colorado/8880/2006 | EPI_ISL_79874 | | | EPI281396 | | |  |  |  |
| A/canine/Connecticut/85863/2011 | EPI_ISL_294935 | | | EPI1157813 | | |  |  |  |
| A/canine/CT/85863/2011 | EPI_ISL_167698 | | | EPI546851 | | |  |  |  |
| A/canine/Florida/15592.1/2004 | EPI_ISL_294945 | | | EPI1157850 | | |  |  |  |
| A/canine/Florida/242/2003 | EPI_ISL_9528 | | | EPI98485 | | |  |  |  |
| A/canine/Florida/43/2004 | EPI_ISL_9527 | | | EPI98467 | | |  |  |  |
| A/canine/Florida/61156-2/2006 | EPI_ISL_79895 | | | EPI281564 | | |  |  |  |
| A/canine/Florida/61156-2/2006 | EPI_ISL_79895 | | | EPI281564 | | |  |  |  |
| A/canine/Florida/78592-2/2006 | EPI_ISL_79879 | | | EPI281436 | | |  |  |  |
| A/canine/Florida/78592-6/2006 | EPI_ISL_79880 | | | EPI281444 | | |  |  |  |
| A/canine/Florida/78592-7/2006 | EPI_ISL_79881 | | | EPI281452 | | |  |  |  |
| A/canine/Florida/89911-2/2006 | EPI_ISL_79882 | | | EPI281460 | | |  |  |  |
| A/canine/Jacksonville/2005 | EPI_ISL_64818 | | | EPI226910 | | |  |  |  |
| A/canine/Kentucky/118778/2006 | EPI_ISL_79889 | | | EPI281516 | | |  |  |  |
| A/canine/Maine/058124/2016 | EPI_ISL_294942 | | | EPI1157825 | | |  |  |  |
| A/canine/Massachusetts/26810/2016 | EPI_ISL_234612 | | | EPI839111 | | |  |  |  |
| A/canine/Miami/2005 | EPI_ISL_64819 | | | EPI226916 | | |  |  |  |
| A/canine/New York/100525-1/2006 | EPI_ISL_79883 | | | EPI281468 | | |  |  |  |
| A/canine/New York/100528-1/2006 | EPI_ISL_79884 | | | EPI281476 | | |  |  |  |
| A/canine/New York/100528-5/2006 | EPI_ISL_79885 | | | EPI281484 | | |  |  |  |
| A/canine/New York/100528-6/2006 | EPI_ISL_79886 | | | EPI281492 | | |  |  |  |
| A/canine/New York/115719/2007 | EPI_ISL_79896 | | | EPI281572 | | |  |  |  |
| A/canine/New York/115809/2005 | EPI_ISL_79864 | | | EPI281316 | | |  |  |  |
| A/canine/New York/145353/2008 | EPI_ISL_79904 | | | EPI281636 | | |  |  |  |
| A/canine/New York/147926-3/2006 | EPI_ISL_79887 | | | EPI281500 | | |  |  |  |
| A/canine/New York/147926-5/2006 | EPI_ISL_79888 | | | EPI281508 | | |  |  |  |
| A/canine/New York/158402-1/2008 | EPI_ISL_79905 | | | EPI281644 | | |  |  |  |
| A/canine/New York/159903/2012 | EPI_ISL_294938 | | | EPI1157898 | | |  |  |  |
| A/canine/New York/1623.1/2010 | EPI_ISL_294943 | | | EPI1157886 | | |  |  |  |
| A/canine/New York/3699/2010 | EPI_ISL_294940 | | | EPI1157885 | | |  |  |  |
| A/canine/New York/4986-2/2006 | EPI_ISL_79873 | | | EPI281388 | | |  |  |  |
| A/canine/New York/5183-6/2006 | EPI_ISL_79871 | | | EPI281372 | | |  |  |  |
| A/canine/New York/5183-6/2006 | EPI_ISL_79871 | | | EPI281372 | | |  |  |  |
| A/canine/New York/51854/2008 | EPI_ISL_79902 | | | EPI281620 | | |  |  |  |
| A/canine/NY/105447/2008 | EPI_ISL_174477 | | | EPI576529 | | |  |  |  |
| A/canine/NY/120106.2/2011 | EPI_ISL_166503 | | | EPI546847 | | |  |  |  |
| A/canine/NY/133/2010 | EPI_ISL_167704 | | | EPI546858 | | |  |  |  |
| A/canine/NY/159903/2012 | EPI_ISL_167700 | | | EPI546853 | | |  |  |  |
| A/canine/NY/1623.1/2010 | EPI_ISL_167705 | | | EPI546859 | | |  |  |  |
| A/canine/NY/3699/2010 | EPI_ISL_167706 | | | EPI546860 | | |  |  |  |
| A/canine/NY/dog1c01/2009 | EPI_ISL_82037 | | | EPI289514 | | |  |  |  |
| A/canine/NY/dog1c11/2009 | EPI_ISL_82022 | | | EPI289515 | | |  |  |  |
| A/canine/NY/dog21c02/2009 | EPI_ISL_82102 | | | EPI289506 | | |  |  |  |
| A/canine/NY/dog21c08/2009 | EPI_ISL_81929 | | | EPI289505 | | |  |  |  |
| A/canine/NY/dog21c09/2009 | EPI_ISL_82183 | | | EPI289504 | | |  |  |  |
| A/canine/NY/dog21c24/2009 | EPI_ISL_82182 | | | EPI289503 | | |  |  |  |
| A/canine/NY/dog3c03/2009 | EPI_ISL_82137 | | | EPI289516 | | |  |  |  |
| A/canine/NY/dog4c05/2009 | EPI_ISL_82122 | | | EPI289511 | | |  |  |  |
| A/canine/NY/dog4c09/2009 | EPI_ISL_82131 | | | EPI289512 | | |  |  |  |
| A/canine/NY/dog4c20/2009 | EPI_ISL_82005 | | | EPI289513 | | |  |  |  |
| A/canine/NY/dog6c13/2009 | EPI_ISL_82082 | | | EPI289510 | | |  |  |  |
| A/canine/NY/dog8c03/2008 | EPI_ISL_81971 | | | EPI289508 | | |  |  |  |
| A/canine/NY/dog8c12/2008 | EPI_ISL_81982 | | | EPI289509 | | |  |  |  |
| A/canine/NY/dog8c15/2008 | EPI_ISL_81978 | | | EPI289507 | | |  |  |  |
| A/canine/PA/111788.2/2009 | EPI_ISL_166506 | | | EPI546857 | | |  |  |  |
| A/canine/PA/27637.3/2010 | EPI_ISL_167697 | | | EPI546850 | | |  |  |  |
| A/canine/PA/33225.4/2010 | EPI_ISL_167696 | | | EPI546848 | | |  |  |  |
| A/canine/PA/96978/2009 | EPI_ISL_167699 | | | EPI546852 | | |  |  |  |
| A/canine/Pennsylvania/10909/2007 | EPI_ISL_79891 | | | EPI281532 | | |  |  |  |
| A/canine/Pennsylvania/10915/2007 | EPI_ISL_79892 | | | EPI281540 | | |  |  |  |
| A/canine/Pennsylvania/137154/2008 | EPI_ISL_79903 | | | EPI281628 | | |  |  |  |
| A/canine/Pennsylvania/16699/2007 | EPI_ISL_79890 | | | EPI281524 | | |  |  |  |
| A/canine/Pennsylvania/94930-3/2007 | EPI_ISL_79897 | | | EPI281580 | | |  |  |  |
| A/canine/Pennsylvania/96978/2009 | EPI_ISL_294937 | | | EPI1157871 | | |  |  |  |
| A/canine/Vermont/278213/2013 | EPI_ISL_294941 | | | EPI1157835 | | |  |  |  |
| A/canine/VT/11039/2013 | EPI_ISL_166505 | | | EPI546849 | | |  |  |  |
| A/canine/VT/2782/2013 | EPI_ISL_167701 | | | EPI546854 | | |  |  |  |
| A/donkey/Shandong/1/2017 | EPI_ISL_284681 | | | EPI1098481 | | |  |  |  |
| A/equine/Alaska/29759/1991 | EPI_ISL_15351 | | | EPI133225 | | |  |  |  |
| A/equine/Algiers/1/1972 | EPI_ISL_20808 | | | EPI156252 | | |  |  |  |
| A/equine/Almaty/26/2007 | EPI_ISL_71977 | | | EPI250109 | | |  |  |  |
| A/equine/Ankara/1/2013 | EPI_ISL_281349 | | | EPI1076907 | | |  |  |  |
| A/equine/Athens/02/2003 | EPI_ISL_75320 | | | EPI347183 | | |  |  |  |
| A/equine/Athens/04/2007 | EPI_ISL_28012 | | | EPI347184 | | |  |  |  |
| A/equine/Austria/421/1992 | EPI_ISL_19675 | | | EPI153957 | | |  |  |  |
| A/Equine/Ayrshire/1/2013 | EPI_ISL_240595 | | | EPI873573 | | |  |  |  |
| A/equine/Berlin/1/1989 | EPI_ISL_19683 | | | EPI154021 | | |  |  |  |
| A/equine/Buckinghamshire/1/2014 | EPI_ISL_197753 | | | EPI651399 | | |  |  |  |
| A/equine/California/1/10 | EPI_ISL_177498 | | | EPI584271 | | |  |  |  |
| A/equine/California/1/1980 | EPI_ISL_8089 | | | EPI77755 | | |  |  |  |
| A/equine/California/103/1982 | EPI_ISL_8101 | | | EPI77983 | | |  |  |  |
| A/equine/California/4537/1997 | EPI_ISL_15842 | | | EPI137763 | | |  |  |  |
| A/equine/California/83/1982 | EPI_ISL_15343 | | | EPI133054 | | |  |  |  |
| A/equine/California/8560/2002 | EPI_ISL_15843 | | | EPI137782 | | |  |  |  |
| A/equine/Cambremer/1/2012 | EPI_ISL_257680 | | | EPI969027 | | |  |  |  |
| A/equine/Cheshire/06 | EPI_ISL_177472 | | | EPI584136 | | |  |  |  |
| A/equine/Cordoba/18/1985 | EPI_ISL_19671 | | | EPI153925 | | |  |  |  |
| A/equine/Devon/1/11 | EPI_ISL_156749 | | | EPI584245 | | |  |  |  |
| A/equine/Dorset/09 | EPI_ISL_177491 | | | EPI584204 | | |  |  |  |
| A/equine/Dubai/1/12 | EPI_ISL_177501 | | | EPI584287 | | |  |  |  |
| A/equine/East Renfrewshire/2/11 | EPI_ISL_156750 | | | EPI584237 | | |  |  |  |
| A/Equine/East Sussex/1/2015 | EPI_ISL_240662 | | | EPI873582 | | |  |  |  |
| A/Equine/Fife/1/16 | EPI_ISL_255632 | | | EPI957625 | | |  |  |  |
| A/equine/FL/146609/2011 | EPI_ISL_167702 | | | EPI546855 | | |  |  |  |
| A/equine/Florida/146609/2011 | EPI_ISL_294944 | | | EPI1157866 | | |  |  |  |
| A/equine/Fontainbleu/1/1979 | EPI_ISL_19682 | | | EPI154013 | | |  |  |  |
| A/equine/France/1/1967 | EPI_ISL_22612 | | | EPI159283 | | |  |  |  |
| A/equine/Gansu/7/2008 | EPI_ISL_32656 | | | EPI186855 | | |  |  |  |
| A/equine/Georgia/1/1981 | EPI_ISL_8095 | | | EPI77869 | | |  |  |  |
| A/equine/Georgia/10/1981 | EPI_ISL_8098 | | | EPI77926 | | |  |  |  |
| A/equine/Georgia/121362-16/2016 | EPI_ISL_294939 | | | EPI1157839 | | |  |  |  |
| A/equine/Georgia/13/1981 | EPI_ISL_8099 | | | EPI77945 | | |  |  |  |
| A/equine/Georgia/3/1981 | EPI_ISL_8096 | | | EPI77888 | | |  |  |  |
| A/equine/Georgia/9/1981 | EPI_ISL_8097 | | | EPI77907 | | |  |  |  |
| A/equine/Gironde/1/2014 | EPI_ISL_257682 | | | EPI969028 | | |  |  |  |
| A/Equine/Gloucestershire/1/16 | EPI_ISL_255667 | | | EPI957690 | | |  |  |  |
| A/equine/Guangxi/1/2008 | EPI_ISL_174545 | | | EPI573753 | | |  |  |  |
| A/equine/Hampshire/3/2016 | EPI_ISL_231828 | | | EPI957613 | | |  |  |  |
| A/Equine/Hampshire/4/2016 | EPI_ISL_240664 | | | EPI873598 | | |  |  |  |
| A/equine/Heilongjiang/1/2010 | EPI_ISL_144923 | | | EPI464983 | | |  |  |  |
| A/equine/Heilongjiang/10/2008 | EPI_ISL_32658 | | | EPI186871 | | |  |  |  |
| A/equine/Heilongjiang/SS1/2013 | EPI_ISL_161474 | | | EPI527596 | | |  |  |  |
| A/equine/Himachal Pradesh/CMVL-YOL2/2008 | EPI_ISL_190904 | | | EPI621744 | | |  |  |  |
| A/equine/Hissar/CMVL-HSR4/2008 | EPI_ISL_190901 | | | EPI621725 | | |  |  |  |
| A/equine/Huabei/1/2007 | EPI_ISL_70195 | | | EPI243548 | | |  |  |  |
| A/equine/Hubei/6/2008 | EPI_ISL_32657 | | | EPI186863 | | |  |  |  |
| A/equine/Ibaraki/1/2007 | EPI_ISL_297956 | | | EPI1172963 | | |  |  |  |
| A/equine/Idaho/37875/1991 | EPI_ISL_6623 | | | EPI52074 | | |  |  |  |
| A/equine/Inner Mongolia/8/2008 | EPI_ISL_32660 | | | EPI186887 | | |  |  |  |
| A/equine/Italy/1062/1991 | EPI_ISL_19678 | | | EPI153981 | | |  |  |  |
| A/equine/Italy/1199/1992 | EPI_ISL_19674 | | | EPI153949 | | |  |  |  |
| A/equine/Italy/824/1991 | EPI_ISL_19673 | | | EPI153941 | | |  |  |  |
| A/equine/Jammu and Kashmir/CMVL-LEH4/2008 | EPI_ISL_190902 | | | EPI621731 | | |  |  |  |
| A/equine/Jammu and Kashmir/CMVL-LEH6/2008 | EPI_ISL_190903 | | | EPI621737 | | |  |  |  |
| A/equine/Johannesburg/1/1986 | EPI_ISL_20809 | | | EPI156260 | | |  |  |  |
| A/equine/Jouars/4/2006 | EPI_ISL_257684 | | | EPI969025 | | |  |  |  |
| A/equine/Kascakew/1/1978 | EPI_ISL_69898 | | | EPI242354 | | |  |  |  |
| A/Equine/Kent/1/15 | EPI_ISL_240663 | | | EPI873590 | | |  |  |  |
| A/Equine/Kent/1/16 | EPI_ISL_255630 | | | EPI957611 | | |  |  |  |
| A/equine/Kentucky/1/11 | EPI_ISL_177500 | | | EPI584279 | | |  |  |  |
| A/equine/Kentucky/1/1978 | EPI_ISL_8087 | | | EPI77717 | | |  |  |  |
| A/equine/Kentucky/1/1981 | EPI_ISL_68957 | | | EPI240113 | | |  |  |  |
| A/equine/Kentucky/1/1986 | EPI_ISL_15344 | | | EPI133073 | | |  |  |  |
| A/equine/Kentucky/1/1987 | EPI_ISL_21060 | | | EPI133092 | | |  |  |  |
| A/equine/Kentucky/1/1991 | EPI_ISL_15352 | | | EPI133263 | | |  |  |  |
| A/equine/Kentucky/1/1992 | EPI_ISL_15350 | | | EPI133206 | | |  |  |  |
| A/equine/Kentucky/1/81 | EPI_ISL_689 | | | EPI18477 | | |  |  |  |
| A/equine/Kentucky/1277/1990 | EPI_ISL_15348 | | | EPI133168 | | |  |  |  |
| A/equine/Kentucky/2/1980 | EPI_ISL_20807 | | | EPI156244 | | |  |  |  |
| A/equine/Kentucky/2/1981 | EPI_ISL_8090 | | | EPI77774 | | |  |  |  |
| A/equine/Kentucky/2/1986 | EPI_ISL_14705 | | | EPI129911 | | |  |  |  |
| A/equine/Kentucky/2/1986 | EPI_ISL_14705 | | | EPI129911 | | |  |  |  |
| A/equine/Kentucky/2/1987 | EPI_ISL_19104 | | | EPI152772 | | |  |  |  |
| A/equine/Kentucky/211/1987 | EPI_ISL_68958 | | | EPI240121 | | |  |  |  |
| A/equine/Kentucky/3/1981 | EPI_ISL_15342 | | | EPI133035 | | |  |  |  |
| A/equine/Kentucky/3/1986 | EPI_ISL_19663 | | | EPI153853 | | |  |  |  |
| A/equine/Kentucky/4/1980 | EPI_ISL_8088 | | | EPI77736 | | |  |  |  |
| A/equine/Kentucky/5/02 | EPI_ISL_4663 | | | EPI25744 | | |  |  |  |
| A/equine/Kentucky/692/1988 | EPI_ISL_15345 | | | EPI133111 | | |  |  |  |
| A/equine/Kentucky/694/1988 | EPI_ISL_15346 | | | EPI133130 | | |  |  |  |
| A/equine/Kentucky/698/1988 | EPI_ISL_15347 | | | EPI133149 | | |  |  |  |
| A/equine/Kentucky/8/1994 | EPI_ISL_15353 | | | EPI133282 | | |  |  |  |
| A/equine/Kentucky/bitter_boredom5/1976 | EPI_ISL_8094 | | | EPI77850 | | |  |  |  |
| A/equine/Kentucky/magnificent_genius1/1981 | EPI_ISL_8100 | | | EPI77964 | | |  |  |  |
| A/equine/Kentucky/pass_the_pepper1/1976 | EPI_ISL_8093 | | | EPI77831 | | |  |  |  |
| A/equine/Kentucky/Rosie100/1981 | EPI_ISL_15830 | | | EPI137535 | | |  |  |  |
| A/equine/Kyonggi/SA1/2011 | EPI_ISL_129971 | | | EPI398888 | | |  |  |  |
| A/equine/Lanarkshire/09 | EPI_ISL_177490 | | | EPI584196 | | |  |  |  |
| A/Equine/Lancashire/1/16 | EPI_ISL_255635 | | | EPI957672 | | |  |  |  |
| A/equine/Liaoning/9/2008 | EPI_ISL_32659 | | | EPI186879 | | |  |  |  |
| A/equine/Lincolnshire/06 | EPI_ISL_177471 | | | EPI584128 | | |  |  |  |
| A/equine/Lincolnshire/1/2007 | EPI_ISL_29831 | | | EPI501405 | | |  |  |  |
| A/equine/Lonquen/1/2006 | EPI_ISL_23100 | | | EPI161636 | | |  |  |  |
| A/equine/Miami/1/1963 | EPI_ISL_8092 | | | EPI129664 | | |  |  |  |
| A/equine/Miami/1/1963 | EPI_ISL_8092 | | | EPI129664 | | |  |  |  |
| A/equine/Montana/9233/2007 | EPI_ISL_79893 | | | EPI281548 | | |  |  |  |
| A/equine/Montana/9564-1/2015 | EPI_ISL_285417 | | | EPI1103409 | | |  |  |  |
| A/equine/Moulton/98 | EPI_ISL_177487 | | | EPI584175 | | |  |  |  |
| A/equine/Neuville-Pres-Sees/1/2011 | EPI_ISL_257679 | | | EPI969026 | | |  |  |  |
| A/equine/New Market/1/1979 | EPI_ISL_95199 | | | EPI332931 | | |  |  |  |
| A/equine/New Market/nasalwash1/1979 | EPI_ISL_95198 | | | EPI332924 | | |  |  |  |
| A/equine/New York/1/1983 | EPI_ISL_15356 | | | EPI133339 | | |  |  |  |
| A/equine/New York/135857/2016 | EPI_ISL_294936 | | | EPI1157807 | | |  |  |  |
| A/equine/New York/146066/2007 | EPI_ISL_79900 | | | EPI281604 | | |  |  |  |
| A/equine/New York/VR-297/1983 | EPI_ISL_8102 | | | EPI78002 | | |  |  |  |
| A/equine/Newmarket/1/1993 | EPI_ISL_30895 | | | EPI239733 | | |  |  |  |
| A/equine/Newmarket/2/1993 | EPI_ISL_30896 | | | EPI239739 | | |  |  |  |
| A/equine/Newmarket/5/2003 | EPI_ISL_30894 | | | EPI235161 | | |  |  |  |
| A/equine/Northamptonshire/1/2013 | EPI_ISL_151841 | | | EPI584252 | | |  |  |  |
| A/equine/Ohio/1/2003 | EPI_ISL_9534 | | | EPI98541 | | |  |  |  |
| A/equine/Ohio/113461-1/2005 | EPI_ISL_79869 | | | EPI281356 | | |  |  |  |
| A/equine/Ohio/113461-2/2005 | EPI_ISL_79866 | | | EPI281332 | | |  |  |  |
| A/equine/Ohio/113461-2/2005 | EPI_ISL_79866 | | | EPI281332 | | |  |  |  |
| A/equine/Ohio/113461-3/2005 | EPI_ISL_79870 | | | EPI281364 | | |  |  |  |
| A/equine/OR/78356/2012 | EPI_ISL_167703 | | | EPI546856 | | |  |  |  |
| A/equine/Oregon/78356/2012 | EPI_ISL_294934 | | | EPI1157851 | | |  |  |  |
| A/equine/Perthshire/3/09 | EPI_ISL_177492 | | | EPI584212 | | |  |  |  |
| A/equine/Qinghai/1/1994 | EPI_ISL_32661 | | | EPI186895 | | |  |  |  |
| A/equine/Richmond/1/2007 | EPI_ISL_153499 | | | EPI501398 | | |  |  |  |
| A/equine/Rio Grande do Sul/1/12 | EPI_ISL_177502 | | | EPI584296 | | |  |  |  |
| A/equine/Roma/5/1991 | EPI_ISL_19677 | | | EPI153973 | | |  |  |  |
| A/equine/Romania/1/1980 | EPI_ISL_19680 | | | EPI153997 | | |  |  |  |
| A/equine/Sachiyama/1/1971 | EPI_ISL_25008 | | | EPI165404 | | |  |  |  |
| A/equine/Santa Fe/1/1985 | EPI_ISL_19672 | | | EPI153933 | | |  |  |  |
| A/equine/Santiago/1/1985 | EPI_ISL_3674 | | | EPI19626 | | |  |  |  |
| A/equine/Sao Paulo/1.19/2012 | EPI_ISL_279065 | | | EPI1065026 | | |  |  |  |
| A/equine/Sao Paulo/1/1969 | EPI_ISL_19681 | | | EPI154005 | | |  |  |  |
| A/equine/Sao Paulo/6/1963 | EPI_ISL_19670 | | | EPI153917 | | |  |  |  |
| A/equine/Saone-et-Loire/1/2015 | EPI_ISL_257683 | | | EPI969029 | | |  |  |  |
| A/equine/Shropshire/10 | EPI_ISL_156748 | | | EPI584228 | | |  |  |  |
| A/equine/Snailwell/98 | EPI_ISL_177489 | | | EPI584188 | | |  |  |  |
| A/Equine/South Africa/4/03 | EPI_ISL_240665 | | | EPI873605 | | |  |  |  |
| A/equine/South Kazakhstan/236/2012 | EPI_ISL_166607 | | | EPI542621 | | |  |  |  |
| A/Equine/Stirlingshire/1/16 | EPI_ISL_255633 | | | EPI957637 | | |  |  |  |
| A/equine/Sussex/1/1989 | EPI_ISL_19884 | | | EPI240129 | | |  |  |  |
| A/equine/Sussex/1/1989 | EPI_ISL_19884 | | | EPI240129 | | |  |  |  |
| A/equine/Sussex/93753/89 | EPI_ISL_8280 | | | EPI154463 | | |  |  |  |
| A/Equine/Sweden/SVA111206SZ0085/VIR165837/2011 | EPI_ISL_180684 | | | EPI594033 | | |  |  |  |
| A/equine/Switzerland/1118/1979 | EPI_ISL_19679 | | | EPI153989 | | |  |  |  |
| A/equine/Switzerland/173/1993 | EPI_ISL_19676 | | | EPI153965 | | |  |  |  |
| A/equine/Switzerland/2225/1979 | EPI_ISL_22611 | | | EPI159275 | | |  |  |  |
| A/equine/Switzerland/P112/07 | EPI_ISL_177468 | | | EPI584117 | | |  |  |  |
| A/equine/Tennessee/27A/2014 | EPI_ISL_220483 | | | EPI753445 | | |  |  |  |
| A/equine/Tennessee/28A/2014 | EPI_ISL_220480 | | | EPI753426 | | |  |  |  |
| A/equine/Tennessee/28B/2014 | EPI_ISL_220484 | | | EPI753471 | | |  |  |  |
| A/equine/Tennessee/29A/2014 | EPI_ISL_220485 | | | EPI753458 | | |  |  |  |
| A/equine/Tennessee/30A/2014 | EPI_ISL_220482 | | | EPI753469 | | |  |  |  |
| A/equine/Tennessee/4A/2014 | EPI_ISL_220481 | | | EPI753461 | | |  |  |  |
| A/equine/Tennessee/5/1986 | EPI_ISL_21061 | | | EPI133244 | | |  |  |  |
| A/equine/Tennessee/5/1986 | EPI_ISL_21061 | | | EPI133244 | | |  |  |  |
| A/equine/Texas/117793/2005 | EPI_ISL_79867 | | | EPI281340 | | |  |  |  |
| A/equine/Texas/117793/2005 | EPI_ISL_79867 | | | EPI281340 | | |  |  |  |
| A/equine/Texas/39655/1991 | EPI_ISL_15349 | | | EPI133187 | | |  |  |  |
| A/equine/Tokyo/2/1971 | EPI_ISL_95197 | | | EPI332912 | | |  |  |  |
| A/equine/Tottori/1/07 | EPI_ISL_89221 | | | EPI314451 | | |  |  |  |
| A/equine/Uruguay/1/1963 | EPI_ISL_21282 | | | EPI154029 | | |  |  |  |
| A/equine/Virginia/131054-3/2005 | EPI_ISL_79899 | | | EPI281596 | | |  |  |  |
| A/equine/Wisconsin/1/03 | EPI_ISL_9783 | | | EPI99380 | | |  |  |  |
| A/equine/Xinjiang/1/2007 | EPI_ISL_32662 | | | EPI186903 | | |  |  |  |
| A/equine/Xinjiang/2/2007 | EPI_ISL_32663 | | | EPI186911 | | |  |  |  |
| A/equine/Xinjiang/3/2007 | EPI_ISL_32664 | | | EPI186919 | | |  |  |  |
| A/equine/Xinjiang/4/2007 | EPI_ISL_32665 | | | EPI186927 | | |  |  |  |
| A/equine/Xinjiang/5/2007 | EPI_ISL_32666 | | | EPI186935 | | |  |  |  |
| A/equine/Xuzhou/01/2013 | EPI_ISL_151130 | | | EPI489633 | | |  |  |  |
| A/equine/Yokohama/aq13/2010 | EPI_ISL_297955 | | | EPI1172955 | | |  |  |  |
| A/equine/Yorkshire/3/09 | EPI_ISL_156745 | | | EPI584220 | | |  |  |  |
| A /Equine/Sweden/SVA111128SZ0073/VIR160172/2011 | EPI_ISL_180755 | | | EPI594745 | | |  |  |  |
| NS1 | | | | | | |  |  |  |
| Isolate_Name | Isolate_Id | | | Segment_Id | | |  |  |  |
| A/canine/California/70645-4/2006 | EPI_ISL_79878 | | | EPI281431 | | |  |  |  |
| A/canine/Colorado/17864/2006 | EPI_ISL_79875 | | | EPI281407 | | |  |  |  |
| A/canine/Colorado/17864/2006 | EPI_ISL_79875 | | | EPI281407 | | |  |  |  |
| A/canine/Colorado/30604/2006 | EPI_ISL_79877 | | | EPI281423 | | |  |  |  |
| A/canine/Colorado/6723-14/2008 | EPI_ISL_82077 | | | EPI289223 | | |  |  |  |
| A/canine/Colorado/6723-8/2008 | EPI_ISL_79901 | | | EPI281615 | | |  |  |  |
| A/canine/Colorado/6723-8/2008 | EPI_ISL_79901 | | | EPI281615 | | |  |  |  |
| A/canine/Colorado/8880/2006 | EPI_ISL_82095 | | | EPI289222 | | |  |  |  |
| A/canine/Colorado/8880/2006 | EPI_ISL_82095 | | | EPI289222 | | |  |  |  |
| A/canine/Connecticut/85863/2011 | EPI_ISL_294935 | | | EPI1157880 | | |  |  |  |
| A/canine/Florida/15592.1/2004 | EPI_ISL_294945 | | | EPI1157875 | | |  |  |  |
| A/canine/Florida/242/2003 | EPI_ISL_9528 | | | EPI98492 | | |  |  |  |
| A/canine/Florida/43/2004 | EPI_ISL_9527 | | | EPI98474 | | |  |  |  |
| A/canine/Florida/61156-2/2006 | EPI_ISL_79895 | | | EPI281567 | | |  |  |  |
| A/canine/Florida/61156-2/2006 | EPI_ISL_79895 | | | EPI281567 | | |  |  |  |
| A/canine/Florida/78592-2/2006 | EPI_ISL_79879 | | | EPI281439 | | |  |  |  |
| A/canine/Florida/78592-6/2006 | EPI_ISL_79880 | | | EPI281447 | | |  |  |  |
| A/canine/Florida/78592-7/2006 | EPI_ISL_79881 | | | EPI281455 | | |  |  |  |
| A/canine/Florida/89911-2/2006 | EPI_ISL_79882 | | | EPI281463 | | |  |  |  |
| A/canine/Jacksonville/2005 | EPI_ISL_64818 | | | EPI226912 | | |  |  |  |
| A/canine/Kentucky/118778/2006 | EPI_ISL_79889 | | | EPI281519 | | |  |  |  |
| A/canine/Maine/058124/2016 | EPI_ISL_294942 | | | EPI1157838 | | |  |  |  |
| A/canine/Massachusetts/26810/2016 | EPI_ISL_234612 | | | EPI839114 | | |  |  |  |
| A/canine/Miami/2005 | EPI_ISL_64819 | | | EPI226918 | | |  |  |  |
| A/canine/New York/100525-1/2006 | EPI_ISL_79883 | | | EPI281471 | | |  |  |  |
| A/canine/New York/100528-1/2006 | EPI_ISL_79884 | | | EPI281479 | | |  |  |  |
| A/canine/New York/100528-5/2006 | EPI_ISL_79885 | | | EPI281487 | | |  |  |  |
| A/canine/New York/100528-6/2006 | EPI_ISL_79886 | | | EPI281495 | | |  |  |  |
| A/canine/New York/115719/2007 | EPI_ISL_79896 | | | EPI281575 | | |  |  |  |
| A/canine/New York/115809/2005 | EPI_ISL_79864 | | | EPI281319 | | |  |  |  |
| A/canine/New York/145353/2008 | EPI_ISL_79904 | | | EPI281639 | | |  |  |  |
| A/canine/New York/147926-3/2006 | EPI_ISL_79887 | | | EPI281503 | | |  |  |  |
| A/canine/New York/147926-5/2006 | EPI_ISL_79888 | | | EPI281511 | | |  |  |  |
| A/canine/New York/158402-1/2008 | EPI_ISL_79905 | | | EPI281647 | | |  |  |  |
| A/canine/New York/159903/2012 | EPI_ISL_294938 | | | EPI1157846 | | |  |  |  |
| A/canine/New York/1623.1/2010 | EPI_ISL_294943 | | | EPI1157870 | | |  |  |  |
| A/canine/New York/3699/2010 | EPI_ISL_294940 | | | EPI1157863 | | |  |  |  |
| A/canine/New York/4986-2/2006 | EPI_ISL_79873 | | | EPI281391 | | |  |  |  |
| A/canine/New York/5183-6/2006 | EPI_ISL_79871 | | | EPI281375 | | |  |  |  |
| A/canine/New York/5183-6/2006 | EPI_ISL_79871 | | | EPI281375 | | |  |  |  |
| A/canine/New York/51854/2008 | EPI_ISL_79902 | | | EPI281623 | | |  |  |  |
| A/canine/NY/100525/2006 | EPI_ISL_81918 | | | EPI289227 | | |  |  |  |
| A/canine/NY/100528-4/2006 | EPI_ISL_81920 | | | EPI289225 | | |  |  |  |
| A/canine/NY/105447/2008 | EPI_ISL_174477 | | | EPI576532 | | |  |  |  |
| A/canine/NY/120106.2/2011 | EPI_ISL_166503 | | | EPI546861 | | |  |  |  |
| A/canine/NY/147926/2006 | EPI_ISL_82076 | | | EPI289228 | | |  |  |  |
| A/canine/NY/4986/2006 | EPI_ISL_81919 | | | EPI289229 | | |  |  |  |
| A/canine/NY/dog1c02/2009 | EPI_ISL_82031 | | | EPI289333 | | |  |  |  |
| A/canine/NY/dog1c04/2009 | EPI_ISL_82029 | | | EPI289332 | | |  |  |  |
| A/canine/NY/dog1c05/2009 | EPI_ISL_82036 | | | EPI289334 | | |  |  |  |
| A/canine/NY/dog1c06/2009 | EPI_ISL_82030 | | | EPI289335 | | |  |  |  |
| A/canine/NY/dog1c09/2009 | EPI_ISL_82032 | | | EPI289336 | | |  |  |  |
| A/canine/NY/dog1c11/2009 | EPI_ISL_82022 | | | EPI289337 | | |  |  |  |
| A/canine/NY/dog1c18/2009 | EPI_ISL_82027 | | | EPI289338 | | |  |  |  |
| A/canine/NY/dog1c20/2009 | EPI_ISL_82060 | | | EPI289339 | | |  |  |  |
| A/canine/NY/dog1c26/2009 | EPI_ISL_82130 | | | EPI289314 | | |  |  |  |
| A/canine/NY/dog1c35/2009 | EPI_ISL_82129 | | | EPI289313 | | |  |  |  |
| A/canine/NY/dog1c38/2009 | EPI_ISL_82126 | | | EPI289310 | | |  |  |  |
| A/canine/NY/dog1c40/2009 | EPI_ISL_82125 | | | EPI289309 | | |  |  |  |
| A/canine/NY/dog1c41/2009 | EPI_ISL_82128 | | | EPI289312 | | |  |  |  |
| A/canine/NY/dog1c42/2009 | EPI_ISL_82127 | | | EPI289311 | | |  |  |  |
| A/canine/NY/dog21c02/2009 | EPI_ISL_82102 | | | EPI289238 | | |  |  |  |
| A/canine/NY/dog21c08/2009 | EPI_ISL_81929 | | | EPI289240 | | |  |  |  |
| A/canine/NY/dog21c14/2009 | EPI_ISL_81922 | | | EPI289237 | | |  |  |  |
| A/canine/NY/dog21c21/2009 | EPI_ISL_82103 | | | EPI289239 | | |  |  |  |
| A/canine/NY/dog23c01/2009 | EPI_ISL_82097 | | | EPI289232 | | |  |  |  |
| A/canine/NY/dog23c02/2009 | EPI_ISL_82096 | | | EPI289231 | | |  |  |  |
| A/canine/NY/dog23c08/2009 | EPI_ISL_82099 | | | EPI289234 | | |  |  |  |
| A/canine/NY/dog23c13/2009 | EPI_ISL_82101 | | | EPI289236 | | |  |  |  |
| A/canine/NY/dog23c15/2009 | EPI_ISL_82100 | | | EPI289235 | | |  |  |  |
| A/canine/NY/dog23c17/2009 | EPI_ISL_82098 | | | EPI289233 | | |  |  |  |
| A/canine/NY/dog2c01/2009 | EPI_ISL_82042 | | | EPI289350 | | |  |  |  |
| A/canine/NY/dog2c02/2009 | EPI_ISL_82041 | | | EPI289303 | | |  |  |  |
| A/canine/NY/dog2c04/2009 | EPI_ISL_82040 | | | EPI289351 | | |  |  |  |
| A/canine/NY/dog2c05/2009 | EPI_ISL_82039 | | | EPI289304 | | |  |  |  |
| A/canine/NY/dog2c06/2009 | EPI_ISL_82046 | | | EPI289352 | | |  |  |  |
| A/canine/NY/dog2c07/2009 | EPI_ISL_82134 | | | EPI289353 | | |  |  |  |
| A/canine/NY/dog2c10/2009 | EPI_ISL_82049 | | | EPI289305 | | |  |  |  |
| A/canine/NY/dog2c14/2009 | EPI_ISL_82047 | | | EPI289302 | | |  |  |  |
| A/canine/NY/dog2c15/2009 | EPI_ISL_82123 | | | EPI289306 | | |  |  |  |
| A/canine/NY/dog2c16/2009 | EPI_ISL_82135 | | | EPI289354 | | |  |  |  |
| A/canine/NY/dog2c17/2009 | EPI_ISL_82043 | | | EPI289307 | | |  |  |  |
| A/canine/NY/dog2c18/2009 | EPI_ISL_82124 | | | EPI289308 | | |  |  |  |
| A/canine/NY/dog2c19/2009 | EPI_ISL_82136 | | | EPI289355 | | |  |  |  |
| A/canine/NY/dog2c20/2009 | EPI_ISL_82045 | | | EPI289356 | | |  |  |  |
| A/canine/NY/dog3c01/2009 | EPI_ISL_82138 | | | EPI289358 | | |  |  |  |
| A/canine/NY/dog3c03/2009 | EPI_ISL_82137 | | | EPI289357 | | |  |  |  |
| A/canine/NY/dog3c04/2009 | EPI_ISL_82051 | | | EPI289360 | | |  |  |  |
| A/canine/NY/dog3c05/2009 | EPI_ISL_82139 | | | EPI289359 | | |  |  |  |
| A/canine/NY/dog3c06/2009 | EPI_ISL_82140 | | | EPI289361 | | |  |  |  |
| A/canine/NY/dog3c07/2009 | EPI_ISL_82141 | | | EPI289362 | | |  |  |  |
| A/canine/NY/dog3c09/2009 | EPI_ISL_82056 | | | EPI289372 | | |  |  |  |
| A/canine/NY/dog3c10/2009 | EPI_ISL_82142 | | | EPI289363 | | |  |  |  |
| A/canine/NY/dog3c11/2009 | EPI_ISL_82055 | | | EPI289364 | | |  |  |  |
| A/canine/NY/dog3c12/2009 | EPI_ISL_82014 | | | EPI289365 | | |  |  |  |
| A/canine/NY/dog3c13/2009 | EPI_ISL_82143 | | | EPI289366 | | |  |  |  |
| A/canine/NY/dog3c14/2009 | EPI_ISL_82147 | | | EPI289373 | | |  |  |  |
| A/canine/NY/dog3c15/2009 | EPI_ISL_82144 | | | EPI289367 | | |  |  |  |
| A/canine/NY/dog3c17/2009 | EPI_ISL_82145 | | | EPI289368 | | |  |  |  |
| A/canine/NY/dog3c18/2009 | EPI_ISL_82146 | | | EPI289369 | | |  |  |  |
| A/canine/NY/dog3c19/2009 | EPI_ISL_82015 | | | EPI289370 | | |  |  |  |
| A/canine/NY/dog3c20/2009 | EPI_ISL_82016 | | | EPI289371 | | |  |  |  |
| A/canine/NY/dog4c01/2009 | EPI_ISL_82013 | | | EPI289342 | | |  |  |  |
| A/canine/NY/dog4c02/2009 | EPI_ISL_82011 | | | EPI289343 | | |  |  |  |
| A/canine/NY/dog4c03/2009 | EPI_ISL_82010 | | | EPI289296 | | |  |  |  |
| A/canine/NY/dog4c05/2009 | EPI_ISL_82122 | | | EPI289297 | | |  |  |  |
| A/canine/NY/dog4c07/2009 | EPI_ISL_82085 | | | EPI289344 | | |  |  |  |
| A/canine/NY/dog4c08/2009 | EPI_ISL_82007 | | | EPI289298 | | |  |  |  |
| A/canine/NY/dog4c09/2009 | EPI_ISL_82131 | | | EPI289345 | | |  |  |  |
| A/canine/NY/dog4c10/2009 | EPI_ISL_82132 | | | EPI289346 | | |  |  |  |
| A/canine/NY/dog4c11/2009 | EPI_ISL_82073 | | | EPI289299 | | |  |  |  |
| A/canine/NY/dog4c12/2009 | EPI_ISL_82000 | | | EPI289347 | | |  |  |  |
| A/canine/NY/dog4c14/2009 | EPI_ISL_82133 | | | EPI289348 | | |  |  |  |
| A/canine/NY/dog4c15/2009 | EPI_ISL_82003 | | | EPI289300 | | |  |  |  |
| A/canine/NY/dog4c18/2009 | EPI_ISL_82086 | | | EPI289349 | | |  |  |  |
| A/canine/NY/dog4c19/2009 | EPI_ISL_82087 | | | EPI289301 | | |  |  |  |
| A/canine/NY/dog5c01/2009 | EPI_ISL_82074 | | | EPI289316 | | |  |  |  |
| A/canine/NY/dog5c03/2009 | EPI_ISL_82052 | | | EPI289317 | | |  |  |  |
| A/canine/NY/dog5c04/2009 | EPI_ISL_82062 | | | EPI289318 | | |  |  |  |
| A/canine/NY/dog5c06/2009 | EPI_ISL_82019 | | | EPI289319 | | |  |  |  |
| A/canine/NY/dog5c07/2009 | EPI_ISL_82018 | | | EPI289320 | | |  |  |  |
| A/canine/NY/dog5c09/2009 | EPI_ISL_82064 | | | EPI289321 | | |  |  |  |
| A/canine/NY/dog5c10/2009 | EPI_ISL_82017 | | | EPI289322 | | |  |  |  |
| A/canine/NY/dog5c11/2009 | EPI_ISL_82020 | | | EPI289323 | | |  |  |  |
| A/canine/NY/dog5c12/2009 | EPI_ISL_82065 | | | EPI289315 | | |  |  |  |
| A/canine/NY/dog5c13/2009 | EPI_ISL_82066 | | | EPI289324 | | |  |  |  |
| A/canine/NY/dog5c14/2009 | EPI_ISL_82067 | | | EPI289325 | | |  |  |  |
| A/canine/NY/dog5c15/2009 | EPI_ISL_82068 | | | EPI289326 | | |  |  |  |
| A/canine/NY/dog5c16/2009 | EPI_ISL_82069 | | | EPI289327 | | |  |  |  |
| A/canine/NY/dog5c17/2009 | EPI_ISL_82070 | | | EPI289328 | | |  |  |  |
| A/canine/NY/dog5c18/2009 | EPI_ISL_82071 | | | EPI289329 | | |  |  |  |
| A/canine/NY/dog5c19/2009 | EPI_ISL_82021 | | | EPI289330 | | |  |  |  |
| A/canine/NY/dog5c20/2009 | EPI_ISL_82072 | | | EPI289331 | | |  |  |  |
| A/canine/NY/dog6c03/2009 | EPI_ISL_82008 | | | EPI289294 | | |  |  |  |
| A/canine/NY/dog6c06/2009 | EPI_ISL_82090 | | | EPI289340 | | |  |  |  |
| A/canine/NY/dog6c07/2009 | EPI_ISL_82001 | | | EPI289295 | | |  |  |  |
| A/canine/NY/dog6c16/2009 | EPI_ISL_82084 | | | EPI289341 | | |  |  |  |
| A/canine/NY/dog6c22/2009 | EPI_ISL_81935 | | | EPI289289 | | |  |  |  |
| A/canine/NY/dog6c23/2009 | EPI_ISL_82118 | | | EPI289290 | | |  |  |  |
| A/canine/NY/dog6c25/2009 | EPI_ISL_82119 | | | EPI289291 | | |  |  |  |
| A/canine/NY/dog6c27/2009 | EPI_ISL_82120 | | | EPI289292 | | |  |  |  |
| A/canine/NY/dog6c28/2009 | EPI_ISL_82121 | | | EPI289293 | | |  |  |  |
| A/canine/NY/dog7c01/2008 | EPI_ISL_81985 | | | EPI289274 | | |  |  |  |
| A/canine/NY/dog7c02/2008 | EPI_ISL_81984 | | | EPI289275 | | |  |  |  |
| A/canine/NY/dog7c03/2008 | EPI_ISL_81954 | | | EPI289276 | | |  |  |  |
| A/canine/NY/dog7c04/2008 | EPI_ISL_81955 | | | EPI289277 | | |  |  |  |
| A/canine/NY/dog7c05/2008 | EPI_ISL_81986 | | | EPI289278 | | |  |  |  |
| A/canine/NY/dog7c07/2008 | EPI_ISL_81956 | | | EPI289279 | | |  |  |  |
| A/canine/NY/dog7c08/2008 | EPI_ISL_82117 | | | EPI289280 | | |  |  |  |
| A/canine/NY/dog7c09/2008 | EPI_ISL_81987 | | | EPI289241 | | |  |  |  |
| A/canine/NY/dog7c11/2008 | EPI_ISL_81988 | | | EPI289281 | | |  |  |  |
| A/canine/NY/dog7c12/2008 | EPI_ISL_81989 | | | EPI289282 | | |  |  |  |
| A/canine/NY/dog7c13/2008 | EPI_ISL_81990 | | | EPI289283 | | |  |  |  |
| A/canine/NY/dog7c14/2008 | EPI_ISL_81991 | | | EPI289284 | | |  |  |  |
| A/canine/NY/dog7c15/2008 | EPI_ISL_81992 | | | EPI289285 | | |  |  |  |
| A/canine/NY/dog7c16/2008 | EPI_ISL_81993 | | | EPI289286 | | |  |  |  |
| A/canine/NY/dog7c17/2008 | EPI_ISL_81994 | | | EPI289287 | | |  |  |  |
| A/canine/NY/dog7c19/2008 | EPI_ISL_81995 | | | EPI289288 | | |  |  |  |
| A/canine/NY/dog8c01/2008 | EPI_ISL_81969 | | | EPI289258 | | |  |  |  |
| A/canine/NY/dog8c02/2008 | EPI_ISL_81970 | | | EPI289259 | | |  |  |  |
| A/canine/NY/dog8c03/2008 | EPI_ISL_81971 | | | EPI289257 | | |  |  |  |
| A/canine/NY/dog8c04/2008 | EPI_ISL_82112 | | | EPI289260 | | |  |  |  |
| A/canine/NY/dog8c05/2008 | EPI_ISL_81981 | | | EPI289261 | | |  |  |  |
| A/canine/NY/dog8c06/2008 | EPI_ISL_81972 | | | EPI289262 | | |  |  |  |
| A/canine/NY/dog8c07/2008 | EPI_ISL_81973 | | | EPI289263 | | |  |  |  |
| A/canine/NY/dog8c08/2008 | EPI_ISL_82113 | | | EPI289264 | | |  |  |  |
| A/canine/NY/dog8c10/2008 | EPI_ISL_81975 | | | EPI289265 | | |  |  |  |
| A/canine/NY/dog8c11/2008 | EPI_ISL_82114 | | | EPI289266 | | |  |  |  |
| A/canine/NY/dog8c12/2008 | EPI_ISL_81982 | | | EPI289267 | | |  |  |  |
| A/canine/NY/dog8c13/2008 | EPI_ISL_81976 | | | EPI289268 | | |  |  |  |
| A/canine/NY/dog8c14/2008 | EPI_ISL_81977 | | | EPI289269 | | |  |  |  |
| A/canine/NY/dog8c15/2008 | EPI_ISL_81978 | | | EPI289270 | | |  |  |  |
| A/canine/NY/dog8c16/2008 | EPI_ISL_81980 | | | EPI289271 | | |  |  |  |
| A/canine/NY/dog8c17/2008 | EPI_ISL_82115 | | | EPI289272 | | |  |  |  |
| A/canine/NY/dog8c18/2008 | EPI_ISL_82116 | | | EPI289273 | | |  |  |  |
| A/canine/NY/dog9c01/2008 | EPI_ISL_82104 | | | EPI289242 | | |  |  |  |
| A/canine/NY/dog9c02/2008 | EPI_ISL_82105 | | | EPI289243 | | |  |  |  |
| A/canine/NY/dog9c03/2008 | EPI_ISL_81960 | | | EPI289244 | | |  |  |  |
| A/canine/NY/dog9c07/2008 | EPI_ISL_81961 | | | EPI289245 | | |  |  |  |
| A/canine/NY/dog9c08/2008 | EPI_ISL_81962 | | | EPI289246 | | |  |  |  |
| A/canine/NY/dog9c09/2008 | EPI_ISL_81963 | | | EPI289247 | | |  |  |  |
| A/canine/NY/dog9c10/2008 | EPI_ISL_82106 | | | EPI289248 | | |  |  |  |
| A/canine/NY/dog9c11/2008 | EPI_ISL_82107 | | | EPI289249 | | |  |  |  |
| A/canine/NY/dog9c13/2008 | EPI_ISL_82108 | | | EPI289250 | | |  |  |  |
| A/canine/NY/dog9c14/2008 | EPI_ISL_82109 | | | EPI289251 | | |  |  |  |
| A/canine/NY/dog9c15/2008 | EPI_ISL_81965 | | | EPI289252 | | |  |  |  |
| A/canine/NY/dog9c16/2008 | EPI_ISL_81966 | | | EPI289253 | | |  |  |  |
| A/canine/NY/dog9c17/2008 | EPI_ISL_82110 | | | EPI289254 | | |  |  |  |
| A/canine/NY/dog9c19/2008 | EPI_ISL_82111 | | | EPI289255 | | |  |  |  |
| A/canine/NY/dog9c20/2008 | EPI_ISL_81968 | | | EPI289256 | | |  |  |  |
| A/canine/PA/111788.2/2009 | EPI_ISL_166506 | | | EPI546864 | | |  |  |  |
| A/canine/PA/33225.4/2010 | EPI_ISL_167696 | | | EPI546862 | | |  |  |  |
| A/canine/Pennsylvania/10909/2007 | EPI_ISL_79891 | | | EPI281535 | | |  |  |  |
| A/canine/Pennsylvania/10915/2007 | EPI_ISL_79892 | | | EPI281543 | | |  |  |  |
| A/canine/Pennsylvania/137154/2008 | EPI_ISL_79903 | | | EPI281631 | | |  |  |  |
| A/canine/Pennsylvania/16699/2007 | EPI_ISL_79890 | | | EPI281527 | | |  |  |  |
| A/canine/Pennsylvania/94930-3/2007 | EPI_ISL_79897 | | | EPI281583 | | |  |  |  |
| A/canine/Pennsylvania/96978/2009 | EPI_ISL_294937 | | | EPI1157808 | | |  |  |  |
| A/canine/Philadelphia/6371100/2008 | EPI_ISL_82057 | | | EPI289226 | | |  |  |  |
| A/canine/StatenIs/115719/2007 | EPI_ISL_81921 | | | EPI289224 | | |  |  |  |
| A/canine/Vermont/278213/2013 | EPI_ISL_294941 | | | EPI1157876 | | |  |  |  |
| A/canine/Virginia/93653/2009 | EPI_ISL_82078 | | | EPI289230 | | |  |  |  |
| A/canine/VT/11039/2013 | EPI_ISL_166505 | | | EPI546863 | | |  |  |  |
| A/donkey/Shandong/1/2017 | EPI_ISL_284681 | | | EPI1098484 | | |  |  |  |
| A/eq/Hong Kong/1/92 | EPI_ISL_683 | | | EPI3578 | | |  |  |  |
| A/eq/Kentucky/1/88 | EPI_ISL_677 | | | EPI3560 | | |  |  |  |
| A/eq/Kentucky/92 | EPI_ISL_68829 | | | EPI239791 | | |  |  |  |
| A/eq/LaPlata/1/88 | EPI_ISL_678 | | | EPI3563 | | |  |  |  |
| A/eq/LaPlata/93 | EPI_ISL_68814 | | | EPI239728 | | |  |  |  |
| A/eq/Newmarket/D63/79 | EPI_ISL_675 | | | EPI3554 | | |  |  |  |
| A/eq/Roma/5/91 | EPI_ISL_682 | | | EPI3575 | | |  |  |  |
| A/equi-2/Ludhiana/87 | EPI_ISL_68955 | | | EPI240108 | | |  |  |  |
| A/equine/Aboyne/1/05 | EPI_ISL_11913 | | | EPI177926 | | |  |  |  |
| A/equine/Ahmedabad/1/2009 | EPI_ISL_74364 | | | EPI255567 | | |  |  |  |
| A/Equine/Alaska/1/91 | EPI_ISL_680 | | | EPI3569 | | |  |  |  |
| A/equine/Alaska/29759/1991 | EPI_ISL_15351 | | | EPI133227 | | |  |  |  |
| A/equine/Algiers/1/1972 | EPI_ISL_20808 | | | EPI156253 | | |  |  |  |
| A/equine/Almaty/26/2007 | EPI_ISL_71977 | | | EPI250069 | | |  |  |  |
| A/equine/Ankara/1/2013 | EPI_ISL_281349 | | | EPI1076910 | | |  |  |  |
| A/equine/Arundel/12369/91 | EPI_ISL_681 | | | EPI3572 | | |  |  |  |
| A/equine/Athens/02/2003 | EPI_ISL_75320 | | | EPI347185 | | |  |  |  |
| A/equine/Athens/04/2007 | EPI_ISL_28012 | | | EPI347186 | | |  |  |  |
| A/equine/Austria/421/1992 | EPI_ISL_19675 | | | EPI153958 | | |  |  |  |
| A/equine/Avesta/1993 | EPI_ISL_68576 | | | EPI238719 | | |  |  |  |
| A/Equine/Ayrshire/1/2013 | EPI_ISL_240595 | | | EPI873572 | | |  |  |  |
| A/equine/Baizak/09/2012 | EPI_ISL_170787 | | | EPI558703 | | |  |  |  |
| A/equine/Belfond/6-2/2009 | EPI_ISL_257677 | | | EPI969065 | | |  |  |  |
| A/equine/Berlin/1/1989 | EPI_ISL_19683 | | | EPI154022 | | |  |  |  |
| A/equine/Buckinghamshire/1/2014 | EPI_ISL_197753 | | | EPI651402 | | |  |  |  |
| A/equine/California/1/10 | EPI_ISL_177498 | | | EPI584274 | | |  |  |  |
| A/equine/California/1/1980 | EPI_ISL_8089 | | | EPI77757 | | |  |  |  |
| A/equine/California/1/2007 | EPI_ISL_29840 | | | EPI177911 | | |  |  |  |
| A/equine/California/103/1982 | EPI_ISL_8101 | | | EPI77985 | | |  |  |  |
| A/equine/California/2/2007 | EPI_ISL_29835 | | | EPI177912 | | |  |  |  |
| A/equine/California/4537/1997 | EPI_ISL_15842 | | | EPI137765 | | |  |  |  |
| A/equine/California/83/1982 | EPI_ISL_15343 | | | EPI133056 | | |  |  |  |
| A/equine/California/8560/2002 | EPI_ISL_15843 | | | EPI137784 | | |  |  |  |
| A/equine/Cambremer/1/2012 | EPI_ISL_257680 | | | EPI969067 | | |  |  |  |
| A/equine/Cheshire/06 | EPI_ISL_177472 | | | EPI584153 | | |  |  |  |
| A/equine/Cheshire/1/2006 | EPI_ISL_29834 | | | EPI177903 | | |  |  |  |
| A/equine/Cheshire/1/2007 | EPI_ISL_29843 | | | EPI177923 | | |  |  |  |
| A/equine/Cheshire/2/2007 | EPI_ISL_29825 | | | EPI177905 | | |  |  |  |
| A/equine/Cheshire/3/2007 | EPI_ISL_29826 | | | EPI177906 | | |  |  |  |
| A/equine/Cordoba/18/1985 | EPI_ISL_19671 | | | EPI153926 | | |  |  |  |
| A/equine/Devon/1/11 | EPI_ISL_156749 | | | EPI584248 | | |  |  |  |
| A/equine/Dorset/09 | EPI_ISL_177491 | | | EPI584207 | | |  |  |  |
| A/equine/Dubai/1/12 | EPI_ISL_177501 | | | EPI584290 | | |  |  |  |
| A/equine/East Renfrewshire/2/11 | EPI_ISL_156750 | | | EPI584240 | | |  |  |  |
| A/Equine/East Sussex/1/2015 | EPI_ISL_240662 | | | EPI873581 | | |  |  |  |
| A/equine/Essaouira/2/2004 | EPI_ISL_119932 | | | EPI372055 | | |  |  |  |
| A/equine/Essaouira/3/2004 | EPI_ISL_119933 | | | EPI372058 | | |  |  |  |
| A/equine/Essex/1/05 | EPI_ISL_11910 | | | EPI177925 | | |  |  |  |
| A/equine/Florida/146609/2011 | EPI_ISL_294944 | | | EPI1157832 | | |  |  |  |
| A/equine/Florida/2/2006 | EPI_ISL_29836 | | | EPI177913 | | |  |  |  |
| A/equine/Fontainbleu/1/1979 | EPI_ISL_19682 | | | EPI154014 | | |  |  |  |
| A/equine/Fontainebleau/1979 | EPI_ISL_69897 | | | EPI242349 | | |  |  |  |
| A/equine/France/1/1967 | EPI_ISL_22612 | | | EPI159284 | | |  |  |  |
| A/equine/Gansu/7/2008 | EPI_ISL_32656 | | | EPI186858 | | |  |  |  |
| A/equine/Georgia/1/1981 | EPI_ISL_8095 | | | EPI77871 | | |  |  |  |
| A/equine/Georgia/10/1981 | EPI_ISL_8098 | | | EPI77928 | | |  |  |  |
| A/equine/Georgia/121362-16/2016 | EPI_ISL_294939 | | | EPI1157817 | | |  |  |  |
| A/equine/Georgia/13/1981 | EPI_ISL_8099 | | | EPI77947 | | |  |  |  |
| A/equine/Georgia/3/1981 | EPI_ISL_8096 | | | EPI77890 | | |  |  |  |
| A/equine/Georgia/9/1981 | EPI_ISL_8097 | | | EPI77909 | | |  |  |  |
| A/equine/Gironde/1/2014 | EPI_ISL_257682 | | | EPI969068 | | |  |  |  |
| A/equine/Gopeswar/1/2009 | EPI_ISL_74365 | | | EPI255777 | | |  |  |  |
| A/equine/Grobois/1/98 | EPI_ISL_3541 | | | EPI18960 | | |  |  |  |
| A/equine/Guangxi/1/2008 | EPI_ISL_174545 | | | EPI573756 | | |  |  |  |
| A/Equine/Hampshire/4/2016 | EPI_ISL_240664 | | | EPI873597 | | |  |  |  |
| A/equine/Heilongjiang/1/2010 | EPI_ISL_144923 | | | EPI464998 | | |  |  |  |
| A/equine/Heilongjiang/10/2008 | EPI_ISL_32658 | | | EPI186874 | | |  |  |  |
| A/equine/Heilongjiang/SS1/2013 | EPI_ISL_161474 | | | EPI527597 | | |  |  |  |
| A/equine/Himachal Pradesh/CMVL-YOL2/2008 | EPI_ISL_190904 | | | EPI621747 | | |  |  |  |
| A/equine/Hissar/CMVL-HSR4/2008 | EPI_ISL_190901 | | | EPI621728 | | |  |  |  |
| A/equine/Hokkaido/I828/2008 | EPI_ISL_69119 | | | EPI240686 | | |  |  |  |
| A/equine/Horsham/1/2007 | EPI_ISL_29844 | | | EPI177901 | | |  |  |  |
| A/equine/Huabei/1/2007 | EPI_ISL_70195 | | | EPI243551 | | |  |  |  |
| A/equine/Hubei/6/2008 | EPI_ISL_32657 | | | EPI186866 | | |  |  |  |
| A/equine/Ibaraki/1/2007 | EPI_ISL_297956 | | | EPI1172966 | | |  |  |  |
| A/equine/Idaho/37875/1991 | EPI_ISL_6623 | | | EPI52076 | | |  |  |  |
| A/equine/Inner Mongolia/8/2008 | EPI_ISL_32660 | | | EPI186890 | | |  |  |  |
| A/equine/Italy/1062/1991 | EPI_ISL_19678 | | | EPI153982 | | |  |  |  |
| A/equine/Italy/1199/1992 | EPI_ISL_19674 | | | EPI153950 | | |  |  |  |
| A/equine/Italy/824/1991 | EPI_ISL_19673 | | | EPI153942 | | |  |  |  |
| A/equine/Jammu and Kashmir/CMVL-LEH4/2008 | EPI_ISL_190902 | | | EPI621734 | | |  |  |  |
| A/equine/Jammu and Kashmir/CMVL-LEH6/2008 | EPI_ISL_190903 | | | EPI621740 | | |  |  |  |
| A/equine/Johannesburg/1/1986 | EPI_ISL_20809 | | | EPI156261 | | |  |  |  |
| A/equine/Jouars/4/2006 | EPI_ISL_257684 | | | EPI969064 | | |  |  |  |
| A/equine/Kascakew/1/1978 | EPI_ISL_69898 | | | EPI242357 | | |  |  |  |
| A/equine/Katra-Jammu/7/2008 | EPI_ISL_74366 | | | EPI300731 | | |  |  |  |
| A/Equine/Kent/1/15 | EPI_ISL_240663 | | | EPI873589 | | |  |  |  |
| A/equine/Kentucky/1/11 | EPI_ISL_177500 | | | EPI584282 | | |  |  |  |
| A/equine/Kentucky/1/1978 | EPI_ISL_8087 | | | EPI77719 | | |  |  |  |
| A/equine/Kentucky/1/1981 | EPI_ISL_68957 | | | EPI240114 | | |  |  |  |
| A/equine/Kentucky/1/1986 | EPI_ISL_15344 | | | EPI133075 | | |  |  |  |
| A/equine/Kentucky/1/1987 | EPI_ISL_21060 | | | EPI133094 | | |  |  |  |
| A/equine/Kentucky/1/1991 | EPI_ISL_15352 | | | EPI133265 | | |  |  |  |
| A/equine/Kentucky/1/1992 | EPI_ISL_15350 | | | EPI133208 | | |  |  |  |
| A/equine/Kentucky/1/1995 | EPI_ISL_290255 | | | EPI1134106 | | |  |  |  |
| A/equine/Kentucky/1/1997 | EPI_ISL_29854 | | | EPI177927 | | |  |  |  |
| A/equine/Kentucky/1/1998 | EPI_ISL_29855 | | | EPI177928 | | |  |  |  |
| A/equine/Kentucky/1/1999 | EPI_ISL_290254 | | | EPI1134105 | | |  |  |  |
| A/Equine/Kentucky/1/2014 | EPI_ISL_206059 | | | EPI687837 | | |  |  |  |
| A/equine/Kentucky/1277/1990 | EPI_ISL_15348 | | | EPI133170 | | |  |  |  |
| A/equine/Kentucky/2/1980 | EPI_ISL_20807 | | | EPI156245 | | |  |  |  |
| A/equine/Kentucky/2/1981 | EPI_ISL_8090 | | | EPI77776 | | |  |  |  |
| A/equine/Kentucky/2/1986 | EPI_ISL_21280 | | | EPI153846 | | |  |  |  |
| A/equine/Kentucky/2/1987 | EPI_ISL_19104 | | | EPI152773 | | |  |  |  |
| A/equine/Kentucky/211/1987 | EPI_ISL_68958 | | | EPI240122 | | |  |  |  |
| A/equine/Kentucky/3/1981 | EPI_ISL_15342 | | | EPI133037 | | |  |  |  |
| A/equine/Kentucky/3/1986 | EPI_ISL_19663 | | | EPI153854 | | |  |  |  |
| A/equine/Kentucky/4/1980 | EPI_ISL_8088 | | | EPI77738 | | |  |  |  |
| A/equine/Kentucky/4/2007 | EPI_ISL_29837 | | | EPI177914 | | |  |  |  |
| A/equine/Kentucky/5/02 | EPI_ISL_4663 | | | EPI25750 | | |  |  |  |
| A/equine/Kentucky/5/2002 | EPI_ISL_9530 | | | EPI98543 | | |  |  |  |
| A/equine/Kentucky/692/1988 | EPI_ISL_15345 | | | EPI133113 | | |  |  |  |
| A/equine/Kentucky/694/1988 | EPI_ISL_15346 | | | EPI133132 | | |  |  |  |
| A/equine/Kentucky/698/1988 | EPI_ISL_15347 | | | EPI133151 | | |  |  |  |
| A/equine/Kentucky/7/2007 | EPI_ISL_29838 | | | EPI177915 | | |  |  |  |
| A/equine/Kentucky/8/1994 | EPI_ISL_15353 | | | EPI133284 | | |  |  |  |
| A/equine/Kentucky/9/2004 | EPI_ISL_29852 | | | EPI177924 | | |  |  |  |
| A/equine/Kentucky/bitter_boredom5/1976 | EPI_ISL_8094 | | | EPI77852 | | |  |  |  |
| A/equine/Kentucky/magnificent_genius1/1981 | EPI_ISL_8100 | | | EPI77966 | | |  |  |  |
| A/equine/Kentucky/pass_the_pepper1/1976 | EPI_ISL_8093 | | | EPI77833 | | |  |  |  |
| A/equine/Kentucky/Rosie100/1981 | EPI_ISL_15830 | | | EPI137537 | | |  |  |  |
| A/equine/Kostanay/09/2012 | EPI_ISL_170790 | | | EPI558706 | | |  |  |  |
| A/equine/Kyonggi/SA1/2011 | EPI_ISL_129971 | | | EPI398891 | | |  |  |  |
| A/equine/Lambourn/22778/92 | EPI_ISL_685 | | | EPI3584 | | |  |  |  |
| A/equine/Lanark/1/2006 | EPI_ISL_29833 | | | EPI177899 | | |  |  |  |
| A/equine/Lanarkshire/09 | EPI_ISL_177490 | | | EPI584199 | | |  |  |  |
| A/equine/LaPlata/1/1995 | EPI_ISL_290257 | | | EPI1134108 | | |  |  |  |
| A/equine/Liaoning/9/2008 | EPI_ISL_32659 | | | EPI186882 | | |  |  |  |
| A/equine/Lincolnshire/06 | EPI_ISL_177471 | | | EPI584131 | | |  |  |  |
| A/equine/Lincolnshire/1/2002 | EPI_ISL_29851 | | | EPI177929 | | |  |  |  |
| A/equine/Lincolnshire/1/2006 | EPI_ISL_29832 | | | EPI177900 | | |  |  |  |
| A/equine/Lincolnshire/1/2007 | EPI_ISL_29831 | | | EPI177907 | | |  |  |  |
| A/equine/LKZ/09/2012 | EPI_ISL_170788 | | | EPI558704 | | |  |  |  |
| A/equine/Maidstone/1/2007 | EPI_ISL_29846 | | | EPI177919 | | |  |  |  |
| A/equine/Maidstone/2/2007 | EPI_ISL_29845 | | | EPI177908 | | |  |  |  |
| A/equine/Malaysia/M201/2015 | EPI_ISL_201434 | | | EPI1073182 | | |  |  |  |
| A/equine/Massachussetts/213/2003 | EPI_ISL_9529 | | | EPI98512 | | |  |  |  |
| A/equine/Matybulak/10/2012 | EPI_ISL_170789 | | | EPI558705 | | |  |  |  |
| A/equine/Miami/1/1963 | EPI_ISL_8092 | | | EPI77814 | | |  |  |  |
| A/equine/Miami/1963 | EPI_ISL_161338 | | | EPI527215 | | |  |  |  |
| A/equine/Mongolia/1/2008 | EPI_ISL_19180 | | | EPI153117 | | |  |  |  |
| A/equine/Mongolia/3/2013 | EPI_ISL_290256 | | | EPI1134107 | | |  |  |  |
| A/equine/Montana/9233/2007 | EPI_ISL_79893 | | | EPI281551 | | |  |  |  |
| A/equine/Montana/9564-1/2015 | EPI_ISL_285417 | | | EPI1103412 | | |  |  |  |
| A/equine/Moulton/98 | EPI_ISL_177487 | | | EPI584183 | | |  |  |  |
| A/equine/Mysore/12/2008 | EPI_ISL_84310 | | | EPI295790 | | |  |  |  |
| A/equine/Nador/1/1997 | EPI_ISL_119931 | | | EPI383562 | | |  |  |  |
| A/equine/Neuville-Pres-Sees/1/2011 | EPI_ISL_257679 | | | EPI969066 | | |  |  |  |
| A/equine/New Market/1/1979 | EPI_ISL_95199 | | | EPI332930 | | |  |  |  |
| A/equine/New Market/1979 | EPI_ISL_69902 | | | EPI242361 | | |  |  |  |
| A/equine/New Market/nasalwash1/1979 | EPI_ISL_95198 | | | EPI332923 | | |  |  |  |
| A/equine/New York/1/1983 | EPI_ISL_15356 | | | EPI133341 | | |  |  |  |
| A/equine/New York/135857/2016 | EPI_ISL_294936 | | | EPI1157881 | | |  |  |  |
| A/equine/New York/146066/2007 | EPI_ISL_79900 | | | EPI281607 | | |  |  |  |
| A/equine/New York/VR-297/1983 | EPI_ISL_8102 | | | EPI78004 | | |  |  |  |
| A/equine/Newmarket/1/1993 | EPI_ISL_30895 | | | EPI182580 | | |  |  |  |
| A/equine/Newmarket/1/2007 | EPI_ISL_29830 | | | EPI177904 | | |  |  |  |
| A/equine/Newmarket/2/1993 | EPI_ISL_30896 | | | EPI182581 | | |  |  |  |
| A/equine/Newmarket/5/2003 | EPI_ISL_30894 | | | EPI182579 | | |  |  |  |
| A/equine/Northamptonshire/1/2013 | EPI_ISL_151841 | | | EPI584259 | | |  |  |  |
| A/equine/Ohio/1/2003 | EPI_ISL_9534 | | | EPI98546 | | |  |  |  |
| A/equine/Ohio/113461-1/2005 | EPI_ISL_79869 | | | EPI281359 | | |  |  |  |
| A/equine/Ohio/113461-2/2005 | EPI_ISL_79866 | | | EPI281335 | | |  |  |  |
| A/equine/Ohio/113461-2/2005 | EPI_ISL_79866 | | | EPI281335 | | |  |  |  |
| A/equine/Ohio/113461-3/2005 | EPI_ISL_79870 | | | EPI281367 | | |  |  |  |
| A/equine/Oregon/78356/2012 | EPI_ISL_294934 | | | EPI1157847 | | |  |  |  |
| A/equine/Otar/764/2007 | EPI_ISL_89156 | | | EPI314114 | | |  |  |  |
| A/equine/Pennsylvania/1/2007 | EPI_ISL_29839 | | | EPI177916 | | |  |  |  |
| A/equine/Perthshire/3/09 | EPI_ISL_177492 | | | EPI584215 | | |  |  |  |
| A/equine/Pulawy/1/2005 | EPI_ISL_64878 | | | EPI548385 | | |  |  |  |
| A/equine/Pulawy/1/2006 | EPI_ISL_64366 | | | EPI548386 | | |  |  |  |
| A/equine/Pulawy/1/2008 | EPI_ISL_168012 | | | EPI548384 | | |  |  |  |
| A/equine/Qinghai/1/1994 | EPI_ISL_32661 | | | EPI186898 | | |  |  |  |
| A/equine/Richmond/1/2007 | EPI_ISL_29828 | | | EPI177909 | | |  |  |  |
| A/equine/Richmond/2/2007 | EPI_ISL_29829 | | | EPI177922 | | |  |  |  |
| A/equine/Rio Grande do Sul/1/12 | EPI_ISL_177502 | | | EPI584299 | | |  |  |  |
| A/equine/Roma/5/1991 | EPI_ISL_19677 | | | EPI153974 | | |  |  |  |
| A/equine/Romania/1/1980 | EPI_ISL_19680 | | | EPI153998 | | |  |  |  |
| A/equine/Sachiyama/1/1971 | EPI_ISL_25008 | | | EPI165405 | | |  |  |  |
| A/equine/Santa Fe/1/1985 | EPI_ISL_19672 | | | EPI153934 | | |  |  |  |
| A/equine/Sao Paulo/1.19/2012 | EPI_ISL_279065 | | | EPI1065023 | | |  |  |  |
| A/equine/Sao Paulo/1/1969 | EPI_ISL_19681 | | | EPI154006 | | |  |  |  |
| A/equine/Sao Paulo/6/1963 | EPI_ISL_19670 | | | EPI153918 | | |  |  |  |
| A/equine/Saone-et-Loire/1/2015 | EPI_ISL_257683 | | | EPI969069 | | |  |  |  |
| A/equine/Shropshire/10 | EPI_ISL_156748 | | | EPI584231 | | |  |  |  |
| A/equine/Snailwell/98 | EPI_ISL_177489 | | | EPI584191 | | |  |  |  |
| A/equine/Solihull/1/2007 | EPI_ISL_29847 | | | EPI177917 | | |  |  |  |
| A/equine/Solihull/2/2007 | EPI_ISL_29848 | | | EPI177918 | | |  |  |  |
| A/Equine/South Africa/4/03 | EPI_ISL_240665 | | | EPI873604 | | |  |  |  |
| A/equine/South Kazakhstan/236/2012 | EPI_ISL_166607 | | | EPI542622 | | |  |  |  |
| A/equine/Southampton/1/2006 | EPI_ISL_29853 | | | EPI177898 | | |  |  |  |
| A/equine/Southampton/1/2007 | EPI_ISL_29827 | | | EPI177910 | | |  |  |  |
| A/equine/Southampton/2/2007 | EPI_ISL_29849 | | | EPI177920 | | |  |  |  |
| A/equine/Strathaven/1/2007 | EPI_ISL_29850 | | | EPI177921 | | |  |  |  |
| A/equine/Sussex/1/1989 | EPI_ISL_19884 | | | EPI182582 | | |  |  |  |
| A/equine/Sussex/1/1989 | EPI_ISL_19884 | | | EPI182582 | | |  |  |  |
| A/equine/Sussex/93753/89 | EPI_ISL_8280 | | | EPI154464 | | |  |  |  |
| A/Equine/Sweden/SVA111206SZ0085/VIR165837/2011 | EPI_ISL_180684 | | | EPI594042 | | |  |  |  |
| A/equine/Switzerland/1118/1979 | EPI_ISL_19679 | | | EPI153990 | | |  |  |  |
| A/equine/Switzerland/173/1993 | EPI_ISL_19676 | | | EPI153966 | | |  |  |  |
| A/equine/Switzerland/2225/1979 | EPI_ISL_22611 | | | EPI159276 | | |  |  |  |
| A/equine/Switzerland/P112/07 | EPI_ISL_177468 | | | EPI584122 | | |  |  |  |
| A/equine/Switzerland/P112/2007 | EPI_ISL_29841 | | | EPI177902 | | |  |  |  |
| A/equine/Tennessee/27A/2014 | EPI_ISL_220483 | | | EPI753447 | | |  |  |  |
| A/equine/Tennessee/28A/2014 | EPI_ISL_220480 | | | EPI753429 | | |  |  |  |
| A/equine/Tennessee/28B/2014 | EPI_ISL_220484 | | | EPI753472 | | |  |  |  |
| A/equine/Tennessee/29A/2014 | EPI_ISL_220485 | | | EPI753466 | | |  |  |  |
| A/equine/Tennessee/30A/2014 | EPI_ISL_220482 | | | EPI753441 | | |  |  |  |
| A/equine/Tennessee/4A/2014 | EPI_ISL_220481 | | | EPI753436 | | |  |  |  |
| A/equine/Tennessee/5/1986 | EPI_ISL_21061 | | | EPI133246 | | |  |  |  |
| A/equine/Tennessee/5/1986 | EPI_ISL_21061 | | | EPI133246 | | |  |  |  |
| A/equine/Tennessee/5/1986 | EPI_ISL_21061 | | | EPI133246 | | |  |  |  |
| A/equine/Texas/117793/2005 | EPI_ISL_79867 | | | EPI281343 | | |  |  |  |
| A/equine/Texas/117793/2005 | EPI_ISL_79867 | | | EPI281343 | | |  |  |  |
| A/equine/Texas/39655/1991 | EPI_ISL_15349 | | | EPI133189 | | |  |  |  |
| A/equine/Tokyo/2/1971 | EPI_ISL_95197 | | | EPI332911 | | |  |  |  |
| A/equine/Tottori/1/07 | EPI_ISL_89221 | | | EPI314456 | | |  |  |  |
| A/equine/Uruguay/1/1963 | EPI_ISL_21282 | | | EPI154030 | | |  |  |  |
| A/equine/Virginia/131054-3/2005 | EPI_ISL_79899 | | | EPI281599 | | |  |  |  |
| A/equine/Wisconsin/1/03 | EPI_ISL_9783 | | | EPI99385 | | |  |  |  |
| A/equine/Xinjiang/1/2007 | EPI_ISL_32662 | | | EPI186906 | | |  |  |  |
| A/equine/Xinjiang/2/2007 | EPI_ISL_32663 | | | EPI186914 | | |  |  |  |
| A/equine/Xinjiang/3/2007 | EPI_ISL_32664 | | | EPI186922 | | |  |  |  |
| A/equine/Xinjiang/4/2007 | EPI_ISL_32665 | | | EPI186930 | | |  |  |  |
| A/equine/Xinjiang/5/2007 | EPI_ISL_32666 | | | EPI186938 | | |  |  |  |
| A/equine/Xuzhou/01/2013 | EPI_ISL_151130 | | | EPI489634 | | |  |  |  |
| A/equine/Yokohama/aq13/2010 | EPI_ISL_297955 | | | EPI1172958 | | |  |  |  |
| A/equine/Yokohama/aq19/2009 | EPI_ISL_69743 | | | EPI241779 | | |  |  |  |
| A/equine/Yorkshire/3/09 | EPI_ISL_156745 | | | EPI584223 | | |  |  |  |
| A/equine/Yvelines/2136/89 | EPI_ISL_679 | | | EPI3566 | | |  |  |  |
| A/equine 2/Suffolk/89 | EPI_ISL_68956 | | | EPI240109 | | |  |  |  |
| A /Equine/Sweden/SVA111128SZ0073/VIR160172/2011 | EPI_ISL_180755 | | | EPI594744 | | |  |  |  |
| PA | | | | | | | |  |  |
| Isolate_Name | Isolate_Id | | | Segment_Id | | | |  |  |
| A /Equine/Sweden/SVA111128SZ0073/VIR160172/2011 | EPI_ISL_180755 | | | EPI594758 | | | |  |  |
| A/equine/Yorkshire/3/09 | EPI_ISL_156745 | | | EPI584218 | | | |  |  |
| A/equine/Yokohama/aq13/2010 | EPI_ISL_297955 | | | EPI1172953 | | | |  |  |
| A/equine/Xuzhou/01/2013 | EPI_ISL_151130 | | | EPI489640 | | | |  |  |
| A/equine/Xinjiang/5/2007 | EPI_ISL_32666 | | | EPI186933 | | | |  |  |
| A/equine/Xinjiang/4/2007 | EPI_ISL_32665 | | | EPI186925 | | | |  |  |
| A/equine/Xinjiang/3/2007 | EPI_ISL_32664 | | | EPI186917 | | | |  |  |
| A/equine/Xinjiang/2/2007 | EPI_ISL_32663 | | | EPI186909 | | | |  |  |
| A/equine/Xinjiang/1/2007 | EPI_ISL_32662 | | | EPI186901 | | | |  |  |
| A/equine/Wisconsin/1/03 | EPI_ISL_9783 | | | EPI99388 | | | |  |  |
| A/equine/Virginia/131054-3/2005 | EPI_ISL_79899 | | | EPI281594 | | | |  |  |
| A/equine/Uruguay/1/1963 | EPI_ISL_21282 | | | EPI154031 | | | |  |  |
| A/equine/Tottori/1/07 | EPI_ISL_89221 | | | EPI314452 | | | |  |  |
| A/equine/Texas/39655/1991 | EPI_ISL_15349 | | | EPI133192 | | | |  |  |
| A/equine/Texas/117793/2005 | EPI_ISL_79867 | | | EPI281338 | | | |  |  |
| A/equine/Texas/117793/2005 | EPI_ISL_79867 | | | EPI281338 | | | |  |  |
| A/equine/Tennessee/5/1986 | EPI_ISL_21061 | | | EPI133249 | | | |  |  |
| A/equine/Tennessee/5/1986 | EPI_ISL_21061 | | | EPI133249 | | | |  |  |
| A/equine/Tennessee/4A/2014 | EPI_ISL_220481 | | | EPI753432 | | | |  |  |
| A/equine/Tennessee/30A/2014 | EPI_ISL_220482 | | | EPI753438 | | | |  |  |
| A/equine/Tennessee/29A/2014 | EPI_ISL_220485 | | | EPI753456 | | | |  |  |
| A/equine/Tennessee/28B/2014 | EPI_ISL_220484 | | | EPI753450 | | | |  |  |
| A/equine/Tennessee/28A/2014 | EPI_ISL_220480 | | | EPI753460 | | | |  |  |
| A/equine/Tennessee/27A/2014 | EPI_ISL_220483 | | | EPI753444 | | | |  |  |
| A/equine/Switzerland/P112/07 | EPI_ISL_177468 | | | EPI584115 | | | |  |  |
| A/equine/Switzerland/2225/1979 | EPI_ISL_22611 | | | EPI159277 | | | |  |  |
| A/equine/Switzerland/173/1993 | EPI_ISL_19676 | | | EPI153967 | | | |  |  |
| A/equine/Switzerland/1118/1979 | EPI_ISL_19679 | | | EPI153991 | | | |  |  |
| A/Equine/Sweden/SVA111206SZ0085/VIR165837/2011 | EPI_ISL_180684 | | | EPI594054 | | | |  |  |
| A/equine/Sussex/93753/89 | EPI_ISL_8280 | | | EPI154465 | | | |  |  |
| A/equine/Sussex/1/1989 | EPI_ISL_19884 | | | EPI240132 | | | |  |  |
| A/equine/Sussex/1/1989 | EPI_ISL_19884 | | | EPI240132 | | | |  |  |
| A/equine/South Kazakhstan/236/2012 | EPI_ISL_166607 | | | EPI542591 | | | |  |  |
| A/Equine/South Africa/4/03 | EPI_ISL_240665 | | | EPI873603 | | | |  |  |
| A/equine/Snailwell/98 | EPI_ISL_177489 | | | EPI584186 | | | |  |  |
| A/equine/Shropshire/10 | EPI_ISL_156748 | | | EPI584226 | | | |  |  |
| A/equine/Saone-et-Loire/1/2015 | EPI_ISL_257683 | | | EPI969087 | | | |  |  |
| A/equine/Sao Paulo/6/1963 | EPI_ISL_19670 | | | EPI153919 | | | |  |  |
| A/equine/Sao Paulo/1/1969 | EPI_ISL_19681 | | | EPI154007 | | | |  |  |
| A/equine/Sao Paulo/1.19/2012 | EPI_ISL_279065 | | | EPI1065027 | | | |  |  |
| A/equine/Santa Fe/1/1985 | EPI_ISL_19672 | | | EPI153935 | | | |  |  |
| A/equine/Sachiyama/1/1971 | EPI_ISL_25008 | | | EPI165406 | | | |  |  |
| A/equine/Romania/1/1980 | EPI_ISL_19680 | | | EPI153999 | | | |  |  |
| A/equine/Roma/5/1991 | EPI_ISL_19677 | | | EPI153975 | | | |  |  |
| A/equine/Rio Grande do Sul/1/12 | EPI_ISL_177502 | | | EPI584294 | | | |  |  |
| A/equine/Richmond/1/2007 | EPI_ISL_153499 | | | EPI501397 | | | |  |  |
| A/equine/Qinghai/1/1994 | EPI_ISL_32661 | | | EPI186893 | | | |  |  |
| A/equine/Perthshire/3/09 | EPI_ISL_156744 | | | EPI507495 | | | |  |  |
| A/equine/Oregon/78356/2012 | EPI_ISL_294934 | | | EPI1157895 | | | |  |  |
| A/equine/Ohio/113461-3/2005 | EPI_ISL_79870 | | | EPI281362 | | | |  |  |
| A/equine/Ohio/113461-2/2005 | EPI_ISL_79866 | | | EPI281330 | | | |  |  |
| A/equine/Ohio/113461-2/2005 | EPI_ISL_79866 | | | EPI281330 | | | |  |  |
| A/equine/Ohio/113461-1/2005 | EPI_ISL_79869 | | | EPI281354 | | | |  |  |
| A/equine/Ohio/1/2003 | EPI_ISL_9534 | | | EPI98549 | | | |  |  |
| A/equine/Northamptonshire/1/2013 | EPI_ISL_151841 | | | EPI584251 | | | |  |  |
| A/equine/Newmarket/5/2003 | EPI_ISL_30894 | | | EPI235164 | | | |  |  |
| A/equine/Newmarket/2/1993 | EPI_ISL_30896 | | | EPI239742 | | | |  |  |
| A/equine/Newmarket/1/1993 | EPI_ISL_30895 | | | EPI239736 | | | |  |  |
| A/equine/New York/VR-297/1983 | EPI_ISL_8102 | | | EPI78007 | | | |  |  |
| A/equine/New York/146066/2007 | EPI_ISL_79900 | | | EPI281602 | | | |  |  |
| A/equine/New York/135857/2016 | EPI_ISL_294936 | | | EPI1157831 | | | |  |  |
| A/equine/New York/1/1983 | EPI_ISL_15356 | | | EPI133344 | | | |  |  |
| A/equine/New Market/nasalwash1/1979 | EPI_ISL_95198 | | | EPI332922 | | | |  |  |
| A/equine/New Market/1/1979 | EPI_ISL_95199 | | | EPI332929 | | | |  |  |
| A/equine/Neuville-Pres-Sees/1/2011 | EPI_ISL_257679 | | | EPI969084 | | | |  |  |
| A/equine/Mysore/1/2008 | EPI_ISL_74367 | | | EPI580106 | | | |  |  |
| A/equine/Moulton/98 | EPI_ISL_177487 | | | EPI584171 | | | |  |  |
| A/equine/Montana/9564-1/2015 | EPI_ISL_285417 | | | EPI1103406 | | | |  |  |
| A/equine/Montana/9233/2007 | EPI_ISL_79893 | | | EPI281546 | | | |  |  |
| A/equine/Miami/1/1963 | EPI_ISL_8092 | | | EPI77817 | | | |  |  |
| A/equine/Lincolnshire/1/2007 | EPI_ISL_29831 | | | EPI501404 | | | |  |  |
| A/equine/Lincolnshire/06 | EPI_ISL_177471 | | | EPI584126 | | | |  |  |
| A/equine/Liaoning/9/2008 | EPI_ISL_32659 | | | EPI186877 | | | |  |  |
| A/equine/Lanarkshire/09 | EPI_ISL_156747 | | | EPI507498 | | | |  |  |
| A/equine/Kyonggi/SA1/2011 | EPI_ISL_129971 | | | EPI398886 | | | |  |  |
| A/equine/Kentucky/Rosie100/1981 | EPI_ISL_15830 | | | EPI137540 | | | |  |  |
| A/equine/Kentucky/pass_the_pepper1/1976 | EPI_ISL_8093 | | | EPI77836 | | | |  |  |
| A/equine/Kentucky/magnificent_genius1/1981 | EPI_ISL_8100 | | | EPI77969 | | | |  |  |
| A/equine/Kentucky/bitter_boredom5/1976 | EPI_ISL_8094 | | | EPI77855 | | | |  |  |
| A/equine/Kentucky/8/1994 | EPI_ISL_15353 | | | EPI133287 | | | |  |  |
| A/equine/Kentucky/698/1988 | EPI_ISL_15347 | | | EPI133154 | | | |  |  |
| A/equine/Kentucky/694/1988 | EPI_ISL_15346 | | | EPI133135 | | | |  |  |
| A/equine/Kentucky/692/1988 | EPI_ISL_15345 | | | EPI133116 | | | |  |  |
| A/equine/Kentucky/5/02 | EPI_ISL_4663 | | | EPI25740 | | | |  |  |
| A/equine/Kentucky/4/1980 | EPI_ISL_8088 | | | EPI77741 | | | |  |  |
| A/equine/Kentucky/3/1986 | EPI_ISL_19663 | | | EPI153855 | | | |  |  |
| A/equine/Kentucky/3/1981 | EPI_ISL_15342 | | | EPI133040 | | | |  |  |
| A/equine/Kentucky/2/1987 | EPI_ISL_19104 | | | EPI152774 | | | |  |  |
| A/equine/Kentucky/2/1986 | EPI_ISL_21280 | | | EPI153847 | | | |  |  |
| A/equine/Kentucky/2/1981 | EPI_ISL_8090 | | | EPI77779 | | | |  |  |
| A/equine/Kentucky/2/1980 | EPI_ISL_20807 | | | EPI156246 | | | |  |  |
| A/equine/Kentucky/1277/1990 | EPI_ISL_15348 | | | EPI133173 | | | |  |  |
| A/equine/Kentucky/1/1992 | EPI_ISL_15350 | | | EPI133211 | | | |  |  |
| A/equine/Kentucky/1/1991 | EPI_ISL_15352 | | | EPI133268 | | | |  |  |
| A/equine/Kentucky/1/1987 | EPI_ISL_21060 | | | EPI133097 | | | |  |  |
| A/equine/Kentucky/1/1986 | EPI_ISL_15344 | | | EPI133078 | | | |  |  |
| A/equine/Kentucky/1/1981 | EPI_ISL_68957 | | | EPI240115 | | | |  |  |
| A/equine/Kentucky/1/1978 | EPI_ISL_8087 | | | EPI77722 | | | |  |  |
| A/equine/Kentucky/1/11 | EPI_ISL_177500 | | | EPI584277 | | | |  |  |
| A/Equine/Kent/1/15 | EPI_ISL_240663 | | | EPI873588 | | | |  |  |
| A/equine/Katra-Jammu/6/2008 | EPI_ISL_154972 | | | EPI580105 | | | |  |  |
| A/equine/Kascakew/1/1978 | EPI_ISL_69898 | | | EPI242352 | | | |  |  |
| A/equine/Jouars/4/2006 | EPI_ISL_257684 | | | EPI969082 | | | |  |  |
| A/equine/Johannesburg/1/1986 | EPI_ISL_20809 | | | EPI156262 | | | |  |  |
| A/equine/Italy/824/1991 | EPI_ISL_19673 | | | EPI153943 | | | |  |  |
| A/equine/Italy/1199/1992 | EPI_ISL_19674 | | | EPI153951 | | | |  |  |
| A/equine/Italy/1062/1991 | EPI_ISL_19678 | | | EPI153983 | | | |  |  |
| A/equine/Inner Mongolia/8/2008 | EPI_ISL_32660 | | | EPI186885 | | | |  |  |
| A/equine/Idaho/37875/1991 | EPI_ISL_6623 | | | EPI52079 | | | |  |  |
| A/equine/Ibaraki/1/2007 | EPI_ISL_297956 | | | EPI1172961 | | | |  |  |
| A/equine/Hubei/6/2008 | EPI_ISL_32657 | | | EPI186861 | | | |  |  |
| A/equine/Huabei/1/2007 | EPI_ISL_70195 | | | EPI243546 | | | |  |  |
| A/equine/Heilongjiang/SS1/2013 | EPI_ISL_161474 | | | EPI527598 | | | |  |  |
| A/equine/Heilongjiang/10/2008 | EPI_ISL_32658 | | | EPI186869 | | | |  |  |
| A/equine/Heilongjiang/1/2010 | EPI_ISL_144923 | | | EPI464997 | | | |  |  |
| A/Equine/Hampshire/4/2016 | EPI_ISL_240664 | | | EPI873596 | | | |  |  |
| A/equine/Guangxi/1/2008 | EPI_ISL_174545 | | | EPI573751 | | | |  |  |
| A/equine/Gironde/1/2014 | EPI_ISL_257682 | | | EPI969086 | | | |  |  |
| A/equine/Georgia/9/1981 | EPI_ISL_8097 | | | EPI77912 | | | |  |  |
| A/equine/Georgia/3/1981 | EPI_ISL_8096 | | | EPI77893 | | | |  |  |
| A/equine/Georgia/13/1981 | EPI_ISL_8099 | | | EPI77950 | | | |  |  |
| A/equine/Georgia/121362-16/2016 | EPI_ISL_294939 | | | EPI1157857 | | | |  |  |
| A/equine/Georgia/10/1981 | EPI_ISL_8098 | | | EPI77931 | | | |  |  |
| A/equine/Georgia/1/1981 | EPI_ISL_8095 | | | EPI77874 | | | |  |  |
| A/equine/Gansu/7/2008 | EPI_ISL_32656 | | | EPI186853 | | | |  |  |
| A/equine/France/1/1967 | EPI_ISL_22612 | | | EPI159285 | | | |  |  |
| A/equine/Fontainbleu/1/1979 | EPI_ISL_19682 | | | EPI154015 | | | |  |  |
| A/equine/Florida/146609/2011 | EPI_ISL_294944 | | | EPI1157867 | | | |  |  |
| A/Equine/East Sussex/1/2015 | EPI_ISL_240662 | | | EPI873580 | | | |  |  |
| A/equine/East Renfrewshire/2/11 | EPI_ISL_156750 | | | EPI584235 | | | |  |  |
| A/equine/Dubai/1/12 | EPI_ISL_177501 | | | EPI584285 | | | |  |  |
| A/equine/Dorset/09 | EPI_ISL_156746 | | | EPI507497 | | | |  |  |
| A/equine/Devon/1/11 | EPI_ISL_156749 | | | EPI584243 | | | |  |  |
| A/equine/Cordoba/18/1985 | EPI_ISL_19671 | | | EPI153927 | | | |  |  |
| A/equine/Cheshire/06 | EPI_ISL_177472 | | | EPI584134 | | | |  |  |
| A/equine/Cambremer/1/2012 | EPI_ISL_257680 | | | EPI969085 | | | |  |  |
| A/equine/California/8560/2002 | EPI_ISL_15843 | | | EPI137787 | | | |  |  |
| A/equine/California/83/1982 | EPI_ISL_15343 | | | EPI133059 | | | |  |  |
| A/equine/California/4537/1997 | EPI_ISL_15842 | | | EPI137768 | | | |  |  |
| A/equine/California/103/1982 | EPI_ISL_8101 | | | EPI77988 | | | |  |  |
| A/equine/California/1/1980 | EPI_ISL_8089 | | | EPI77760 | | | |  |  |
| A/equine/California/1/10 | EPI_ISL_177498 | | | EPI584268 | | | |  |  |
| A/equine/Buckinghamshire/1/2014 | EPI_ISL_197753 | | | EPI651397 | | | |  |  |
| A/equine/Berlin/1/1989 | EPI_ISL_19683 | | | EPI154023 | | | |  |  |
| A/equine/Belfond/6-2/2009 | EPI_ISL_257677 | | | EPI969083 | | | |  |  |
| A/Equine/Ayrshire/1/2013 | EPI_ISL_240595 | | | EPI873571 | | | |  |  |
| A/equine/Austria/421/1992 | EPI_ISL_19675 | | | EPI153959 | | | |  |  |
| A/equine/Ankara/1/2013 | EPI_ISL_281349 | | | EPI1076905 | | | |  |  |
| A/equine/Almaty/26/2007 | EPI_ISL_71977 | | | EPI383943 | | | |  |  |
| A/equine/Algiers/1/1972 | EPI_ISL_20808 | | | EPI156254 | | | |  |  |
| A/equine/Alaska/29759/1991 | EPI_ISL_15351 | | | EPI133230 | | | |  |  |
| A/equine/Ahmedabad/1/2009 | EPI_ISL_74364 | | | EPI580107 | | | |  |  |
| A/donkey/Shandong/1/2017 | EPI_ISL_284681 | | | EPI1098479 | | | |  |  |
| A/canine/VT/11039/2013 | EPI_ISL_166505 | | | EPI542271 | | | |  |  |
| A/canine/Virginia/93653/2009 | EPI_ISL_82078 | | | EPI289592 | | | |  |  |
| A/canine/Vermont/278213/2013 | EPI_ISL_294941 | | | EPI1157859 | | | |  |  |
| A/canine/Philadelphia/6371100/2008 | EPI_ISL_82057 | | | EPI289137 | | | |  |  |
| A/canine/Pennsylvania/96978/2009 | EPI_ISL_294937 | | | EPI1157836 | | | |  |  |
| A/canine/Pennsylvania/94930-3/2007 | EPI_ISL_79897 | | | EPI281578 | | | |  |  |
| A/canine/Pennsylvania/16699/2007 | EPI_ISL_79890 | | | EPI281522 | | | |  |  |
| A/canine/Pennsylvania/137154/2008 | EPI_ISL_79903 | | | EPI281626 | | | |  |  |
| A/canine/Pennsylvania/10915/2007 | EPI_ISL_79892 | | | EPI281538 | | | |  |  |
| A/canine/Pennsylvania/10909/2007 | EPI_ISL_79891 | | | EPI281530 | | | |  |  |
| A/canine/PA/33225.4/2010 | EPI_ISL_167696 | | | EPI542270 | | | |  |  |
| A/canine/PA/111788.2/2009 | EPI_ISL_166506 | | | EPI542272 | | | |  |  |
| A/canine/NY/dog9c16/2008 | EPI_ISL_81966 | | | EPI289593 | | | |  |  |
| A/canine/NY/dog8c19/2008 | EPI_ISL_82152 | | | EPI289603 | | | |  |  |
| A/canine/NY/dog8c14/2008 | EPI_ISL_81977 | | | EPI289602 | | | |  |  |
| A/canine/NY/dog8c11/2008 | EPI_ISL_82114 | | | EPI289601 | | | |  |  |
| A/canine/NY/dog8c07/2008 | EPI_ISL_81973 | | | EPI289600 | | | |  |  |
| A/canine/NY/dog7c20/2008 | EPI_ISL_81996 | | | EPI289607 | | | |  |  |
| A/canine/NY/dog7c19/2008 | EPI_ISL_81995 | | | EPI289606 | | | |  |  |
| A/canine/NY/dog7c18/2008 | EPI_ISL_82184 | | | EPI289605 | | | |  |  |
| A/canine/NY/dog7c16/2008 | EPI_ISL_81993 | | | EPI289604 | | | |  |  |
| A/canine/NY/dog7c13/2008 | EPI_ISL_81990 | | | EPI289599 | | | |  |  |
| A/canine/NY/dog7c07/2008 | EPI_ISL_81956 | | | EPI289598 | | | |  |  |
| A/canine/NY/dog5c20/2009 | EPI_ISL_82072 | | | EPI289179 | | | |  |  |
| A/canine/NY/dog5c19/2009 | EPI_ISL_82021 | | | EPI289178 | | | |  |  |
| A/canine/NY/dog5c18/2009 | EPI_ISL_82071 | | | EPI289177 | | | |  |  |
| A/canine/NY/dog5c17/2009 | EPI_ISL_82070 | | | EPI289176 | | | |  |  |
| A/canine/NY/dog5c16/2009 | EPI_ISL_82069 | | | EPI289175 | | | |  |  |
| A/canine/NY/dog5c15/2009 | EPI_ISL_82068 | | | EPI289174 | | | |  |  |
| A/canine/NY/dog5c14/2009 | EPI_ISL_82067 | | | EPI289173 | | | |  |  |
| A/canine/NY/dog5c13/2009 | EPI_ISL_82066 | | | EPI289172 | | | |  |  |
| A/canine/NY/dog5c12/2009 | EPI_ISL_82065 | | | EPI289171 | | | |  |  |
| A/canine/NY/dog5c11/2009 | EPI_ISL_82020 | | | EPI289170 | | | |  |  |
| A/canine/NY/dog5c10/2009 | EPI_ISL_82017 | | | EPI289169 | | | |  |  |
| A/canine/NY/dog5c09/2009 | EPI_ISL_82064 | | | EPI289168 | | | |  |  |
| A/canine/NY/dog5c08/2009 | EPI_ISL_82063 | | | EPI289167 | | | |  |  |
| A/canine/NY/dog5c07/2009 | EPI_ISL_82018 | | | EPI289166 | | | |  |  |
| A/canine/NY/dog5c06/2009 | EPI_ISL_82019 | | | EPI289165 | | | |  |  |
| A/canine/NY/dog5c05/2009 | EPI_ISL_82053 | | | EPI289164 | | | |  |  |
| A/canine/NY/dog5c04/2009 | EPI_ISL_82062 | | | EPI289163 | | | |  |  |
| A/canine/NY/dog5c03/2009 | EPI_ISL_82052 | | | EPI289183 | | | |  |  |
| A/canine/NY/dog5c02/2009 | EPI_ISL_82075 | | | EPI289182 | | | |  |  |
| A/canine/NY/dog5c01/2009 | EPI_ISL_82074 | | | EPI289181 | | | |  |  |
| A/canine/NY/dog4c11/2009 | EPI_ISL_82073 | | | EPI289180 | | | |  |  |
| A/canine/NY/dog4c03/2009 | EPI_ISL_82010 | | | EPI289594 | | | |  |  |
| A/canine/NY/dog2c10/2009 | EPI_ISL_82049 | | | EPI289140 | | | |  |  |
| A/canine/NY/dog2c05/2009 | EPI_ISL_82039 | | | EPI289139 | | | |  |  |
| A/canine/NY/dog2c03/2009 | EPI_ISL_82059 | | | EPI289141 | | | |  |  |
| A/canine/NY/dog2c02/2009 | EPI_ISL_82041 | | | EPI289142 | | | |  |  |
| A/canine/NY/dog21c11/2009 | EPI_ISL_82058 | | | EPI289138 | | | |  |  |
| A/canine/NY/dog1c20/2009 | EPI_ISL_82060 | | | EPI289150 | | | |  |  |
| A/canine/NY/dog1c19/2009 | EPI_ISL_82028 | | | EPI289149 | | | |  |  |
| A/canine/NY/dog1c18/2009 | EPI_ISL_82027 | | | EPI289148 | | | |  |  |
| A/canine/NY/dog1c17/2009 | EPI_ISL_82026 | | | EPI289147 | | | |  |  |
| A/canine/NY/dog1c16/2009 | EPI_ISL_82054 | | | EPI289146 | | | |  |  |
| A/canine/NY/dog1c15/2009 | EPI_ISL_82025 | | | EPI289145 | | | |  |  |
| A/canine/NY/dog1c14/2009 | EPI_ISL_82024 | | | EPI289144 | | | |  |  |
| A/canine/NY/dog1c13/2009 | EPI_ISL_82035 | | | EPI289595 | | | |  |  |
| A/canine/NY/dog1c12/2009 | EPI_ISL_82023 | | | EPI289156 | | | |  |  |
| A/canine/NY/dog1c11/2009 | EPI_ISL_82022 | | | EPI289155 | | | |  |  |
| A/canine/NY/dog1c10/2009 | EPI_ISL_82033 | | | EPI289154 | | | |  |  |
| A/canine/NY/dog1c09/2009 | EPI_ISL_82032 | | | EPI289153 | | | |  |  |
| A/canine/NY/dog1c08/2009 | EPI_ISL_82038 | | | EPI289152 | | | |  |  |
| A/canine/NY/dog1c07/2009 | EPI_ISL_82034 | | | EPI289151 | | | |  |  |
| A/canine/NY/dog1c06/2009 | EPI_ISL_82030 | | | EPI289162 | | | |  |  |
| A/canine/NY/dog1c05/2009 | EPI_ISL_82036 | | | EPI289161 | | | |  |  |
| A/canine/NY/dog1c04/2009 | EPI_ISL_82029 | | | EPI289596 | | | |  |  |
| A/canine/NY/dog1c02/2009 | EPI_ISL_82031 | | | EPI289597 | | | |  |  |
| A/canine/NY/dog1c01/2009 | EPI_ISL_82037 | | | EPI289157 | | | |  |  |
| A/canine/NY/dog1c000003/2009 | EPI_ISL_82061 | | | EPI289159 | | | |  |  |
| A/canine/NY/4986/2006 | EPI_ISL_81919 | | | EPI289588 | | | |  |  |
| A/canine/NY/147926/2006 | EPI_ISL_82076 | | | EPI289590 | | | |  |  |
| A/canine/NY/120106.2/2011 | EPI_ISL_166503 | | | EPI542269 | | | |  |  |
| A/canine/NY/105447/2008 | EPI_ISL_174477 | | | EPI573505 | | | |  |  |
| A/canine/NY/100525/2006 | EPI_ISL_81918 | | | EPI289589 | | | |  |  |
| A/canine/New York/51854/2008 | EPI_ISL_79902 | | | EPI281618 | | | |  |  |
| A/canine/New York/5183-6/2006 | EPI_ISL_79871 | | | EPI281370 | | | |  |  |
| A/canine/New York/5183-6/2006 | EPI_ISL_79871 | | | EPI281370 | | | |  |  |
| A/canine/New York/4986-2/2006 | EPI_ISL_79873 | | | EPI281386 | | | |  |  |
| A/canine/New York/3699/2010 | EPI_ISL_294940 | | | EPI1157815 | | | |  |  |
| A/canine/New York/1623.1/2010 | EPI_ISL_294943 | | | EPI1157840 | | | |  |  |
| A/canine/New York/159903/2012 | EPI_ISL_294938 | | | EPI1157882 | | | |  |  |
| A/canine/New York/158402-1/2008 | EPI_ISL_79905 | | | EPI281642 | | | |  |  |
| A/canine/New York/147926-5/2006 | EPI_ISL_79888 | | | EPI281506 | | | |  |  |
| A/canine/New York/147926-3/2006 | EPI_ISL_79887 | | | EPI281498 | | | |  |  |
| A/canine/New York/145353/2008 | EPI_ISL_79904 | | | EPI281634 | | | |  |  |
| A/canine/New York/115809/2005 | EPI_ISL_79864 | | | EPI281314 | | | |  |  |
| A/canine/New York/115719/2007 | EPI_ISL_79896 | | | EPI281570 | | | |  |  |
| A/canine/New York/100528-6/2006 | EPI_ISL_79886 | | | EPI281490 | | | |  |  |
| A/canine/New York/100528-5/2006 | EPI_ISL_79885 | | | EPI281482 | | | |  |  |
| A/canine/New York/100528-1/2006 | EPI_ISL_79884 | | | EPI281474 | | | |  |  |
| A/canine/New York/100525-1/2006 | EPI_ISL_79883 | | | EPI281466 | | | |  |  |
| A/canine/Miami/2005 | EPI_ISL_64819 | | | EPI226915 | | | |  |  |
| A/canine/Massachusetts/26810/2016 | EPI_ISL_234612 | | | EPI838686 | | | |  |  |
| A/canine/Maine/058124/2016 | EPI_ISL_294942 | | | EPI1157822 | | | |  |  |
| A/canine/Kentucky/118778/2006 | EPI_ISL_79889 | | | EPI281514 | | | |  |  |
| A/canine/Jacksonville/2005 | EPI_ISL_64818 | | | EPI226909 | | | |  |  |
| A/canine/Florida/89911-2/2006 | EPI_ISL_79882 | | | EPI281458 | | | |  |  |
| A/canine/Florida/78592-7/2006 | EPI_ISL_79881 | | | EPI281450 | | | |  |  |
| A/canine/Florida/78592-6/2006 | EPI_ISL_79880 | | | EPI281442 | | | |  |  |
| A/canine/Florida/78592-2/2006 | EPI_ISL_79879 | | | EPI281434 | | | |  |  |
| A/canine/Florida/61156-2/2006 | EPI_ISL_79895 | | | EPI281562 | | | |  |  |
| A/canine/Florida/61156-2/2006 | EPI_ISL_79895 | | | EPI281562 | | | |  |  |
| A/canine/Florida/43/2004 | EPI_ISL_9527 | | | EPI98465 | | | |  |  |
| A/canine/Florida/242/2003 | EPI_ISL_9528 | | | EPI98481 | | | |  |  |
| A/canine/Florida/15592.1/2004 | EPI_ISL_294945 | | | EPI1157861 | | | |  |  |
| A/canine/Connecticut/85863/2011 | EPI_ISL_294935 | | | EPI1157818 | | | |  |  |
| A/canine/Colorado/8880/2006 | EPI_ISL_82095 | | | EPI289587 | | | |  |  |
| A/canine/Colorado/8880/2006 | EPI_ISL_82095 | | | EPI289587 | | | |  |  |
| A/canine/Colorado/6723-8/2008 | EPI_ISL_79901 | | | EPI281610 | | | |  |  |
| A/canine/Colorado/6723-8/2008 | EPI_ISL_79901 | | | EPI281610 | | | |  |  |
| A/canine/Colorado/6723-14/2008 | EPI_ISL_82077 | | | EPI289591 | | | |  |  |
| A/canine/Colorado/30604/2006 | EPI_ISL_79877 | | | EPI281418 | | | |  |  |
| A/canine/Colorado/17864/2006 | EPI_ISL_79875 | | | EPI281402 | | | |  |  |
| A/canine/Colorado/17864/2006 | EPI_ISL_79875 | | | EPI281402 | | | |  |  |
| A/canine/California/70645-4/2006 | EPI_ISL_79878 | | | EPI281426 | | | |  |  |
| PB1 | | | | | | | |  |  |
| Isolate_Name | | Isolate_Id | | Segment_Id | | | |  |  |
| A/canine/California/70645-4/2006 | | EPI_ISL_79878 | | EPI281425 | | | |  |  |
| A/canine/Colorado/17864/2006 | | EPI_ISL_79875 | | EPI281401 | | | |  |  |
| A/canine/Colorado/17864/2006 | | EPI_ISL_79875 | | EPI281401 | | | |  |  |
| A/canine/Colorado/30604/2006 | | EPI_ISL_79877 | | EPI281417 | | | |  |  |
| A/canine/Colorado/6723-14/2008 | | EPI_ISL_82077 | | EPI289187 | | | |  |  |
| A/canine/Colorado/6723-8/2008 | | EPI_ISL_79901 | | EPI281609 | | | |  |  |
| A/canine/Colorado/6723-8/2008 | | EPI_ISL_79901 | | EPI281609 | | | |  |  |
| A/canine/Colorado/8880/2006 | | EPI_ISL_79874 | | EPI281393 | | | |  |  |
| A/canine/Connecticut/85863/2011 | | EPI_ISL_294935 | | EPI1157888 | | | |  |  |
| A/canine/Florida/15592.1/2004 | | EPI_ISL_294945 | | EPI1157837 | | | |  |  |
| A/canine/Florida/242/2003 | | EPI_ISL_9528 | | EPI98479 | | | |  |  |
| A/canine/Florida/43/2004 | | EPI_ISL_9527 | | EPI98463 | | | |  |  |
| A/canine/Florida/61156-2/2006 | | EPI_ISL_79895 | | EPI281561 | | | |  |  |
| A/canine/Florida/61156-2/2006 | | EPI_ISL_79895 | | EPI281561 | | | |  |  |
| A/canine/Florida/78592-2/2006 | | EPI_ISL_79879 | | EPI281433 | | | |  |  |
| A/canine/Florida/78592-6/2006 | | EPI_ISL_79880 | | EPI281441 | | | |  |  |
| A/canine/Florida/78592-7/2006 | | EPI_ISL_79881 | | EPI281449 | | | |  |  |
| A/canine/Florida/89911-2/2006 | | EPI_ISL_79882 | | EPI281457 | | | |  |  |
| A/canine/Jacksonville/2005 | | EPI_ISL_64818 | | EPI226908 | | | |  |  |
| A/canine/Kentucky/118778/2006 | | EPI_ISL_79889 | | EPI281513 | | | |  |  |
| A/canine/Maine/058124/2016 | | EPI_ISL_294942 | | EPI1157897 | | | |  |  |
| A/canine/Massachusetts/26810/2016 | | EPI_ISL_234612 | | EPI839110 | | | |  |  |
| A/canine/Miami/2005 | | EPI_ISL_64819 | | EPI226914 | | | |  |  |
| A/canine/New York/100525-1/2006 | | EPI_ISL_79883 | | EPI281465 | | | |  |  |
| A/canine/New York/100528-1/2006 | | EPI_ISL_79884 | | EPI281473 | | | |  |  |
| A/canine/New York/100528-5/2006 | | EPI_ISL_79885 | | EPI281481 | | | |  |  |
| A/canine/New York/100528-6/2006 | | EPI_ISL_79886 | | EPI281489 | | | |  |  |
| A/canine/New York/115719/2007 | | EPI_ISL_79896 | | EPI281569 | | | |  |  |
| A/canine/New York/115809/2005 | | EPI_ISL_79864 | | EPI281313 | | | |  |  |
| A/canine/New York/145353/2008 | | EPI_ISL_79904 | | EPI281633 | | | |  |  |
| A/canine/New York/147926-3/2006 | | EPI_ISL_79887 | | EPI281497 | | | |  |  |
| A/canine/New York/147926-5/2006 | | EPI_ISL_79888 | | EPI281505 | | | |  |  |
| A/canine/New York/158402-1/2008 | | EPI_ISL_79905 | | EPI281641 | | | |  |  |
| A/canine/New York/159903/2012 | | EPI_ISL_294938 | | EPI1157809 | | | |  |  |
| A/canine/New York/1623.1/2010 | | EPI_ISL_294943 | | EPI1157834 | | | |  |  |
| A/canine/New York/4986-2/2006 | | EPI_ISL_79873 | | EPI281385 | | | |  |  |
| A/canine/New York/5183-6/2006 | | EPI_ISL_79871 | | EPI281369 | | | |  |  |
| A/canine/New York/5183-6/2006 | | EPI_ISL_79871 | | EPI281369 | | | |  |  |
| A/canine/New York/51854/2008 | | EPI_ISL_79902 | | EPI281617 | | | |  |  |
| A/canine/NY/100525/2006 | | EPI_ISL_81918 | | EPI289612 | | | |  |  |
| A/canine/NY/105447/2008 | | EPI_ISL_174477 | | EPI576527 | | | |  |  |
| A/canine/NY/120106.2/2011 | | EPI_ISL_166503 | | EPI546865 | | | |  |  |
| A/canine/NY/147926/2006 | | EPI_ISL_82076 | | EPI289613 | | | |  |  |
| A/canine/NY/4986/2006 | | EPI_ISL_81919 | | EPI289611 | | | |  |  |
| A/canine/NY/dog21c05/2009 | | EPI_ISL_82081 | | EPI289191 | | | |  |  |
| A/canine/NY/dog21c10/2009 | | EPI_ISL_82080 | | EPI289190 | | | |  |  |
| A/canine/NY/dog21c13/2009 | | EPI_ISL_82079 | | EPI289189 | | | |  |  |
| A/canine/NY/dog4c01/2009 | | EPI_ISL_82013 | | EPI289201 | | | |  |  |
| A/canine/NY/dog4c07/2009 | | EPI_ISL_82085 | | EPI289202 | | | |  |  |
| A/canine/NY/dog4c11/2009 | | EPI_ISL_82073 | | EPI289203 | | | |  |  |
| A/canine/NY/dog4c12/2009 | | EPI_ISL_82000 | | EPI289204 | | | |  |  |
| A/canine/NY/dog4c16/2009 | | EPI_ISL_82002 | | EPI289205 | | | |  |  |
| A/canine/NY/dog4c18/2009 | | EPI_ISL_82086 | | EPI289206 | | | |  |  |
| A/canine/NY/dog4c19/2009 | | EPI_ISL_82087 | | EPI289608 | | | |  |  |
| A/canine/NY/dog4c23/2009 | | EPI_ISL_82088 | | EPI289208 | | | |  |  |
| A/canine/NY/dog4c32/2009 | | EPI_ISL_82089 | | EPI289209 | | | |  |  |
| A/canine/NY/dog5c03/2009 | | EPI_ISL_82052 | | EPI289210 | | | |  |  |
| A/canine/NY/dog5c04/2009 | | EPI_ISL_82062 | | EPI289211 | | | |  |  |
| A/canine/NY/dog5c05/2009 | | EPI_ISL_82053 | | EPI289214 | | | |  |  |
| A/canine/NY/dog5c06/2009 | | EPI_ISL_82019 | | EPI289213 | | | |  |  |
| A/canine/NY/dog5c07/2009 | | EPI_ISL_82018 | | EPI289609 | | | |  |  |
| A/canine/NY/dog5c09/2009 | | EPI_ISL_82064 | | EPI289212 | | | |  |  |
| A/canine/NY/dog5c12/2009 | | EPI_ISL_82065 | | EPI289196 | | | |  |  |
| A/canine/NY/dog5c15/2009 | | EPI_ISL_82068 | | EPI289610 | | | |  |  |
| A/canine/NY/dog6c05/2009 | | EPI_ISL_81999 | | EPI289215 | | | |  |  |
| A/canine/NY/dog6c06/2009 | | EPI_ISL_82090 | | EPI289216 | | | |  |  |
| A/canine/NY/dog6c07/2009 | | EPI_ISL_82001 | | EPI289217 | | | |  |  |
| A/canine/NY/dog6c09/2009 | | EPI_ISL_82091 | | EPI289218 | | | |  |  |
| A/canine/NY/dog6c11/2009 | | EPI_ISL_82092 | | EPI289219 | | | |  |  |
| A/canine/NY/dog6c13/2009 | | EPI_ISL_82082 | | EPI289198 | | | |  |  |
| A/canine/NY/dog6c14/2009 | | EPI_ISL_82083 | | EPI289199 | | | |  |  |
| A/canine/NY/dog6c16/2009 | | EPI_ISL_82084 | | EPI289200 | | | |  |  |
| A/canine/NY/dog8c03/2008 | | EPI_ISL_81971 | | EPI289195 | | | |  |  |
| A/canine/NY/dog8c05/2008 | | EPI_ISL_81981 | | EPI289193 | | | |  |  |
| A/canine/NY/dog8c06/2008 | | EPI_ISL_81972 | | EPI289194 | | | |  |  |
| A/canine/PA/111788.2/2009 | | EPI_ISL_166506 | | EPI546868 | | | |  |  |
| A/canine/PA/33225.4/2010 | | EPI_ISL_167696 | | EPI546866 | | | |  |  |
| A/canine/Pennsylvania/10909/2007 | | EPI_ISL_79891 | | EPI281529 | | | |  |  |
| A/canine/Pennsylvania/10915/2007 | | EPI_ISL_79892 | | EPI281537 | | | |  |  |
| A/canine/Pennsylvania/137154/2008 | | EPI_ISL_79903 | | EPI281625 | | | |  |  |
| A/canine/Pennsylvania/16699/2007 | | EPI_ISL_79890 | | EPI281521 | | | |  |  |
| A/canine/Pennsylvania/94930-3/2007 | | EPI_ISL_79897 | | EPI281577 | | | |  |  |
| A/canine/Pennsylvania/96978/2009 | | EPI_ISL_294937 | | EPI1157826 | | | |  |  |
| A/canine/Philadelphia/6371100/2008 | | EPI_ISL_82057 | | EPI289192 | | | |  |  |
| A/canine/StatenIs/115719/2007 | | EPI_ISL_81921 | | EPI289614 | | | |  |  |
| A/canine/Vermont/278213/2013 | | EPI_ISL_294941 | | EPI1157854 | | | |  |  |
| A/canine/Virginia/93653/2009 | | EPI_ISL_82078 | | EPI289615 | | | |  |  |
| A/canine/VT/11039/2013 | | EPI_ISL_166505 | | EPI546867 | | | |  |  |
| A/donkey/Shandong/1/2017 | | EPI_ISL_284681 | | EPI1098478 | | | |  |  |
| A/equine/Ahmedabad/1/2009 | | EPI_ISL_74364 | | EPI580104 | | | |  |  |
| A/equine/Alaska/29759/1991 | | EPI_ISL_15351 | | EPI133232 | | | |  |  |
| A/equine/Algiers/1/1972 | | EPI_ISL_20808 | | EPI156255 | | | |  |  |
| A/equine/Ankara/1/2013 | | EPI_ISL_281349 | | EPI1076904 | | | |  |  |
| A/equine/Athens/02/2003 | | EPI_ISL_75320 | | EPI347188 | | | |  |  |
| A/equine/Athens/04/2007 | | EPI_ISL_28012 | | EPI347190 | | | |  |  |
| A/equine/Austria/421/1992 | | EPI_ISL_19675 | | EPI153960 | | | |  |  |
| A/Equine/Ayrshire/1/2013 | | EPI_ISL_240595 | | EPI873300 | | | |  |  |
| A/equine/Belfond/6-2/2009 | | EPI_ISL_257677 | | EPI969077 | | | |  |  |
| A/equine/Berlin/1/1989 | | EPI_ISL_19683 | | EPI154024 | | | |  |  |
| A/equine/Buckinghamshire/1/2014 | | EPI_ISL_197753 | | EPI662160 | | | |  |  |
| A/equine/California/1/10 | | EPI_ISL_177498 | | EPI584267 | | | |  |  |
| A/equine/California/1/1980 | | EPI_ISL_8089 | | EPI77762 | | | |  |  |
| A/equine/California/103/1982 | | EPI_ISL_8101 | | EPI77990 | | | |  |  |
| A/equine/California/191/2003 | | EPI_ISL_9533 | | EPI98525 | | | |  |  |
| A/equine/California/4537/1997 | | EPI_ISL_15842 | | EPI137770 | | | |  |  |
| A/equine/California/83/1982 | | EPI_ISL_15343 | | EPI133061 | | | |  |  |
| A/equine/California/8560/2002 | | EPI_ISL_15843 | | EPI137789 | | | |  |  |
| A/equine/Cambremer/1/2012 | | EPI_ISL_257680 | | EPI969079 | | | |  |  |
| A/equine/Cheshire/06 | | EPI_ISL_177472 | | EPI584133 | | | |  |  |
| A/equine/Cordoba/18/1985 | | EPI_ISL_19671 | | EPI153928 | | | |  |  |
| A/equine/Devon/1/11 | | EPI_ISL_156749 | | EPI584242 | | | |  |  |
| A/equine/Dorset/09 | | EPI_ISL_177491 | | EPI584201 | | | |  |  |
| A/equine/Dubai/1/12 | | EPI_ISL_177501 | | EPI584284 | | | |  |  |
| A/equine/East Renfrewshire/2/11 | | EPI_ISL_156750 | | EPI584234 | | | |  |  |
| A/Equine/East Sussex/1/2015 | | EPI_ISL_240662 | | EPI873608 | | | |  |  |
| A/equine/Florida/146609/2011 | | EPI_ISL_294944 | | EPI1157842 | | | |  |  |
| A/equine/Fontainbleu/1/1979 | | EPI_ISL_19682 | | EPI154016 | | | |  |  |
| A/equine/France/1/1967 | | EPI_ISL_22612 | | EPI159286 | | | |  |  |
| A/equine/Gansu/7/2008 | | EPI_ISL_32656 | | EPI186852 | | | |  |  |
| A/equine/Georgia/1/1981 | | EPI_ISL_8095 | | EPI77876 | | | |  |  |
| A/equine/Georgia/10/1981 | | EPI_ISL_8098 | | EPI77933 | | | |  |  |
| A/equine/Georgia/121362-16/2016 | | EPI_ISL_294939 | | EPI1157891 | | | |  |  |
| A/equine/Georgia/13/1981 | | EPI_ISL_8099 | | EPI77952 | | | |  |  |
| A/equine/Georgia/3/1981 | | EPI_ISL_8096 | | EPI77895 | | | |  |  |
| A/equine/Georgia/9/1981 | | EPI_ISL_8097 | | EPI77914 | | | |  |  |
| A/equine/Gironde/1/2014 | | EPI_ISL_257682 | | EPI969080 | | | |  |  |
| A/equine/Guangxi/1/2008 | | EPI_ISL_174545 | | EPI573747 | | | |  |  |
| A/Equine/Hampshire/4/2016 | | EPI_ISL_240664 | | EPI873595 | | | |  |  |
| A/equine/Heilongjiang/1/2010 | | EPI_ISL_103105 | | EPI515348 | | | |  |  |
| A/equine/Heilongjiang/10/2008 | | EPI_ISL_32658 | | EPI186868 | | | |  |  |
| A/equine/Heilongjiang/SS1/2013 | | EPI_ISL_161474 | | EPI527600 | | | |  |  |
| A/equine/Himachal Pradesh/CMVL-YOL2/2008 | | EPI_ISL_190904 | | EPI621742 | | | |  |  |
| A/equine/Hissar/CMVL-HSR4/2008 | | EPI_ISL_190901 | | EPI621723 | | | |  |  |
| A/equine/Huabei/1/2007 | | EPI_ISL_70195 | | EPI243545 | | | |  |  |
| A/equine/Hubei/6/2008 | | EPI_ISL_32657 | | EPI186860 | | | |  |  |
| A/equine/Ibaraki/1/2007 | | EPI_ISL_297956 | | EPI1172960 | | | |  |  |
| A/equine/Idaho/37875/1991 | | EPI_ISL_6623 | | EPI52081 | | | |  |  |
| A/equine/Inner Mongolia/8/2008 | | EPI_ISL_32660 | | EPI186884 | | | |  |  |
| A/equine/Italy/1062/1991 | | EPI_ISL_19678 | | EPI153984 | | | |  |  |
| A/equine/Italy/1199/1992 | | EPI_ISL_19674 | | EPI153952 | | | |  |  |
| A/equine/Italy/824/1991 | | EPI_ISL_19673 | | EPI153944 | | | |  |  |
| A/equine/Jammu and Kashmir/CMVL-LEH4/2008 | | EPI_ISL_190902 | | EPI621729 | | | |  |  |
| A/equine/Jammu and Kashmir/CMVL-LEH6/2008 | | EPI_ISL_190903 | | EPI621735 | | | |  |  |
| A/equine/Johannesburg/1/1986 | | EPI_ISL_20809 | | EPI156263 | | | |  |  |
| A/equine/Jouars/4/2006 | | EPI_ISL_257684 | | EPI969076 | | | |  |  |
| A/equine/Kascakew/1/1978 | | EPI_ISL_69898 | | EPI242351 | | | |  |  |
| A/equine/Katra-Jammu/6/2008 | | EPI_ISL_154972 | | EPI580102 | | | |  |  |
| A/Equine/Kent/1/15 | | EPI_ISL_240663 | | EPI873587 | | | |  |  |
| A/equine/Kentucky/1/11 | | EPI_ISL_177500 | | EPI584276 | | | |  |  |
| A/equine/Kentucky/1/1978 | | EPI_ISL_8087 | | EPI77724 | | | |  |  |
| A/equine/Kentucky/1/1981 | | EPI_ISL_68957 | | EPI240116 | | | |  |  |
| A/equine/Kentucky/1/1986 | | EPI_ISL_15344 | | EPI133080 | | | |  |  |
| A/equine/Kentucky/1/1987 | | EPI_ISL_21060 | | EPI133099 | | | |  |  |
| A/equine/Kentucky/1/1991 | | EPI_ISL_15352 | | EPI133270 | | | |  |  |
| A/equine/Kentucky/1/1992 | | EPI_ISL_15350 | | EPI133213 | | | |  |  |
| A/equine/Kentucky/1277/1990 | | EPI_ISL_15348 | | EPI133175 | | | |  |  |
| A/equine/Kentucky/2/1980 | | EPI_ISL_20807 | | EPI156247 | | | |  |  |
| A/equine/Kentucky/2/1981 | | EPI_ISL_8090 | | EPI77781 | | | |  |  |
| A/equine/Kentucky/2/1986 | | EPI_ISL_21280 | | EPI153848 | | | |  |  |
| A/equine/Kentucky/2/1987 | | EPI_ISL_19104 | | EPI152775 | | | |  |  |
| A/equine/Kentucky/3/1981 | | EPI_ISL_15342 | | EPI133042 | | | |  |  |
| A/equine/Kentucky/3/1986 | | EPI_ISL_19663 | | EPI153856 | | | |  |  |
| A/equine/Kentucky/4/1980 | | EPI_ISL_8088 | | EPI77743 | | | |  |  |
| A/equine/Kentucky/5/02 | | EPI_ISL_4663 | | EPI25738 | | | |  |  |
| A/equine/Kentucky/5/2002 | | EPI_ISL_9530 | | EPI98523 | | | |  |  |
| A/equine/Kentucky/692/1988 | | EPI_ISL_15345 | | EPI133118 | | | |  |  |
| A/equine/Kentucky/694/1988 | | EPI_ISL_15346 | | EPI133137 | | | |  |  |
| A/equine/Kentucky/698/1988 | | EPI_ISL_15347 | | EPI133156 | | | |  |  |
| A/equine/Kentucky/8/1994 | | EPI_ISL_15353 | | EPI133289 | | | |  |  |
| A/equine/Kentucky/bitter_boredom5/1976 | | EPI_ISL_8094 | | EPI77857 | | | |  |  |
| A/equine/Kentucky/magnificent_genius1/1981 | | EPI_ISL_8100 | | EPI77971 | | | |  |  |
| A/equine/Kentucky/pass_the_pepper1/1976 | | EPI_ISL_8093 | | EPI77838 | | | |  |  |
| A/equine/Kentucky/Rosie100/1981 | | EPI_ISL_15830 | | EPI137542 | | | |  |  |
| A/equine/Kyonggi/SA1/2011 | | EPI_ISL_129971 | | EPI398885 | | | |  |  |
| A/equine/Lanarkshire/09 | | EPI_ISL_177490 | | EPI584193 | | | |  |  |
| A/equine/Liaoning/9/2008 | | EPI_ISL_32659 | | EPI186876 | | | |  |  |
| A/equine/Lincolnshire/06 | | EPI_ISL_177471 | | EPI584125 | | | |  |  |
| A/equine/Lincolnshire/1/2007 | | EPI_ISL_29831 | | EPI501403 | | | |  |  |
| A/equine/Massachussetts/213/2003 | | EPI_ISL_9529 | | EPI98515 | | | |  |  |
| A/equine/Miami/1/1963 | | EPI_ISL_8092 | | EPI126494 | | | |  |  |
| A/equine/Montana/9233/2007 | | EPI_ISL_79893 | | EPI281545 | | | |  |  |
| A/equine/Montana/9564-1/2015 | | EPI_ISL_285417 | | EPI1103405 | | | |  |  |
| A/equine/Moulton/98 | | EPI_ISL_177487 | | EPI584170 | | | |  |  |
| A/equine/Mysore/1/2008 | | EPI_ISL_74367 | | EPI580103 | | | |  |  |
| A/equine/Neuville-Pres-Sees/1/2011 | | EPI_ISL_257679 | | EPI969078 | | | |  |  |
| A/equine/New Market/1/1979 | | EPI_ISL_95199 | | EPI332928 | | | |  |  |
| A/equine/New York/1/1983 | | EPI_ISL_15356 | | EPI133346 | | | |  |  |
| A/equine/New York/1/1999 | | EPI_ISL_9531 | | EPI98521 | | | |  |  |
| A/equine/New York/135857/2016 | | EPI_ISL_294936 | | EPI1157887 | | | |  |  |
| A/equine/New York/146066/2007 | | EPI_ISL_79900 | | EPI281601 | | | |  |  |
| A/equine/New York/452/2003 | | EPI_ISL_9532 | | EPI98519 | | | |  |  |
| A/equine/New York/VR-297/1983 | | EPI_ISL_8102 | | EPI78009 | | | |  |  |
| A/equine/Newmarket/1/1993 | | EPI_ISL_30895 | | EPI239737 | | | |  |  |
| A/equine/Newmarket/2/1993 | | EPI_ISL_30896 | | EPI239743 | | | |  |  |
| A/equine/Newmarket/5/2003 | | EPI_ISL_30894 | | EPI235165 | | | |  |  |
| A/equine/Northamptonshire/1/2013 | | EPI_ISL_151841 | | EPI584250 | | | |  |  |
| A/equine/Ohio/1/2003 | | EPI_ISL_9534 | | EPI98517 | | | |  |  |
| A/equine/Ohio/113461-1/2005 | | EPI_ISL_79869 | | EPI281353 | | | |  |  |
| A/equine/Ohio/113461-2/2005 | | EPI_ISL_79866 | | EPI281329 | | | |  |  |
| A/equine/Ohio/113461-2/2005 | | EPI_ISL_79866 | | EPI281329 | | | |  |  |
| A/equine/Ohio/113461-3/2005 | | EPI_ISL_79870 | | EPI281361 | | | |  |  |
| A/equine/Oregon/78356/2012 | | EPI_ISL_294934 | | EPI1157855 | | | |  |  |
| A/equine/Perthshire/3/09 | | EPI_ISL_177492 | | EPI584209 | | | |  |  |
| A/equine/Qinghai/1/1994 | | EPI_ISL_32661 | | EPI186892 | | | |  |  |
| A/equine/Richmond/1/2007 | | EPI_ISL_153499 | | EPI501402 | | | |  |  |
| A/equine/Rio Grande do Sul/1/12 | | EPI_ISL_177502 | | EPI584293 | | | |  |  |
| A/equine/Roma/5/1991 | | EPI_ISL_19677 | | EPI153976 | | | |  |  |
| A/equine/Romania/1/1980 | | EPI_ISL_19680 | | EPI154000 | | | |  |  |
| A/equine/Sachiyama/1/1971 | | EPI_ISL_25008 | | EPI165407 | | | |  |  |
| A/equine/Santa Fe/1/1985 | | EPI_ISL_19672 | | EPI153936 | | | |  |  |
| A/equine/Sao Paulo/1.19/2012 | | EPI_ISL_279065 | | EPI1065028 | | | |  |  |
| A/equine/Sao Paulo/1/1969 | | EPI_ISL_19681 | | EPI154008 | | | |  |  |
| A/equine/Sao Paulo/6/1963 | | EPI_ISL_19670 | | EPI153920 | | | |  |  |
| A/equine/Saone-et-Loire/1/2015 | | EPI_ISL_257683 | | EPI969081 | | | |  |  |
| A/equine/Shropshire/10 | | EPI_ISL_156748 | | EPI584225 | | | |  |  |
| A/equine/Snailwell/98 | | EPI_ISL_177489 | | EPI584185 | | | |  |  |
| A/equine/South Kazakhstan/236/2012 | | EPI_ISL_166607 | | EPI542623 | | | |  |  |
| A/equine/Sussex/1/1989 | | EPI_ISL_19884 | | EPI240133 | | | |  |  |
| A/equine/Sussex/1/1989 | | EPI_ISL_19884 | | EPI240133 | | | |  |  |
| A/equine/Sussex/93753/89 | | EPI_ISL_8280 | | EPI154466 | | | |  |  |
| A/Equine/Sweden/SVA111206SZ0085/VIR165837/2011 | | EPI_ISL_180684 | | EPI594111 | | | |  |  |
| A/equine/Switzerland/1118/1979 | | EPI_ISL_19679 | | EPI153992 | | | |  |  |
| A/equine/Switzerland/173/1993 | | EPI_ISL_19676 | | EPI153968 | | | |  |  |
| A/equine/Switzerland/2225/1979 | | EPI_ISL_22611 | | EPI159278 | | | |  |  |
| A/equine/Switzerland/P112/07 | | EPI_ISL_177468 | | EPI584112 | | | |  |  |
| A/equine/Tennessee/27A/2014 | | EPI_ISL_220483 | | EPI753443 | | | |  |  |
| A/equine/Tennessee/28A/2014 | | EPI_ISL_220480 | | EPI753468 | | | |  |  |
| A/equine/Tennessee/28B/2014 | | EPI_ISL_220484 | | EPI753449 | | | |  |  |
| A/equine/Tennessee/29A/2014 | | EPI_ISL_220485 | | EPI753455 | | | |  |  |
| A/equine/Tennessee/30A/2014 | | EPI_ISL_220482 | | EPI753437 | | | |  |  |
| A/equine/Tennessee/4A/2014 | | EPI_ISL_220481 | | EPI753431 | | | |  |  |
| A/equine/Tennessee/5/1986 | | EPI_ISL_21061 | | EPI133251 | | | |  |  |
| A/equine/Tennessee/5/1986 | | EPI_ISL_21061 | | EPI133251 | | | |  |  |
| A/equine/Texas/117793/2005 | | EPI_ISL_79867 | | EPI281337 | | | |  |  |
| A/equine/Texas/117793/2005 | | EPI_ISL_79867 | | EPI281337 | | | |  |  |
| A/equine/Texas/39655/1991 | | EPI_ISL_15349 | | EPI133194 | | | |  |  |
| A/equine/Tottori/1/07 | | EPI_ISL_89221 | | EPI314453 | | | |  |  |
| A/equine/Uruguay/1/1963 | | EPI_ISL_21282 | | EPI154032 | | | |  |  |
| A/equine/Virginia/131054-3/2005 | | EPI_ISL_79899 | | EPI281593 | | | |  |  |
| A/equine/Wisconsin/1/03 | | EPI_ISL_9783 | | EPI99390 | | | |  |  |
| A/equine/Xinjiang/1/2007 | | EPI_ISL_32662 | | EPI186900 | | | |  |  |
| A/equine/Xinjiang/2/2007 | | EPI_ISL_32663 | | EPI186908 | | | |  |  |
| A/equine/Xinjiang/3/2007 | | EPI_ISL_32664 | | EPI186916 | | | |  |  |
| A/equine/Xinjiang/4/2007 | | EPI_ISL_32665 | | EPI186924 | | | |  |  |
| A/equine/Xinjiang/5/2007 | | EPI_ISL_32666 | | EPI186932 | | | |  |  |
| A/equine/Xuzhou/01/2013 | | EPI_ISL_151130 | | EPI489636 | | | |  |  |
| A/equine/Yokohama/aq13/2010 | | EPI_ISL_297955 | | EPI1172952 | | | |  |  |
| A/equine/Yorkshire/3/09 | | EPI_ISL_156745 | | EPI584217 | | | |  |  |
| A /Equine/Sweden/SVA111128SZ0073/VIR160172/2011 | | EPI_ISL_180755 | | EPI594796 | | | |  |  |
| PB2 | | | | | | | | |  |
| Isolate_Name | | | Isolate_Id | | Segment_Id | | | |  |
| A /Equine/Sweden/SVA111128SZ0073/VIR160172/2011 | | | EPI_ISL_180755 | | EPI594819 | | | |  |
| A/equine/Yorkshire/3/09 | | | EPI_ISL_156745 | | EPI584216 | | | |  |
| A/equine/Yokohama/aq13/2010 | | | EPI_ISL_297955 | | EPI1172951 | | | |  |
| A/equine/Xuzhou/01/2013 | | | EPI_ISL_151130 | | EPI489637 | | | |  |
| A/equine/Xinjiang/5/2007 | | | EPI_ISL_32666 | | EPI186931 | | | |  |
| A/equine/Xinjiang/4/2007 | | | EPI_ISL_32665 | | EPI186923 | | | |  |
| A/equine/Xinjiang/3/2007 | | | EPI_ISL_32664 | | EPI186915 | | | |  |
| A/equine/Xinjiang/2/2007 | | | EPI_ISL_32663 | | EPI186907 | | | |  |
| A/equine/Xinjiang/1/2007 | | | EPI_ISL_32662 | | EPI186899 | | | |  |
| A/equine/Wisconsin/1/03 | | | EPI_ISL_9783 | | EPI99392 | | | |  |
| A/equine/Virginia/131054-3/2005 | | | EPI_ISL_79899 | | EPI281592 | | | |  |
| A/equine/Uruguay/1/1963 | | | EPI_ISL_21282 | | EPI154033 | | | |  |
| A/equine/Tottori/1/07 | | | EPI_ISL_89221 | | EPI314454 | | | |  |
| A/equine/Texas/39655/1991 | | | EPI_ISL_15349 | | EPI133197 | | | |  |
| A/equine/Texas/117793/2005 | | | EPI_ISL_79867 | | EPI281336 | | | |  |
| A/equine/Texas/117793/2005 | | | EPI_ISL_79867 | | EPI281336 | | | |  |
| A/equine/Tennessee/5/1986 | | | EPI_ISL_21061 | | EPI133254 | | | |  |
| A/equine/Tennessee/4A/2014 | | | EPI_ISL_220481 | | EPI753430 | | | |  |
| A/equine/Tennessee/30A/2014 | | | EPI_ISL_220482 | | EPI753462 | | | |  |
| A/equine/Tennessee/29A/2014 | | | EPI_ISL_220485 | | EPI753454 | | | |  |
| A/equine/Tennessee/28B/2014 | | | EPI_ISL_220484 | | EPI753448 | | | |  |
| A/equine/Tennessee/28A/2014 | | | EPI_ISL_220480 | | EPI753467 | | | |  |
| A/equine/Tennessee/27A/2014 | | | EPI_ISL_220483 | | EPI753442 | | | |  |
| A/equine/Switzerland/P112/07 | | | EPI_ISL_177468 | | EPI584111 | | | |  |
| A/equine/Switzerland/2225/1979 | | | EPI_ISL_22611 | | EPI159279 | | | |  |
| A/equine/Switzerland/173/1993 | | | EPI_ISL_19676 | | EPI153969 | | | |  |
| A/equine/Switzerland/1118/1979 | | | EPI_ISL_19679 | | EPI153993 | | | |  |
| A/Equine/Sweden/SVA111206SZ0085/VIR165837/2011 | | | EPI_ISL_180684 | | EPI594130 | | | |  |
| A/equine/Sussex/93753/89 | | | EPI_ISL_8280 | | EPI154467 | | | |  |
| A/equine/Sussex/1/1989 | | | EPI_ISL_19884 | | EPI240130 | | | |  |
| A/equine/Sussex/1/1989 | | | EPI_ISL_19884 | | EPI154459 | | | |  |
| A/equine/South Kazakhstan/236/2012 | | | EPI_ISL_166607 | | EPI542626 | | | |  |
| A/Equine/South Africa/4/03 | | | EPI_ISL_240665 | | EPI873602 | | | |  |
| A/equine/Snailwell/98 | | | EPI_ISL_177489 | | EPI584184 | | | |  |
| A/equine/Shropshire/10 | | | EPI_ISL_156748 | | EPI584224 | | | |  |
| A/equine/Saone-et-Loire/1/2015 | | | EPI_ISL_257683 | | EPI969075 | | | |  |
| A/equine/Sao Paulo/6/1963 | | | EPI_ISL_19670 | | EPI153921 | | | |  |
| A/equine/Sao Paulo/1/1969 | | | EPI_ISL_19681 | | EPI154009 | | | |  |
| A/equine/Sao Paulo/1.19/2012 | | | EPI_ISL_279065 | | EPI1065029 | | | |  |
| A/equine/Santa Fe/1/1985 | | | EPI_ISL_19672 | | EPI153937 | | | |  |
| A/equine/Sachiyama/1/1971 | | | EPI_ISL_25008 | | EPI165408 | | | |  |
| A/equine/Romania/1/1980 | | | EPI_ISL_19680 | | EPI154001 | | | |  |
| A/equine/Roma/5/1991 | | | EPI_ISL_19677 | | EPI153977 | | | |  |
| A/equine/Rio Grande do Sul/1/12 | | | EPI_ISL_177502 | | EPI584292 | | | |  |
| A/equine/Richmond/1/2007 | | | EPI_ISL_153499 | | EPI501396 | | | |  |
| A/equine/Qinghai/1/1994 | | | EPI_ISL_32661 | | EPI186891 | | | |  |
| A/equine/Perthshire/3/09 | | | EPI_ISL_177492 | | EPI584208 | | | |  |
| A/equine/Oregon/78356/2012 | | | EPI_ISL_294934 | | EPI1157805 | | | |  |
| A/equine/Ohio/113461-3/2005 | | | EPI_ISL_79870 | | EPI281360 | | | |  |
| A/equine/Ohio/113461-2/2005 | | | EPI_ISL_79866 | | EPI281328 | | | |  |
| A/equine/Ohio/113461-2/2005 | | | EPI_ISL_79866 | | EPI281328 | | | |  |
| A/equine/Ohio/113461-1/2005 | | | EPI_ISL_79869 | | EPI281352 | | | |  |
| A/equine/Ohio/1/2003 | | | EPI_ISL_9534 | | EPI98527 | | | |  |
| A/equine/Northamptonshire/1/2013 | | | EPI_ISL_151841 | | EPI584249 | | | |  |
| A/equine/Newmarket/5/2003 | | | EPI_ISL_30894 | | EPI235162 | | | |  |
| A/equine/Newmarket/2/1993 | | | EPI_ISL_30896 | | EPI239740 | | | |  |
| A/equine/Newmarket/1/1993 | | | EPI_ISL_30895 | | EPI239734 | | | |  |
| A/equine/New York/VR-297/1983 | | | EPI_ISL_8102 | | EPI78012 | | | |  |
| A/equine/New York/452/2003 | | | EPI_ISL_9532 | | EPI98533 | | | |  |
| A/equine/New York/146066/2007 | | | EPI_ISL_79900 | | EPI281600 | | | |  |
| A/equine/New York/135857/2016 | | | EPI_ISL_294936 | | EPI1157810 | | | |  |
| A/equine/New York/1/1983 | | | EPI_ISL_15356 | | EPI133349 | | | |  |
| A/equine/New Market/nasalwash1/1979 | | | EPI_ISL_95198 | | EPI332921 | | | |  |
| A/equine/New Market/1/1979 | | | EPI_ISL_95199 | | EPI332926 | | | |  |
| A/equine/Neuville-Pres-Sees/1/2011 | | | EPI_ISL_257679 | | EPI969072 | | | |  |
| A/equine/Mysore/1/2008 | | | EPI_ISL_74367 | | EPI580100 | | | |  |
| A/equine/Moulton/98 | | | EPI_ISL_177487 | | EPI584169 | | | |  |
| A/equine/Montana/9564-1/2015 | | | EPI_ISL_285417 | | EPI1103403 | | | |  |
| A/equine/Montana/9233/2007 | | | EPI_ISL_79893 | | EPI281544 | | | |  |
| A/equine/Miami/1/1963 | | | EPI_ISL_8092 | | EPI77822 | | | |  |
| A/equine/Massachussetts/213/2003 | | | EPI_ISL_9529 | | EPI98535 | | | |  |
| A/equine/Malaysia/M201/2015 | | | EPI_ISL_201434 | | EPI1073180 | | | |  |
| A/equine/Lincolnshire/1/2007 | | | EPI_ISL_29831 | | EPI501401 | | | |  |
| A/equine/Lincolnshire/06 | | | EPI_ISL_177471 | | EPI584124 | | | |  |
| A/equine/Liaoning/9/2008 | | | EPI_ISL_32659 | | EPI186875 | | | |  |
| A/equine/Lanarkshire/09 | | | EPI_ISL_177490 | | EPI584192 | | | |  |
| A/equine/Kyonggi/SA1/2011 | | | EPI_ISL_129971 | | EPI398884 | | | |  |
| A/equine/Kentucky/Rosie100/1981 | | | EPI_ISL_15830 | | EPI137545 | | | |  |
| A/equine/Kentucky/pass_the_pepper1/1976 | | | EPI_ISL_8093 | | EPI77841 | | | |  |
| A/equine/Kentucky/magnificent_genius1/1981 | | | EPI_ISL_8100 | | EPI77974 | | | |  |
| A/equine/Kentucky/bitter_boredom5/1976 | | | EPI_ISL_8094 | | EPI77860 | | | |  |
| A/equine/Kentucky/8/1994 | | | EPI_ISL_15353 | | EPI133292 | | | |  |
| A/equine/Kentucky/698/1988 | | | EPI_ISL_15347 | | EPI133159 | | | |  |
| A/equine/Kentucky/694/1988 | | | EPI_ISL_15346 | | EPI133140 | | | |  |
| A/equine/Kentucky/692/1988 | | | EPI_ISL_15345 | | EPI133121 | | | |  |
| A/equine/Kentucky/5/2002 | | | EPI_ISL_9530 | | EPI98529 | | | |  |
| A/equine/Kentucky/5/02 | | | EPI_ISL_4663 | | EPI25736 | | | |  |
| A/equine/Kentucky/4/1980 | | | EPI_ISL_8088 | | EPI77746 | | | |  |
| A/equine/Kentucky/3/1986 | | | EPI_ISL_19663 | | EPI153857 | | | |  |
| A/equine/Kentucky/3/1981 | | | EPI_ISL_15342 | | EPI133045 | | | |  |
| A/equine/Kentucky/211/1987 | | | EPI_ISL_68958 | | EPI240124 | | | |  |
| A/equine/Kentucky/2/1987 | | | EPI_ISL_19104 | | EPI152776 | | | |  |
| A/equine/Kentucky/2/1986 | | | EPI_ISL_14705 | | EPI130339 | | | |  |
| A/equine/Kentucky/2/1986 | | | EPI_ISL_14705 | | EPI130339 | | | |  |
| A/equine/Kentucky/2/1981 | | | EPI_ISL_8090 | | EPI77784 | | | |  |
| A/equine/Kentucky/2/1980 | | | EPI_ISL_20807 | | EPI156248 | | | |  |
| A/equine/Kentucky/1277/1990 | | | EPI_ISL_15348 | | EPI133178 | | | |  |
| A/equine/Kentucky/1/1992 | | | EPI_ISL_15350 | | EPI133216 | | | |  |
| A/equine/Kentucky/1/1991 | | | EPI_ISL_15352 | | EPI133273 | | | |  |
| A/equine/Kentucky/1/1987 | | | EPI_ISL_21060 | | EPI133102 | | | |  |
| A/equine/Kentucky/1/1986 | | | EPI_ISL_15344 | | EPI133083 | | | |  |
| A/equine/Kentucky/1/1981 | | | EPI_ISL_68957 | | EPI240117 | | | |  |
| A/equine/Kentucky/1/1978 | | | EPI_ISL_8087 | | EPI77727 | | | |  |
| A/equine/Kentucky/1/11 | | | EPI_ISL_177500 | | EPI584275 | | | |  |
| A/Equine/Kent/1/15 | | | EPI_ISL_240663 | | EPI873586 | | | |  |
| A/equine/Katra-Jammu/6/2008 | | | EPI_ISL_154972 | | EPI580099 | | | |  |
| A/equine/Kascakew/1/1978 | | | EPI_ISL_69898 | | EPI242350 | | | |  |
| A/equine/Jouars/4/2006 | | | EPI_ISL_257684 | | EPI969070 | | | |  |
| A/equine/Johannesburg/1/1986 | | | EPI_ISL_20809 | | EPI156264 | | | |  |
| A/equine/Italy/824/1991 | | | EPI_ISL_19673 | | EPI153945 | | | |  |
| A/equine/Italy/1199/1992 | | | EPI_ISL_19674 | | EPI153953 | | | |  |
| A/equine/Italy/1062/1991 | | | EPI_ISL_19678 | | EPI153985 | | | |  |
| A/equine/Inner Mongolia/8/2008 | | | EPI_ISL_32660 | | EPI186883 | | | |  |
| A/equine/Idaho/37875/1991 | | | EPI_ISL_6623 | | EPI52084 | | | |  |
| A/equine/Ibaraki/1/2007 | | | EPI_ISL_297956 | | EPI1172959 | | | |  |
| A/equine/Hubei/6/2008 | | | EPI_ISL_32657 | | EPI186859 | | | |  |
| A/equine/Huabei/1/2007 | | | EPI_ISL_70195 | | EPI243544 | | | |  |
| A/equine/Himachal Pradesh/CMVL-YOL2/2008 | | | EPI_ISL_190904 | | EPI621741 | | | |  |
| A/equine/Heilongjiang/SS1/2013 | | | EPI_ISL_161474 | | EPI527599 | | | |  |
| A/equine/Heilongjiang/10/2008 | | | EPI_ISL_32658 | | EPI186867 | | | |  |
| A/equine/Heilongjiang/1/2010 | | | EPI_ISL_144923 | | EPI464973 | | | |  |
| A/Equine/Hampshire/4/2016 | | | EPI_ISL_240664 | | EPI873594 | | | |  |
| A/equine/Guangxi/1/2008 | | | EPI_ISL_174545 | | EPI573746 | | | |  |
| A/equine/Gironde/1/2014 | | | EPI_ISL_257682 | | EPI969074 | | | |  |
| A/equine/Georgia/9/1981 | | | EPI_ISL_8097 | | EPI77917 | | | |  |
| A/equine/Georgia/3/1981 | | | EPI_ISL_8096 | | EPI77898 | | | |  |
| A/equine/Georgia/13/1981 | | | EPI_ISL_8099 | | EPI77955 | | | |  |
| A/equine/Georgia/121362-16/2016 | | | EPI_ISL_294939 | | EPI1157892 | | | |  |
| A/equine/Georgia/10/1981 | | | EPI_ISL_8098 | | EPI77936 | | | |  |
| A/equine/Georgia/1/1981 | | | EPI_ISL_8095 | | EPI77879 | | | |  |
| A/equine/Gansu/7/2008 | | | EPI_ISL_32656 | | EPI186851 | | | |  |
| A/equine/France/1/1967 | | | EPI_ISL_22612 | | EPI159287 | | | |  |
| A/equine/Fontainbleu/1/1979 | | | EPI_ISL_19682 | | EPI154017 | | | |  |
| A/equine/Florida/146609/2011 | | | EPI_ISL_294944 | | EPI1157884 | | | |  |
| A/Equine/East Sussex/1/2015 | | | EPI_ISL_240662 | | EPI873607 | | | |  |
| A/equine/East Renfrewshire/2/11 | | | EPI_ISL_156750 | | EPI584233 | | | |  |
| A/equine/Dubai/1/12 | | | EPI_ISL_177501 | | EPI584283 | | | |  |
| A/equine/Dorset/09 | | | EPI_ISL_177491 | | EPI584200 | | | |  |
| A/equine/Devon/1/11 | | | EPI_ISL_156749 | | EPI584241 | | | |  |
| A/equine/Cordoba/18/1985 | | | EPI_ISL_19671 | | EPI153929 | | | |  |
| A/equine/Cheshire/06 | | | EPI_ISL_177472 | | EPI584132 | | | |  |
| A/equine/Cambremer/1/2012 | | | EPI_ISL_257680 | | EPI969073 | | | |  |
| A/equine/California/8560/2002 | | | EPI_ISL_15843 | | EPI137792 | | | |  |
| A/equine/California/83/1982 | | | EPI_ISL_15343 | | EPI133064 | | | |  |
| A/equine/California/4537/1997 | | | EPI_ISL_15842 | | EPI137773 | | | |  |
| A/equine/California/191/2003 | | | EPI_ISL_9533 | | EPI98537 | | | |  |
| A/equine/California/103/1982 | | | EPI_ISL_8101 | | EPI77993 | | | |  |
| A/equine/California/1/1980 | | | EPI_ISL_8089 | | EPI77765 | | | |  |
| A/equine/California/1/10 | | | EPI_ISL_177498 | | EPI584266 | | | |  |
| A/equine/Buckinghamshire/1/2014 | | | EPI_ISL_197753 | | EPI651396 | | | |  |
| A/equine/Berlin/1/1989 | | | EPI_ISL_19683 | | EPI154025 | | | |  |
| A/equine/Belfond/6-2/2009 | | | EPI_ISL_257677 | | EPI969071 | | | |  |
| A/equine/Austria/421/1992 | | | EPI_ISL_19675 | | EPI153961 | | | |  |
| A/equine/Ankara/1/2013 | | | EPI_ISL_281349 | | EPI1076903 | | | |  |
| A/equine/Algiers/1/1972 | | | EPI_ISL_20808 | | EPI156256 | | | |  |
| A/equine/Alaska/29759/1991 | | | EPI_ISL_15351 | | EPI133235 | | | |  |
| A/equine/Ahmedabad/1/2009 | | | EPI_ISL_74364 | | EPI580101 | | | |  |
| A/donkey/Shandong/1/2017 | | | EPI_ISL_284681 | | EPI1098477 | | | |  |
| A/canine/VT/11039/2013 | | | EPI_ISL_166505 | | EPI546871 | | | |  |
| A/canine/Vermont/278213/2013 | | | EPI_ISL_294941 | | EPI1157883 | | | |  |
| A/canine/Pennsylvania/96978/2009 | | | EPI_ISL_294937 | | EPI1157845 | | | |  |
| A/canine/Pennsylvania/94930-3/2007 | | | EPI_ISL_79897 | | EPI281576 | | | |  |
| A/canine/Pennsylvania/16699/2007 | | | EPI_ISL_79890 | | EPI281520 | | | |  |
| A/canine/Pennsylvania/137154/2008 | | | EPI_ISL_79903 | | EPI281624 | | | |  |
| A/canine/Pennsylvania/10915/2007 | | | EPI_ISL_79892 | | EPI281536 | | | |  |
| A/canine/Pennsylvania/10909/2007 | | | EPI_ISL_79891 | | EPI281528 | | | |  |
| A/canine/PA/33225.4/2010 | | | EPI_ISL_167696 | | EPI546870 | | | |  |
| A/canine/PA/111788.2/2009 | | | EPI_ISL_166506 | | EPI546872 | | | |  |
| A/canine/NY/dog4c13/2009 | | | EPI_ISL_82009 | | EPI289617 | | | |  |
| A/canine/NY/dog4c07/2009 | | | EPI_ISL_82085 | | EPI289616 | | | |  |
| A/canine/NY/dog4c06/2009 | | | EPI_ISL_82012 | | EPI289618 | | | |  |
| A/canine/NY/dog4/2009 | | | EPI_ISL_82093 | | EPI289220 | | | |  |
| A/canine/NY/dog3c15/2009 | | | EPI_ISL_82144 | | EPI289619 | | | |  |
| A/canine/NY/dog23/2009 | | | EPI_ISL_82094 | | EPI289221 | | | |  |
| A/canine/NY/120106.2/2011 | | | EPI_ISL_166503 | | EPI546869 | | | |  |
| A/canine/NY/105447/2008 | | | EPI_ISL_174477 | | EPI576526 | | | |  |
| A/canine/New York/51854/2008 | | | EPI_ISL_79902 | | EPI281616 | | | |  |
| A/canine/New York/5183-6/2006 | | | EPI_ISL_79871 | | EPI281368 | | | |  |
| A/canine/New York/5183-6/2006 | | | EPI_ISL_79871 | | EPI281368 | | | |  |
| A/canine/New York/4986-2/2006 | | | EPI_ISL_79873 | | EPI281384 | | | |  |
| A/canine/New York/3699/2010 | | | EPI_ISL_294940 | | EPI1157873 | | | |  |
| A/canine/New York/1623.1/2010 | | | EPI_ISL_294943 | | EPI1157824 | | | |  |
| A/canine/New York/159903/2012 | | | EPI_ISL_294938 | | EPI1157879 | | | |  |
| A/canine/New York/158402-1/2008 | | | EPI_ISL_79905 | | EPI281640 | | | |  |
| A/canine/New York/147926-5/2006 | | | EPI_ISL_79888 | | EPI281504 | | | |  |
| A/canine/New York/147926-3/2006 | | | EPI_ISL_79887 | | EPI281496 | | | |  |
| A/canine/New York/145353/2008 | | | EPI_ISL_79904 | | EPI281632 | | | |  |
| A/canine/New York/115809/2005 | | | EPI_ISL_79864 | | EPI281312 | | | |  |
| A/canine/New York/115719/2007 | | | EPI_ISL_79896 | | EPI281568 | | | |  |
| A/canine/New York/100528-6/2006 | | | EPI_ISL_79886 | | EPI281488 | | | |  |
| A/canine/New York/100528-5/2006 | | | EPI_ISL_79885 | | EPI281480 | | | |  |
| A/canine/New York/100528-1/2006 | | | EPI_ISL_79884 | | EPI281472 | | | |  |
| A/canine/New York/100525-1/2006 | | | EPI_ISL_79883 | | EPI281464 | | | |  |
| A/canine/Miami/2005 | | | EPI_ISL_64819 | | EPI226913 | | | |  |
| A/canine/Massachusetts/26810/2016 | | | EPI_ISL_234612 | | EPI839119 | | | |  |
| A/canine/Maine/058124/2016 | | | EPI_ISL_294942 | | EPI1157828 | | | |  |
| A/canine/Kentucky/118778/2006 | | | EPI_ISL_79889 | | EPI281512 | | | |  |
| A/canine/Jacksonville/2005 | | | EPI_ISL_64818 | | EPI226907 | | | |  |
| A/canine/Florida/89911-2/2006 | | | EPI_ISL_79882 | | EPI281456 | | | |  |
| A/canine/Florida/78592-7/2006 | | | EPI_ISL_79881 | | EPI281448 | | | |  |
| A/canine/Florida/78592-6/2006 | | | EPI_ISL_79880 | | EPI281440 | | | |  |
| A/canine/Florida/78592-2/2006 | | | EPI_ISL_79879 | | EPI281432 | | | |  |
| A/canine/Florida/61156-2/2006 | | | EPI_ISL_79895 | | EPI281560 | | | |  |
| A/canine/Florida/61156-2/2006 | | | EPI_ISL_79895 | | EPI281560 | | | |  |
| A/canine/Florida/43/2004 | | | EPI_ISL_9527 | | EPI98461 | | | |  |
| A/canine/Florida/242/2003 | | | EPI_ISL_9528 | | EPI98477 | | | |  |
| A/canine/Florida/15592.1/2004 | | | EPI_ISL_294945 | | EPI1157877 | | | |  |
| A/canine/Connecticut/85863/2011 | | | EPI_ISL_294935 | | EPI1157823 | | | |  |
| A/canine/Colorado/8880/2006 | | | EPI_ISL_79874 | | EPI281392 | | | |  |
| A/canine/Colorado/6723-8/2008 | | | EPI_ISL_79901 | | EPI281608 | | | |  |
| A/canine/Colorado/6723-8/2008 | | | EPI_ISL_79901 | | EPI281608 | | | |  |
| A/canine/Colorado/30604/2006 | | | EPI_ISL_79877 | | EPI281416 | | | |  |
| A/canine/Colorado/17864/2006 | | | EPI_ISL_79875 | | EPI281400 | | | |  |
| A/canine/Colorado/17864/2006 | | | EPI_ISL_79875 | | EPI281400 | | | |  |
| A/canine/California/70645-4/2006 | | | EPI_ISL_79878 | | EPI281424 | | | |  |
