# Supplementary figures and images for: Host-range shift of H3N8 canine influenza virus: a phylodynamic analysis of its origin and adaptation from equine to canine host
Source: Vet Res. 2019 Oct 30;50:87. doi: 10.1186/s13567-019-0707-2 (PMC6822366; doi:10.1186/s13567-019-0707-2)

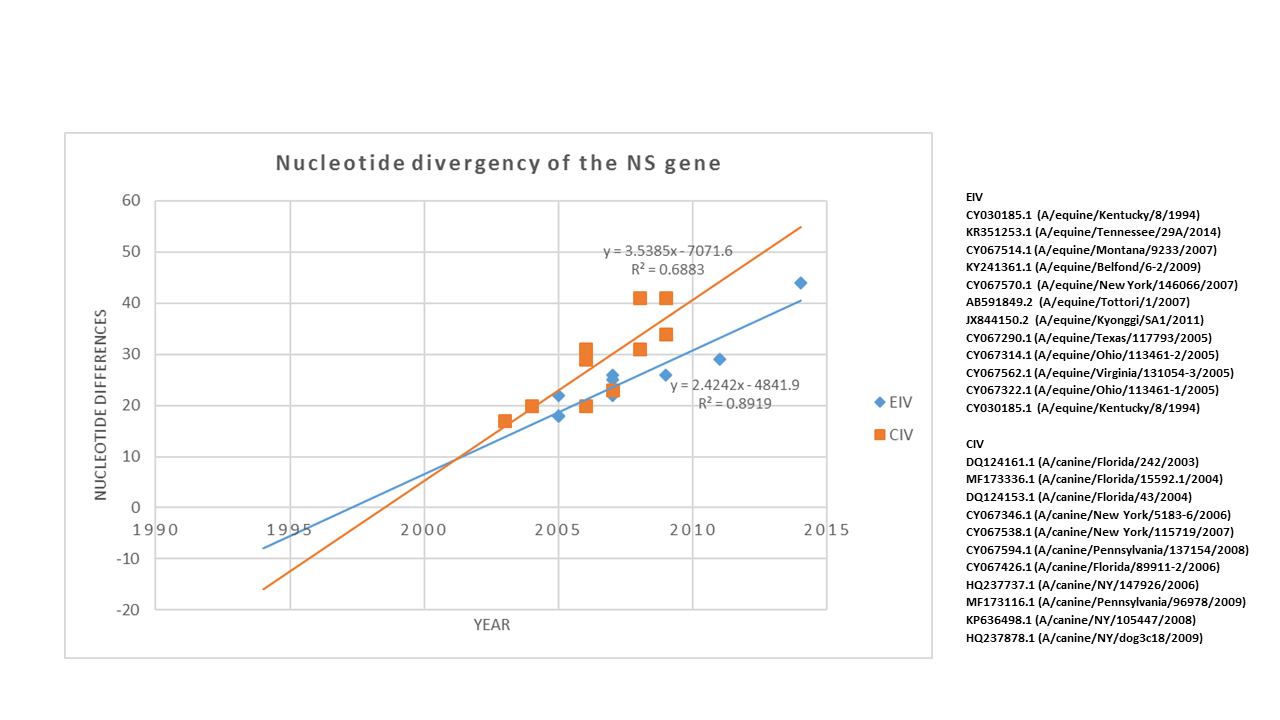

Supplement: Supplementary file 2 — Additional file 2. Regression analysis of the rate of nucleotide substitutions for the NS1 gene of EIV and CIV. [file 13567_2019_707_MOESM2_ESM.tif]
