# Supplementary material for: Host-range shift of H3N8 canine influenza virus: a phylodynamic analysis of its origin and adaptation from equine to canine host
Source: Vet Res. 2019 Oct 30;50:87. doi: 10.1186/s13567-019-0707-2 (PMC6822366; doi:10.1186/s13567-019-0707-2)
Supplement: Supplementary file 3 — Additional file 3. Amino acid changes that differentiate H3N8 CIV into six clades. [file 13567_2019_707_MOESM3_ESM.docx]

| HA | Emerging | clade I | clade II | clade III | clade IV | clade V | clade VI |
| --- | --- | --- | --- | --- | --- | --- | --- |
| 41 | V | V | V | V | V | I | V |
| 73 | I | I | I | I | I | V | I |
| 90 | H | H | H | H | H | Q | H |
| 107 | S | N | S | N | N | N | N |
| 133 | L | L | V | V | V | V | V |
| 150 | R | R | R | R | K | R | R |
| 187 | K | K | K | K | E | K | K |
| 190 | D | D | D | D | E | D | D |
| 197 | I | I | I | I | V | I | I |
| 231 | N | N | N | N | H | H | H |
| 238 | V | V | V | V | I | I | V |
| 276 | K | K | N | N | N | N | N |
| 277 | T | T | T | T | T | P | T |
| 283 | M | M | M | M | M | I | M |
| 325 | R | R | R | R | R | K | R |
| 402 | R | R | R | R | K | R | R |
| 479 | G | G | D | D | D | N | G |
| 494 | G | G | E | E | E | E | E |
| 511 | L | L | L | L | L | I | L |
| 556 | K | R | K | K | K | K | K |
| M1 | Emerging | clade I | clade II | clade III | clade IV | clade V | clade VI |
| 15 | V | V | V | I | I | I | I |
| 85 | S | S | S | S | S | N | S |
| 138 | V | V | V | I | I | I | I |
| 147 | V | V | V | V | I | V | V |
| 161 | S | A | S | S | S | S | S |
| NA | Emerging | clade I | clade II | clade III | clade IV | clade V | clade VI |
| 7 | I | I | I | I | V | I | I |
| 12 | F | F | F | F | S | S | S |
| 22 | V | V | V | V | V | I | V |
| 35 | V | V | V | V | V | I | V |
| 45 | C | C | C | Y | C | C | C |
| 62 | I | I | L | L | L | L | L |
| 63 | T | T | I | T | T | T | T |
| 68 | T | T | T | T | T | I | T |
| 71 | I | I | I | I | I | T | I |
| 147 | V | V | I | I | I | I | I |
| 171 | R | R | R | R | K | K | K |
| 201 | I | I | T | I | I | I | I |
| 209 | V | V | V | V | V | I | V |
| 247 | R | R | S | R | R | R | R |
| 250 | K | K | K | K | N | N | N |
| 301 | I | V | I | I | I | I | I |
| 332 | T | T | T | T | P | T | T |
| 337 | S | S | N | S | S | S | S |
| 365 | R | R | K | R | R | R | R |
| 452 | I | I | I | I | V | I | I |
| NP | Emerging | clade I | clade II | clade III | clade IV | clade V | clade VI |
| 27 | A | A | A | A | T | T | T |
| 52 | H | H | H | H | H | Y | H |
| 186 | V | V | V | I | V | V | V |
| 214 | R | K | R | R | R | R | R |
| 285 | V | I | V | V | V | V | V |
| 313 | F | F | L | F | F | F | F |
| 357 | Q | Q | Q | H | Q | Q | Q |
| 498 | S | S | S | S | N | N | N |
| NS | Emerging | clade I | clade II | clade III | clade IV | clade V | clade VI |
| 21 | R | R | R | R | Q | Q | R |
| 50 | L | L | I | L | L | L | L |
| 72 | E | E | E | E | E | E | K |
| 77 | L | L | L | L | P | P | P |
| 86 | T | T | A | A | A | A | A |
| 88 | R | L | R | R | R | R | R |
| 140 | G | G | R | R | R | R | R |
| 156 | V | V | V | V | I | I | I |
| 185 | L | L | L | L | F | F | F |
| 193 | R | R | R | R | R | K | R |
| 212 | P | P | H | P | P | S | P |
| 214 | F | F | F | F | F | L | F |
| PA | Emerging | clade I | clade II | clade III | clade IV | clade V | clade VI |
| 58 | G | G | S | G | G | G | G |
| 142 | K | K | K | K | K | R | K |
| 184 | G | G | S | G | G | G | S |
| 233 | V | V | I | I | V | V | V |
| 256 | R | R | K | K | K | K | K |
| 308 | I | I | I | I | V | I | I |
| 327 | E | E | K | K | K | K | K |
| 348 | L | L | L | L | I | I | I |
| 353 | R | R | R | R | K | K | R/K |
| 400 | A | A | A | A | A | V/I | A |
| 409 | S | S | S | S | S | N | S/N |
| 437 | Y | Y | Y | Y | Y | H | Y |
| 441 | M | M | M | M | M | I | M |
| 444 | N | N | N | N | D | D | D |
| 460 | M | I | M | M | M | M | M |
| 603 | K | K | K | K | K | R | K |
| 673 | R | K | R | R | R | R | R |
| PB1 | Emerging | clade I | clade II | clade III | clade IV | clade V | clade VI |
| 119 | V | V | M | V | V | V | V |
| 164 | M | M | M | M | I | I | I |
| 191 | V | V | I | V | V | V | V |
| 200 | V | V | I | I | I | I | I |
| 213 | N | N | S | N | N | N | N |
| 338 | S | S | S | S | N | N | N |
| 398 | D | D | D | D | N | N | N |
| 459 | I | V | I | I | I | I | I |
| 529 | V | V | V | V | I | T | V |
| 584 | R | R | R | R | Q | Q | Q |
| 591 | V | V | V | V | I | I | V |
| 682 | I | V | I | I | I | I | I |
| 687 | Q | Q | Q | Q | H | H | H |
| 754 | R | R | R | R | K | K | R/K |
| PB2 | Emerging | clade I | clade II | clade III | clade IV | clade V | clade VI |
| 37 | G | E | G | G | G | G | G |
| 81 | T | T | T | T | T | M | T |
| 91 | V | V | V | V | I | V | V |
| 107 | S | S | N | N | N | N | N |
| 152 | A | A | A | A | S | A | A |
| 175 | R | I | R | R | R | R | R |
| 221 | A | A | V | V | V | V | V |
| 227 | V | V | V | V | I | I | V |
| 251 | R | R | R | R | K | R | R |
| 292 | I | I | T | T | T | T | T |
| 295 | V | V | V | V | V | I | V |
| 344 | M | M | M | M | M | L | M |
| 389 | R | R | R | R | K | K | R |
| 447 | Q | H | Q | Q | Q | Q | Q |
| 457 | V | V | V | V | I | V | V |
| 559 | I | I | I | N | N | N | N |
| 661 | A | A | V | A | A | A | A |
| 679 | P | P | S | P | P | P | P |
| 731 | V | V | V | V | V | I | V |
